# Precision medicine for Alzheimer's disease in Down syndrome

**DOI:** 10.1002/alz.71398

**Published:** 2026-04-19

**Authors:** Sonal Sukreet, Elise K. Kim, Melissa Petersen, Fan Zhang, Sid E. O'Bryant, Michael S. Rafii, Robert A. Rissman

**Affiliations:** ^1^ Departments of Physiology and Neuroscience Keck School of Medicine of the University of Southern California San Diego California USA; ^2^ Institute for Translational Research Department of Family Medicine University of North Texas Health Science Center Fort Worth Texas USA; ^3^ Department of Neurology Keck School of Medicine of the University of Southern California San Diego California USA

**Keywords:** Alzheimer's disease, blood‐based biomarkers, Down syndrome, endosomal dysfunction, precision therapy, small extracellular vesicles

## Abstract

**INTRODUCTION:**

Down syndrome (DS) exhibits a genetic form of Alzheimer's disease (AD). We used a blood‐based proteomic algorithm to predict cognitive status, treatment responders, and change to vitamin E in DS adults from a completed clinical trial, “Vitamin E in Aged Persons with Down Syndrome,” which originally showed no significant cognitive benefit using the primary endpoint cognition (Brief Praxis Test [BPT]).

**METHODS:**

Plasma and extracellular vesicle (EV; astrocytic and neuronal) biomarkers were assayed at baseline and 36 months (*n* = 138 each). Cognitive response was measured using combined scores from the BPT, vocabulary, and behavior and function DS tests. Support vector machine (SVM) analyses predicted diagnostic and treatment responders and change accuracy.

**RESULTS:**

SVM classified demented versus non‐demented with up to 99% accuracy and predicted treatment response and changes with up to 100% accuracy in plasma and EV.

**DISCUSSION:**

Our study supports blood‐based screening and precision diagnostics for AD therapy in DS.

## Highlights

- Plasma and plasma‐derived astrocytic and neuronal extracellular vesicles (EVs) predict dementia status in adults with Down syndrome (DS).
- Proinflammatory and Alzheimer's disease (AD)‐related proteins identify responders to vitamin E treatment.
- Plasma and EV proteomics distinguish responders from non‐responders over 36 months.
- Longitudinal biomarker shifts track change in treatment in DS‐related AD.
- Our study supports scalable screening and precision medicine tools for ongoing and future DS‐associated AD clinical trials.

## 1 INTRODUCTION

Down syndrome (DS) is a common genetic disorder that affects 1 in 700 babies.^1^ DS individuals are at risk for Alzheimer's disease (AD) due to chromosome 21 trisomy, causing overexpression of amyloid precursor protein (APP).^2^, ^3^ Advances in early interventions and medication now allow individuals with DS to reach adulthood, and nearly all develop amyloid beta (Aβ) plaques by age 40, offering an opportunity to inform AD research and treatment development.^4^, ^5^, ^6^ Detecting Aβ and tau is invasive, costly, and physically challenging for adults with DS, who need a multitiered approach to assess and predict AD risk for targeted interventions.^7^ The amyloid/tau/neurodegeneration (A/T/N) framework has transformed AD research and trials, but remains underexplored in adults with DS despite its promise for tailored therapies in AD.^8^, ^9^, ^10^, ^11^, ^12^, ^13^, ^14^, ^15^, ^16^ Higher plasma Aβ42 levels in DS have been associated with the presence of one apolipoprotein E (*APOE*) ε4 allele and increased dementia prevalence, as well as elevated risk of AD and mortality in non‐demented (ND) individuals.^12^, ^13^ Dementia risk has been linked to longitudinal fluctuations and elevated levels of plasma Aβ42 and Aβ40, with Aβ and tau proteins in cerebrospinal fluid (CSF) and brain tissue.^14^, ^17^, ^18^, ^19^, ^20^, ^21^ Studies show plasma phosphorylated tau217 (p‐tau217) accurately distinguishes stable, prodromal, and dementia stages, strongly correlating with Aβ status in adults with DS (area under the curve [AUC] ≈ 0.95–0.96).^22^, ^23^, ^24^ Complementary research in AD cohorts indicates that plasma Aβ42/40 ratios, p‐tau181, glial fibrillary acidic protein (GFAP), and neurofilament light chain (NfL) can differentiate AD from other dementias and controls, supporting their role as accessible biomarkers for disease staging and progression.^25^, ^26^ Blood‐based and imaging biomarkers in this population demonstrate similar diagnostic performance and temporal changes to sporadic and autosomal dominant AD.^20^, ^21^

Vitamin E (vit E), a lipophilic antioxidant and anti‐inflammatory, was proposed for AD due to its ability to cross the blood–brain barrier and reduce oxidative stress (OS) and inflammation.^27^, ^28^ Clinical trials on vit E in AD show mixed results; some report slowed progression, while others found no benefit.^29^, ^30^, ^31^, ^32^ Given the well‐documented OS in DS and its mechanistic similarities to AD pathology, vit E was considered a plausible intervention. However, despite this logical basis, its therapeutic effectiveness in DS remains uncertain, as current clinical evidence has not definitively demonstrated preventive or disease‐modifying benefits, with one trial indicating no AD‐prevention benefit in DS.^33^ However, the trial did not use blood‐based biomarker assays to assess AD progression, which may have limited the evaluation of treatment efficacy. Recent advances in biomarkers in plasma, astrocytic, and neuronal small extracellular vesicles (aEVs and nEVs) have opened up new avenues for DS‐associated AD (DSAD) to visualize the time it takes to detect pathological proteins in EV compared to plasma.^34^, ^35^, ^36^, ^37^, ^38^

For this study, we used biobanked plasma samples from a clinical trial on vit E in DS, which showed no cognitive benefit based on the Brief Praxis Test (BPT); the primary endpoint evaluated cognitive decline, motor‐related changes, intellectual development, and age‐related change.^33^, ^39^ Cognitive and functional variability in DS challenges standardized trial criteria, making tailored patient selection crucial for the DSAD trials. We propose targeted therapeutics for subsets of DS individuals by analyzing plasma and plasma‐derived aEVs and nEVs using an established proteomic profiling for neuroinflammation and AD to identify cognitive status and treatment responders. Unlike traditional trials using “trial and error” methods, precision medicine uses biomarker‐guided strategies for accurate diagnostics and treatment.^40^ Our group has developed and cross‐validated a blood‐based proteomic profile with high accuracy for detecting dementia and treatment responders in AD, and presented proof of concept and efficacy for the precision medicine approach in anti‐inflammatory treatment.^41^, ^42^, ^43^, ^44^, ^45^, ^46^, ^47^ Given inflammatory dysfunction in DS adults with AD, there is a potential that this proteomic profile will help detect ND from AD‐like demented (D) individuals and identify treatment efficacy.

Our work is the first proof of concept precision biomarker framework to compare proinflammatory and AD biomarkers in plasma, aEVs, and nEVs with cognitive status and vit E treatment efficacy in DS adults, with the goal of developing a scalable, cost‐effective screening tool for DS clinical trials targeting neuroinflammation. We hypothesize that EV biomarkers may reveal subtle changes in DS individuals that are not yet detectable in plasma. Cognitive status was assessed using the annual rate of change (ARC) from a combination of BPT, vocabulary test (VT), and behavior and function DS test (BFDS). ARC was selected for its ability to detect longitudinal changes in DS populations, rather than for its use as a diagnostic tool for classification. We evaluated whether baseline biomarkers could predict cognitive status (D vs. ND) and identify treatment responders (yes/no), and whether 36‐month biomarkers could forecast changes from baseline to 36 months.

## 2 METHODS

### 2.1 Participants from the Vitamin E in Aging Persons with Down Syndrome Trial (NCT00056329)

DS patients aged ≥ 50 who spoke English and were medically diagnosed with or without dementia were included in the clinical trial.^33^ A full description of the sample has been previously published.^33^ All clinical experiments followed institutional review board (IRB)‐approved protocols, and patients or their informants provided written informed consent. The plasma samples were received and biobanked at the Rissman laboratory located at University of Southern California Alzheimer's Therapeutic Research Institute, and the trial samples were used for academic research in this investigation. Due to limited sample availability, primarily from misplaced labels, unlabeled samples, and cases in which plasma was unavailable for analysis, only a subset of participants from the original cohort was included in the present analyses. The exclusions were random; nonetheless, we recognize that differences might still exist. That's why this is a proof‐of‐concept study with biomarker‐guided reanalysis of the samples collected in the original vit E trial. From the total enrollment of participants in the vit E clinical trial, after excluding individuals with missing data or samples at baseline, either/or 36 months, the included participants comprised baseline (*n* = 138) and 36‐month (*n* = 138) plasma samples, with *n* = 62 in the placebo and *n* = 76 in the treatment arms.

### 2.2 Total small EV isolation and enrichment of neuronal and astrocyte‐derived EVs from plasma

We applied previously standardized, published protocols from the Rissman laboratory for plasma small EV (sEV) isolation and aEV/nEV enrichment, with minor modifications;^48^ 0.30‐mL plasma aliquots were incubated with 0.1‐mL thromboplastin D (Thermo Fisher Scientific, Inc.) and 0.1‐mL calcium‐ and magnesium‐free 1X phosphate‐buffered saline containing protease inhibitor cocktail (Roche) and phosphatase inhibitor cocktail. Total sEV were precipitated by centrifuging the supernatants at 3000 × g for 30 minutes at 4°C with 120‐µL per tube of ExoQuick (System Biosciences) followed by a spin at 1500 × g for 30 minutes at 4°C. Neuronal and astrocyte enrichment was performed according to the manufacturer's protocol (System Biosciences, Inc.; Cat# CSFLOWBASICA‐1). Briefly, 50 µL of streptavidin magnetic exo‐flow beads were incubated with 100 ng/mL of biotinylated GLAST (ACSA‐1) antibodies or mouse anti‐human CD171 (L1CAM) for 2 hours on ice, mixing every 30 minutes. The bead–antibody complexes were washed three times in bead wash buffer, then suspended in 300 µL of buffer and 100 µL of total sEV suspension and rotated overnight at 4°C. After washing, the complexes were stained with exo‐FITC FACS stain for 2 hours on ice, washed again, and resuspended for sorting using BD FACS Aria II. Flow‐sorted complexes were incubated with elution buffer for 30 minutes at 25°C. Eluted sEV were treated with exo‐FlowIP clearing reagent for 30 minutes at 37°C, aliquoted in centrifuge tubes, and stored at −80°C for downstream experiments. Enriched aEV and nEV were aliquoted and stored at −80°C for downstream experiments. The size and count of aEV and nEV were measured using a NanoFCM (NanoFCM Co., Ltd.) at the Flow Cytometry and Single‐Cell Genomics Core Facilities Center for Biotechnology at the University of Nebraska‐Lincoln, following the manufacturer's instructions. Morphology was assessed using transmission electron microscopy (TEM) and scanning electron microscopy (SEM) at the University of California San Diego Microscopy Core, as previously described.^49^ The enriched aEV and nEV proteins were quantified for sEV transmembrane marker CD81 per the manufacturer's protocol (Cusabio, Cat# CSB‐EL004960HU). The exact numbers of aEV and nEV (≈ 1 x 10^10^ particles) were used to prepare lysed protein samples for proteomic assays, and CD81 mean values were set to 1.00 for each assay group, and relative sample values were normalized accordingly.^48^

**RESEARCH IN CONTEXT**

1. **Systematic review**: We reviewed the literature using PubMed for studies on Alzheimer's disease (AD) biomarkers in individuals with Down syndrome (DS). Although DS is recognized as a genetic model for AD, few studies have examined the utility of predictive blood‐based biomarkers using plasma and plasma‐derived extracellular vesicles (EVs) of both astrocytic and neuronal origin for predicting dementia, treatment responders, and change over time.
2. **Interpretation**: In a vitamin E trial, plasma and EV biomarkers predicted dementia and treatment responders and also tracked treatment effects from baseline in individuals with DS, supporting their use as scalable, non‐invasive clinical trial stratification and monitoring techniques in DS‐associated AD.
3. **Future directions**: Future research should validate these biomarkers in larger, independent cohorts and across diverse stages of cognitive decline in DS. Integrating these blood‐based measures into upcoming clinical trials may enhance the screening process and precision medicine approaches, enabling more targeted neuroinflammation‐modulating interventions in DS.

### 2.3 Proteomic assays

All assays were conducted following laboratory protocols and/or manufacturers’ instructions. The frozen biobanked samples were thawed on ice, and 300 µL of plasma was aliquoted into the two 0.5‐mL polypropylene tubes (Sarstedt 72.706.600) for each participant and immediately stored at −80°C until the assays were conducted. The same number of enriched aEV and nEV particle counts were lysed before the proteomic assay. Mammalian protein extraction reagent (Thermo Fisher Scientific, Catalog #78501), supplemented with protease and phosphatase inhibitors, was used to lyse the eluted nEV and aEV suspensions. Protein concentrations of lysed nEV and aEV particles were measured using the bicinchoninic acid protein assay (Thermo Fisher Scientific, Catalog #23225).

Proteomic assays were performed across multiple analytical batches because of sample volume and instrument capacity constraints. To mitigate systematic bias, samples were randomized across batches using a stratified randomization strategy that balanced treatment group, responder status, sex, and study time point, thereby ensuring proportional representation of these key covariates within each batch. Quality control procedures included the inclusion of pooled reference plasma samples in every batch to monitor interbatch variability, the analysis of technical replicates, assay controls, and calibration standards per manufacturer specifications. Proteins exceeding coefficient of variation thresholds (>12%–20%) were excluded from subsequent analyses. All downstream statistical modeling was conducted using batch‐corrected, log‐transformed, and standardized protein expression values.

For proteomic quantification, Aβ42, Aβ40, and total tau (t‐tau) were assessed using the Simoa Neurology 3‐Plex E kit, while NfL and GFAP were analyzed using the Simoa Neurology 2‐Plex E kit, both using the HD‐X analyzer. The Meso Scale Discovery (MSD) platform was used for the p‐tau 181 and 231 assays. The proinflammatory proteins interferon (IFN), interleukin (IL)‐10, IL‐12p70, 1L‐13, IL‐1B, IL‐2, IL‐4, IL‐6, IL‐8, and tumor necrosis factor alpha (TNF‐ɑ) in the samples were assayed using V‐PLEX Proinflammatory Panel 1 Human Kit on the MSD platform. All samples were duplicated with a 4‐fold sample dilution and were loaded onto a 96‐well plate and analyzed along with interassay controls for both HD‐X and MSD platforms. The combined proteomic results from MSD and HD‐X assays were used to represent a broader biomarker profile (sections 3.2, 3.3, 3.4, 3.5) and reflect a broader proteomic biomarker profile. *APOE* proteotype was analyzed for this study at C2N Diagnostics’ Clinical Laboratory Improvement Amendments (CLIA)‐certified lab (Precivity‐AD) using immunoprecipitation liquid chromatography‐mass spectrometry. The outcomes of the *APOE* proteotype analysis were compared to the *APOE* genotype from the original clinical trial data.

### 2.4 Statistical analyses

To conduct the statistical analysis, R (4.4.2) was used.^50^ A support vector machine (SVM) algorithm analysis using a training‐only approach was used to construct proteomic profiles of D/ND responders versus non‐responders and to assess changes in treatment response over time due to the small sample size.^51^ SVM classifies tasks by defining hyperplanes in multidimensional space to divide cases with various class labels. We used 10 times 5‐fold cross‐validation for SVM analysis in this study.^52^ Ten times repeated 5‐fold cross‐validation divides the data into five folds, trains the model on four folds, and tests it on the remaining one, repeating this process 10 times to ensure robustness. Using the mean, evaluation metrics are summarized. It decreases the variance of a single cross‐validation trial, making out‐of‐sample performance estimates more accurate. The 10‐times repeated 5‐fold cross‐validation–based parameter optimization method was applied to evaluate blood‐based proteomic profiles and diagnostic prediction performance. Additionally, to further evaluate model robustness and mitigate overfitting, we reanalyzed the dataset using a training–testing and splitting approach for cognitive diagnosis prediction. However, the implementation of this strategy for response‐related prediction models was limited by a small sample size, resulting in insufficient power and unstable performance estimates.

Diagnostic accuracy was assessed using receiver operating characteristic (ROC) curves, and the covariates included were *APOE* carrier status, sex, and age. Statistical measures such as accuracy, sensitivity (SN), specificity (SP), negative predictive value (NPV), and positive predictive value (PPV) were computed and reported. The analysis proceeded as follows: detecting D versus ND in DS cohort using baseline biomarkers, detecting whether proteomic profiles of D/ND could predict treatment responders (yes/no) using baseline biomarkers, and detecting whether proteomic profiles of D/ND determine the change in treatment response (changed treatment responders [CR]/non‐changed treatment responders [NCR]) of anti‐inflammatory treatment and vit E over 36 months using biomarkers measured at the same interval. Responder classification was determined using a combined biomarker‐driven and clinical endpoint framework. Consistent with the previously validated NSAIDs and REFLECT precision‐medicine approach, we implemented an SVM‐based training‐only modeling strategy to classify treatment response.^43^, ^44^, ^53^ Baseline plasma protein expression levels from the biomarker panel were used to generate a predictive response signature.^43^, ^44^, ^53^ Participants whose baseline biomarker profiles matched the predefined response signature and who demonstrated stability or improvement in ARC scores over the trial duration were classified as responders, indicating a biologically predicted benefit from vit E. In contrast, individuals lacking the predictive biomarker signature and exhibiting decline on ARC during the same period were classified as non‐responders. Classification performance was assessed by the SVM training‐only model's ability to discriminate between treatment benefit and non‐benefit across the dataset, consistent with the high predictive accuracy reported in previous analyses.^43^, ^44^, ^53^

Descriptive statistics were used to summarize participant characteristics and biomarker distributions. Group differences between D/ND individuals were assessed using Wilcoxon rank‐sum tests for continuous biomarkers (e.g., age) and chi‐squared tests for categorical variables (e.g., *APOE*, sex). The AUC for individual biomarkers was estimated using ROC analysis in the pROC package in R to evaluate their ability to differentiate between D/ND individuals and responders versus non‐responders and to assess changes in treatment response over time. Linear regression was used to assess the association between ARC and protein markers.

## 3 RESULTS

In this proof‐of‐concept investigation, a total of 276 samples were analyzed across three groups: plasma, aEV, and nEV. For each group, baseline (*n* = 138) and 36‐month follow‐up (*n* = 138) data were available for inclusion in the current analyses. The full characterization of the cohort is presented in the original clinical trial report.^33^ The demographic characteristics are in Table 1. Baseline data showed 23% met dementia criteria, aligning with the high mid‐life dementia prevalence in DS. Age, sex distribution, and *APOE* genotype frequencies did not differ significantly between D/ND groups (*P* > 0.05). Although 63% of males were in the D group and ε4 carriers were present in both groups, these differences were not statistically significant, suggesting no major demographic or genetic imbalance at baseline. For our analyses, there were a total of 32 demented and 106 ND DS individuals based on clinical outcomes. Across the trial, when changes in ARC of clinical outcome scores were measured from baseline to 36 months, 71 participants were categorized as stable or improved, while 67 were categorized as decliners. We examined the SVM algorithm using two sets of predictive markers, both individually and in combination. We used proinflammatory panels (IFN, IL‐10, IL‐12p70, IL‐13, IL‐1β, IL‐2, IL‐4, IL‐6, IL‐8, and TNF‐α) for the first set of predictive markers. The second set was A/T/N biomarkers, including Aβ42, Aβ40, NfL, GFAP, total tau, and p‐tau 181 and 231. These markers individually and combined predicted the cognitive status, treatment response versus non‐response, and change in treatment response from baseline (0 months) to endpoint (36 months) based on evaluating the ARC as measured by a combination of primary and secondary endpoints (BPT, VT, and BFDS) within the Vit E trial in adults with DS.

**TABLE 1:** Demographic characteristics of the vitamin trial in the Down syndrome cohort.

Characteristics	Overall, *N* = 138	Demented, *N* = 32	Non‐demented, *N* = 106	*P* value
Age	54.20 (3.71)	55.41 (4.61)	53.84 (3.33)	0.12
Sex				0.47
F	59/138 (43%)	12/32 (38%)	48/106 (45%)	
M	78/138 (57%)	20/32 (63%)	58/106 (55%)	
*APOE*				0.16
ε2/ε2	1/138 (0.7%)	1/32 (3.1%)	0/106 (0%)	
ε2/ε3	25/138 (18%)	8/32 (25%)	17/106 (16%)	
ε2/ε4	4/138 (2.9%)	2/32 (9.4%)	2/106 (1.9%)	
ε3/ε3	84/138 (61%)	18/32 (56%)	66/106 (62%)	
ε3/ε4	22/138 (16%)	3/32 (9.4%)	19/106 (18%)	
ε4/ε4	2/138 (1.4%)	0/32 (0%)	2/106 (1.9%)	

Abbreviation: *APOE*, apolipoprotein E.

### 3.1 sEV authentication and characterization

sEV was the major type of EV in our preparations, as shown in Figure S1 in supporting information. The enriched aEV and nEV had the spherical shape as expected for sEV. Enriched aEV, nEV, and negative control (no‐sEV) labeled with FITC green‐fluorescent label (Exo‐FITC, System Biosciences Inc.) and FACS sorted showed < 0.1% FITC‐positive particles in the negative controls (Figure S1A–D). In comparison, sEV preparations from D and ND DS groups had 99.0% to 99.8% Exo‐FITC–positive particles. FITC = ‐positive events showed no significant difference in nEV and aEV recovery between the two groups, as determined by quantification. It suggested a successful enrichment of aEV and nEV from sEV preparations against neuronal and astrocyte sources. The diameter and count in aEV and nEV were measured from D and ND, showing a mean particle size for both aEV and nEV ≈ 70 nm with no significant differences between the groups. However, significant quantitative differences in particle concentration and count were observed between aEV (ND = 1.27E + 10 ± 8.51E + 09 and D = 1.74E + 10 ± 6.35E + 09) and nEV (ND = 7.97E +09 ± 2.07E + 09 and D = 9.29E + 09 ± 8.12E + 09), with a particle increase in both measures in the D compared to the ND (Figure S1E–H). aEV and nEV were finely dispersed, and EV aggregation was unlikely to be a confounder in proteomic assays (Figure S1I–L). Plasma concentrations of the EV marker CD81 showed a significant increase in D compared to the ND for both aEV (ND = 10.3 ± 2 and D = 16.1 ± 2.2) and nEV (ND = 6.9 ± 1.5 and D = 11.1 ± 2.1; Figure S1M). This suggests an upregulation of EV‐associated CD81 in individuals with DS and dementia, potentially reflecting increased EV biogenesis or altered vesicle composition associated with disease pathology.

### 3.2 Univariate AD biomarkers over time, cognition, *APOE*, and sex

Each of the protein biomarkers from neat plasma, aEV, and nEV was evaluated independently for accuracy in determining cognitive status and therapeutic efficacy in the vit E trial. The AUCs for individual biomarkers (no algorithm) over time, *APOE*, age, and sex are reported in Figure S2 in supporting information. Individual biomarker levels were correlated to cognitive assessments (ARC) to evaluate their utility in tracking D and ND‐DS individuals, treatment response (yes/no), and change in treatment response (changed/no change).

### 3.3 Prediction of cognitive diagnosis using individual panels of proinflammatory, A/T/N, and combined proinflammatory and A/T/N panels

The cognitive diagnosis training‐only algorithm using inflammatory protein markers identified 27/32 Ds and 106/106 NDs in plasma, 17/32 Ds and 90/106 NDs in aEV, and 31/32 Ds and 74/106 NDs in nEV. The SVM proinflammatory‐based plasma profile was highly accurate in predicting cognition status (D/ND), AUC = 0.97, SN = 0.84, SP = 1.0, NPV = 95%, PPV = 100% (Figure S3A in supporting information). The top plasma proinflammatory markers IL‐10, IL‐6, and IFN yielded an AUC of 0.55, 0.54, and 0.46, respectively (Figure S4A in supporting information). AUC = 0.79, SN = 0.54, SP = 0.85, NPV = 85%, and PPV = 51% were observed in the proinflammatory‐based profile of cognitive diagnosis using aEV (Figure S3B), while nEV provided AUC = 0.92, SN = 0.96, SP = 0.69, NPV = 98%, and PPV = 49% (Figure S3C). In aEV, the top proinflammatory markers IL‐4, TNF‐ɑ, and IL‐6 produced AUCs of 0.70, 0.72, and 0.67, respectively (Figure S3A). IL‐8, IL‐1B, and IL‐4 were top candidates in nEV with AUCs of 0.65, 0.68, and 0.65, respectively (Figure S4A).

With A/T/N markers, the algorithm identified 31/32 Ds and 104/106 NDs in plasma, 32/32 Ds and 85/106 NDs in aEV, and 31/32 Ds and 103/106 NDs in nEV. The SVM algorithm for A/T/N markers accurately detected plasma‐based profile D/ND among the DS population with AUC = 0.99, SN = 0.96, SP = 0.98, PPV = 97%, and NPV = 99% (Figure S5A in supporting information). For comparison purposes, the top plasma A/T/N markers Aβ40, Aβ42, and p‐tau 181 individually yielded an AUC = 0.87, 0.81, and 0.92, respectively (Figure S4A). aEV profiles accurately yielded AUC = 1.0, SN = 1.0, SP = 0.80, PPV = 60%, and NPV = 100% (Figure S5B), whereas A/T/N markers from nEV provided an AUC = 0.95, SN = 0.87, SP = 0.97, PPV = 90%, and NPV = 96% (Figure S5C). The top aEV A/T/N markers NfL, GFAP, and Aβ42/Aβ40 provided an AUC = 0.83, 0.72, and 0.77, respectively (Figure S4A), while the top A/T/N markers from nEV were tau, Aβ40, and GFAP with an AUC of 0.92, 0.86, and 0.62, respectively (Figure S4A). We also looked into individual AUCs of GFAP, NfL, and tau ratios, including p‐tau181/Aβ42, p‐tau231/Aβ42, and p‐tau231/p‐tau181 in all three groups (Figure S4A).

**FIGURE 1:**
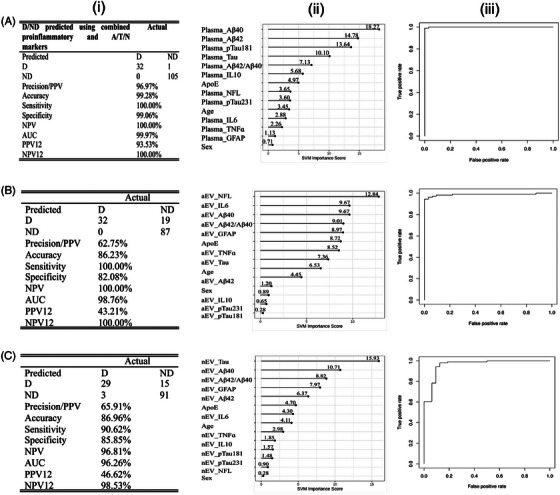
SVM analyses using a testing‐only algorithm for the combined baseline proinflammatory and A/T/N biomarkers in plasma (A), aEV (B), and nEV (C), to predict cognitive status D versus ND in adults with Down syndrome. A‐i, SVM class statistics of the area under the curve, accuracy, sensitivity, specificity, and negative predictive value in plasma; (A‐ii) SVM variable importance values plot in plasma; and (A‐iii) ROC curve assessing the accuracy of algorithm prediction in plasma. B‐i, SVM class statistics of the area under the curve, accuracy, sensitivity, specificity, and negative predictive value in aEV; (B‐ii) SVM variable importance values plot in aEV; and (B‐iii) ROC curve assessing the accuracy of algorithm prediction in aEV. C‐i, SVM class statistics of the area under the curve, accuracy, sensitivity, specificity, and negative predictive value in nEV; (C‐ii) SVM variable importance values plot in nEV; and (C‐iii) ROC curve assessing the accuracy of algorithm prediction in nEV. aEV, astrocytic extracellular vesicle; A/T/N, amyloid/tau/neurodegeneration; AUC, area under the curve; D, demented; ND, non‐demented; nEV, neuronal extracellular vesicle;; NPV, negative predictive value; PPV, positive predictive value; ROC, receiver operating characteristic; SVM, support vector machine.

Our combined approach (integrating inflammatory and neuropathological data into a single algorithm) for predicting the cognitive status showed robust and impressive performance in all three biofluid compartments with high sensitivity and accuracy: 32/32 Ds and 105/106 NDs in plasma, 32/32 Ds and 87/106 NDs in aEV, and 29/32 Ds and 91/106 NDs in nEV. The combined algorithm achieved an AUC = 0.99 with SN = 1.0, SP = 0.99, NPV = 100%, and PPV = 96% with an optimized cut‐off of 0.5 for distinguishing D/ND status using plasma samples (Figure 1A). The top three markers identified by the combined algorithm in plasma were predominantly A/T/N markers, Aβ40, Aβ42, and p‐tau 181 with an AUC = 0.87, 0.81, and 0.92, respectively (Figure S4A). The combined algorithm for aEV yielded an AUC of 0.98 with SN = 1.0, SP = 0.82, NPV = 100%, and PPV = 62%, while nEV showed AUC = 0.96, SN = 0.90, SP = 0.85, NPV = 98%, and PPV = 65% (Figure 1B,C). The combined algorithm selected NfL, IL‐6, and Aβ40 as the top three markers in aEV, with AUC values of 0.83, 0.67, and 0.77, respectively (Figure S4A). The top three markers in nEV were primarily A/T/N‐related: tau, Aβ40, and Aβ42/40 with AUCs of 0.92, 0.86, and 0.79, respectively (Figure S4A). Although all three profiles demonstrated high accuracy in distinguishing D/ND status, the covariates age, sex, and *APOE* were not among the top five most important variables in either panel analysis.

When reanalyzed using a training‐test splitting model (Figure S6 in supporting information), the combined approach's performance remained strong but showed modest reductions, consistent with reduced overfitting and more conservative generalization estimates. Plasma retained the highest robustness, with preserved high SN and SP, while nEV showed the greatest decline in PPV and overall accuracy (accuracy ≈ 99%, AUC ≈ 0.95), reflecting greater variability in nEV measures and smaller effective sample sizes. Importantly, the top predictors across compartments were predominantly A/T/N biomarkers, supporting the model's biological coherence. Demographic covariates (age, sex, *APOE*) were not among the top contributors in either modeling strategy, suggesting that biomarker‐driven classification outweighed traditional risk factors within this DS cohort. Overall, the training‐only model demonstrates proof‐of‐concept discriminatory capacity, while the training‐test split confirms substantial, though slightly attenuated, predictive performance, supporting model stability while appropriately tempering expectations for real‐world generalizability.

### 3.4 Prediction of treatment responders using baseline plasma proinflammatory, A/T/N, and combined proteomics panel

For predicting vit E response on both treatment and placebo arms, the baseline proinflammatory predictive biomarkers detected 71 responders and 67 non‐responders with plasma, aEV, and nEV, and accurately predicted treatment response in 64/71 responders and 67/67 of the non‐responders in plasma; 59/71 responders and 42/67 of the non‐responders in aEV, and 60/71 responders and 67/67 of the non‐responders in nEV. Plasma proinflammatory markers yielded an AUC = 0.99, SN = 0.90, SP = 1.0, NPV = 90%, and PPV = 100% (Figure S7A in supporting information). The top inflammatory markers to predict the responders versus non‐responders were IL‐2, TNF‐ɑ, and IFN, and for comparison purposes individually, they yielded an AUC = 0.49, 0.51, and 0.51, respectively (Figure S4B). aEV proinflammatory markers yielded an AUC = 0.78, SN = 0.83, SP = 0.62, NPV = 77%, and PPV = 70% (Figure S7B) with top markers being IFN, IL‐13, and IL‐1B. They individually yielded an AUC = 0.50, 0.57, and 0.51, respectively (Figure S4B). Proinflammatory markers in nEV provided an AUC = 1.0, SN = 0.84, SP = 1.0, NPV = 85%, and PPV = 100% (Figure S7C). The top predictive markers observed in nEV were IL‐12p70, TNF‐ɑ, and IL‐13 with individual AUC = 0.56, 0.57, and 0.58, respectively (Figure S4B).

The SVM algorithm for A/T/N markers also detected a total of 71 responders and 67 non‐responders and predicted 45/71 responders and 53/67 of the non‐responders in plasma; 71/71 responders and 44/67 of the non‐responders in aEV, and 53 of the 71 responders and 67/67 of the non‐responders in nEV. The plasma A/T/N markers provided an AUC = 0.73, SN = 0.63, SP = 0.79, NPV = 67%, and PPV = 76% (Figure S8A in supporting information). The top neuropathological markers that predicted the responders from non‐responders were GFAP, Aβ42, and NfL, with AUC = 0.53, 0.52, and 0.50, respectively (Figure S4B). The A/T/N markers in aEV showed AUC = 0.93, SN = 1.0, SP = 0.65, NPV = 100%, and PPV = 75% with the top three markers enlisted as p‐tau181, Aβ42, and NfL with individual AUC = 0.52, 0.52, and 0.57, respectively (Figures S8B and S4B) whereas the A/T/N markers from nEV yielded AUC = 0.97, SN = 0.74, SP = 1.0, NPV = 96%, and PPV = 100% with the top three markers as p‐tau231, GFAP, and Aβ42/Aβ40 with individual AUC = 0.53, 0.54, and 0.59, respectively (Figures S8C and S4B). We also looked into individual AUCs of GFAP, NfL, and tau ratios, including p‐tau181/Aβ42, p‐tau231/Aβ42, and p‐tau231/p‐tau181 for all three panels (Figure S4B).

**FIGURE 2:**
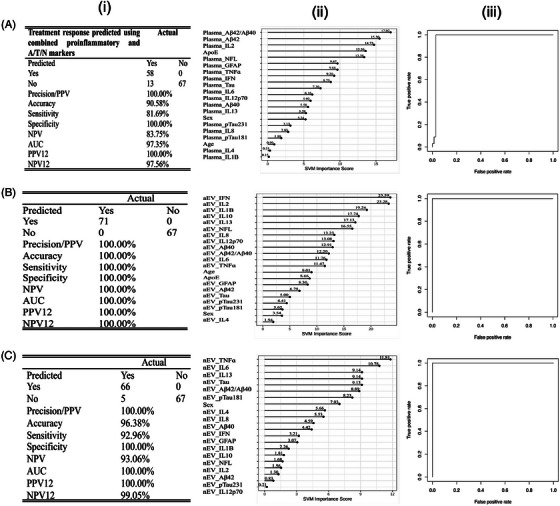
Predictive accuracy of combined proinflammatory and A/T/N biomarkers at baseline for identifying responders versus non‐responders (yes/no) using SVM testing‐only algorithm. The analyses were conducted in plasma (A), aEV (B), and nEV (C) from adults with Down syndrome. A‐i, SVM class statistics of the area under the curve, accuracy, sensitivity, specificity, and negative predictive value in plasma; (A‐ii) SVM variable importance values plot in plasma; and (A‐iii) ROC curve assessing the accuracy of algorithm prediction in plasma. B‐i, SVM class statistics of the area under the curve, accuracy, sensitivity, specificity, and negative predictive value in aEV; (B‐ii) SVM variable importance values plot in aEV; and (B‐iii) ROC curve assessing the accuracy of algorithm prediction in aEV. C‐i, SVM class statistics of the area under the curve, accuracy, sensitivity, specificity, and negative predictive value in nEV; (C‐ii) SVM variable importance values plot in nEV; and (C‐iii) ROC curve assessing the accuracy of algorithm prediction in nEV. aEV, astrocytic extracellular vesicle; A/T/N, amyloid/tau/neurodegeneration; AUC, area under the curve; D, demented; ND, non‐demented; nEV, neuronal extracellular vesicle;; NPV, negative predictive value; PPV, positive predictive value; ROC, receiver operating characteristic; SVM, support vector machine.

The combined proinflammatory and neuropathological markers for all panels again detected 71 responders and 67 non‐responders with high accuracy and predicted 58/71 responders and 67/67 of the non‐responders in plasma; 71/71 responders and 67/67 of the non‐responders in aEV, and 66/71 responders and 67/67 of the non‐responders in nEV. The plasma detected AUC = 0.97, SN = 0.81, SP = 1.0, NPV = 83%, and PPV = 100% with an optimized cutoff of 0.5 for distinguishing responder status (Figure 2A). The markers that predominated the detection of responders versus non‐responders using a combined algorithm for plasma were the Aβ42/40, Aβ42, and IL‐2, with individual AUC = 0.54, 0.52, and 0.49, respectively (Figure S4B). The aEV distinguished responder status with an AUC = 1.0, SN = 1.0, SP = 1.0, NPV = 100%, and PPV = 100% with the top three markers as IFN, IL‐2, and IL‐1B with individual AUCs 0.50, 0.54, and 0.51, respectively (Figures 2B and S4B) whereas nEV showed AUC = 1.0, SN = 0.92, SP = 1.0, NPV = 99%, and PPV = 100% with the top three markers as TNF‐ɑ, IL‐6, and IL‐13 (Figure 2C). The observed individual AUCs for these markers was 0.57, 0.50, and 0.58, respectively (Figure S4B). Although the panels were highly accurate in distinguishing responders from non‐responders, the covariates age and sex did not emerge as important variables in either panel analysis.

### 3.5 Prediction of change in treatment response (treatment) from baseline (0 months) to the end time point of trial (36 months) using plasma proinflammatory panels, A/T/N, and combined proteomic panel

Predictive proinflammatory and A/T/N biomarkers, evaluated both individually and through a combined SVM algorithm, were used in parallel to ARC based on a composite of primary and secondary endpoints (BPT, VT, and BFDS) from baseline to 36 months. A total of 72 participants were classified as exhibiting a stable treatment response, changed responder (treatment; i.e., a measurable change in treatment trajectory, CR), whereas 66 were classified as unstable, non‐changed responder (i.e., no significant change in treatment, NCR). In plasma, the proinflammatory biomarkers predicted treatment in 24/72 CR and all 66 NCR, in aEV 69/72 CR and all 66 NCR, and in nEV 72/72 CR and only 16/66 NCR. The plasma predictive inflammatory markers algorithm with an optimal SVM‐based cutoff score of 0.5 provided an AUC = 0.92, SN = 0.33, SP = 1.0, NPV = 95%, and PPV = 100%. The top predictive markers identified were TNF‐ɑ, IL‐8, and IFN (Figure S9A in supporting information). The aEV predictive inflammatory markers provided an AUC = 1.0, SN = 0.95, SP = 1.0, NPV = 95%, and PPV = 100%, and the key markers were identified as IFN, IL‐1B, and IL‐10 (Figure S9B), while nEV provided AUC = 0.90, SN = 1.0, SP = 0.24, NPV = 100%, and PPV = 59%. The top three markers for the nEV panel were IFN, IL‐4, and age (Figure S9C). The individual AUC for top markers is shown in Figure S4C.

A/T/N biomarkers displayed different performance profiles in plasma; 55/72 CR and 63/66 NCR were predicted. In aEV, they predicted 63/72 CR and 43/66 NCR. In nEV, the algorithm predicted 54/72 CR and 38/66 NCR. The predictive A/T/N algorithm for plasma provided AUC = 0.96, SN = 0.76, SP = 0.95, NPV = 78%, and PPV = 94% with top markers as Aβ42/Aβ40 ratio, sex, and age (Figure S10A in supporting information) while for aEV we observed an AUC = 0.89, SN = 0.87, SP = 0.65, NPV = 82%, and PPV = 73% with key markers such as NfL, tau, and GFAP (Figure S10B). The nEV panel yielded an AUC = 0.72, SN = 0.75, SP = 0.57, NPV = 67%, and PPV = 65%. The top markers for this panel were NfL, GFAP, and *APOE* (Figure S10C). The observed individual AUC of top markers and GFAP, NfL, and tau ratios, including p‐tau181/Aβ42, p‐tau231/Aβ42, and p‐tau231/p‐tau181 for comparison, is shown in Figure S4C.

**FIGURE 3:**
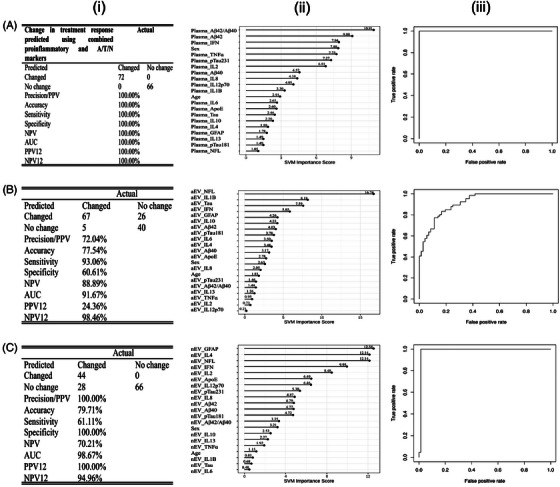
Predictive accuracy of combined proinflammatory and A/T/N biomarkers in identifying changes in treatment response versus no change from baseline (0) to 36 months using SVM testing‐only algorithm. Panels represent analyses in plasma (A), aEV (B), and nEV (C). A‐i, SVM class statistics of the area under the curve, accuracy, sensitivity, specificity, and negative predictive value in plasma; (A‐ii) SVM variable importance values plot in plasma; and (A‐iii) ROC curve assessing the accuracy of algorithm prediction in plasma. B‐i, SVM class statistics of the area under the curve, accuracy, sensitivity, specificity, and negative predictive value in aEV; (B‐ii) SVM variable importance values plot in aEV; and (B‐iii) ROC curve assessing the accuracy of algorithm prediction in aEV. C‐i, SVM class statistics of the area under the curve, accuracy, sensitivity, specificity, and negative predictive value in nEV; (C‐ii) SVM variable importance values plot in nEV; and (C‐iii) ROC curve assessing the accuracy of algorithm prediction in nEV. aEV, astrocytic extracellular vesicle; A/T/N, amyloid/tau/neurodegeneration; AUC, area under the curve; D, demented; ND, non‐demented; nEV, neuronal extracellular vesicle;; NPV, negative predictive value; PPV, positive predictive value; ROC, receiver operating characteristic; SVM, support vector machine.

The combined proinflammatory and neuropathological markers for all panels again detected 72 stable and 66 unstable with high accuracy. The predictive power improved; in plasma, all 72 CR and all 66 NCR were correctly identified, achieving perfect sensitivity (100%) and accuracy (100%). In aEV, the model predicted 67/71 CR and 40/66 NCR and in nEV, 44/72 CR and all 66 NCR. Plasma‐combined biomarkers yielded AUC = 1.0, SN = 1.0, SP = 1.0, NPV = 100%, and PPV = 100% (Figure 3A). The markers Aβ42/40, Aβ42, and IFN predominated in distinguishing treatment in this population with individual AUC values of 0.54, 0.55, and 0.54, respectively (Figure S4C). The combined biomarkers SVM for aEV showed an AUC = 0.89, SN = 0.87, SP = 0.65, NPV = 82%, and PPV = 73%, and the top identified markers were NfL, tau, and GFAP (Figures 3B and S4c) while nEV distinguished the treatment with an AUC = 0.98, SN = 0.61, SP = 1.0, NPV = 70%, and PPV = 100%, and the top markers were GFAP, IL‐4, and NfL (Figures 3C and S4C).

The association's top three biomarkers with ARC and their concentrations in the respective biofluids for predicting cognitive diagnosis, treatment response, and treatment response change are shown in Figures S11 and S12 in supporting information, respectively.

## 4 DISCUSSION

We present a proof‐of‐concept study for the potential of a precision medicine approach to identify DS individuals who will likely benefit from anti‐inflammatory therapy. Our dual‐marker strategy leveraging plasma‐based proinflammatory and A/T/N biomarkers enabled both the prediction of dementia and the identification of treatment responders, marking a significant step toward personalized care in DS. Notably, this study is the first to simultaneously analyze proinflammatory and A/T/N biomarkers in plasma, aEV, and nEV. Without a sensitive, accessible biomarker for staging, treatment response, and patient selection, most AD clinical trials exclude DS, limiting DSAD research and exacerbating health disparities.^21^, ^54^, ^55^, ^56^, ^57^, ^58^, ^59^, ^60^ AD biomarker research has rapidly expanded, mainly in imaging and CSF, but blood biomarkers offer a promising tool for diagnosing DSAD, which mirrors late‐onset AD.^61^, ^62^, ^63^, ^64^, ^65^, ^66^, ^67^, ^68^, ^69^, ^70^ Our team previously demonstrated that blood‐based inflammatory and A/T/N markers can predict treatment response and cognitive decline using samples from the AD anti‐inflammatory trial (ADAPT) and the Health and Aging Brain Study‐Health Disparities (HABS‐HD study), respectively.^41^, ^53^ We used a broader proinflammatory panel since these indicators are well‐studied and linked to neuroinflammation in AD and DS.^71^, ^72^, ^73^, ^74^, ^75^, ^76^, ^77^, ^78^, ^79^ In a vit E trial, our biomarker panels effectively predicted dementia and treatment responders and changes, with several participants showing reduced inflammatory markers and stabilized ARC scores.

Using baseline proinflammatory and A/T/N biomarkers, the combined SVM algorithm accurately predicted DS treatment responders, achieving 100% PPV across all modalities and nearly 100% NPV in EV. The prominence and predictive value of cytokines such as IFN, IL‐1β, IL‐6, IL‐2, TNF‐α, and IL‐13 underscore a strong immunological basis for treatment response in the DS population, highlighting inflammation as a key therapeutic driver. Further, it also marks a key advance in identifying likely treatment responders, essential for optimizing clinical trial design and therapeutic targeting in DS. It highlights the power of cell type–specific liquid biomarkers for guiding therapeutic decisions, as aEV and nEV outperform plasma in predictive power, suggesting that cell type–specific biomarker cargo enhances diagnostic precision and may amplify the signal of brain‐specific changes that may be diluted in peripheral blood. These findings support the use of baseline EV‐based biomarkers to stratify patients with DS who are most likely to respond to therapeutic interventions, thereby reducing exposure to ineffective treatments and optimizing resource allocation.

DS individuals have chronic immune dysregulation due to triplication of IFN receptor genes on chromosome 21, which increases IFN signaling and inflammation and accelerates AD pathology (e.g., type I IFN hypersensitivity and immune remodeling in DS).^80^ Elevated IL‐6 and TNF‐α in DSAD suggest a role for neuroinflammation in cognitive decline, while IL‐2 and IL‐13 indicate broader immune changes.^57^, ^58^, ^81^, ^82^ Some predictors have modest individual discrimination, but combined in models, they reveal integrated “immuno‐oxidative” profiles relevant to DSAD vulnerability. Vit E's antioxidant and anti‐inflammatory properties modulate nuclear factor kappa beta cytokine production and reduce OS, supporting indirect effects but not directly regulating all cytokines. Thus, the predictive values of identified cytokines should be interpreted within this broader systems framework.

Comparing how baseline biomarkers performed in predicting treatment over 36 months revealed that proinflammatory biomarkers excelled in aEV, where they primarily captured inflammation‐related processes, but performed poorly in plasma; maybe systemic noise obscured relevant signals. On the other hand, A/T/N biomarkers showed a more balanced performance across biofluids, with reasonable predictive power in plasma and aEV but limited efficacy in nEV, where their specificity to neuronal processes may have restricted their ability to detect broader treatment effects. The plasma‐based algorithm demonstrated perfect predictive accuracy (100% SN, SP, NPV, and PPV), ensuring all true CRs are identified while accurately excluding NCRs to prevent unnecessary treatment and optimize health‐care resources. In contrast, the aEV model showed high sensitivity (87%) but moderate specificity (65%), with a PPV of 73% and NPV of 82%, making it well suited for clinical trial screening or enrichment strategies focused on maximizing true CR inclusion despite lower reliability in ruling out NCR. The nEV model, with 100% specificity and PPV but lower sensitivity (61%) and moderate NPV (70%), is best suited for confirming NCR and excluding unlikely responders, especially when treatment risks or resource constraints are high.

The SVM algorithm for predictive proinflammatory and A/T/N markers predicting dementia performed best in plasma. Proinflammatory markers showed high sensitivity, accuracy, and near‐perfect predictive values (NPV = 96%–100%, PPV = 96%–100%) for identifying D in DS. In contrast, aEV was limited by lower sensitivity and reduced ability to detect all D, despite a still‐decent overall accuracy (77%). In nEV, the algorithm showed strong sensitivity and NPV but lower PPV, suggesting it is more helpful in ruling out rather than confirming cognitive decline. For A/T/N markers, the plasma‐based algorithm again excelled, delivering nearly ideal NPV and PPV. While aEV showed perfect sensitivity and NPV, their moderate PPV (60%) limits diagnostic certainty. However, nEV showed high predictivity in both directions (NPV = 96%, PPV = 90%), underscoring its potential for early identification as well as exclusion of cognitive decline. However, the combined algorithm performs impressively across all biofluids, but plasma remains the most reliable for accurate prediction of D. The combined approach in plasma yielded near‐perfect diagnostic accuracy (NPV = 100%, PPV = 96%), highlighting its potential as the most powerful and reliable biomarker combination for either identifying or excluding D in DS while in EV, the combined algorithm maintained a high NPV; however, their PPV remained modest, with 100% sensitivity in aEV but a slight decrease (90%) in nEV, likely reflecting greater heterogeneity within the aEV and nEV populations, potentially leading to an inaccurate distinction between D and ND. Each biofluid type offers unique advantages, but the combined algorithm performs well across all compartments. Plasma remains the most robust and reliable medium, with near‐perfect diagnostic accuracy. EV‐based data add value in sensitivity and may be most effective when paired with plasma data or used in longitudinal tracking, in which early, subtle changes matter.

Comparing the training‐only and training‐test splitting analyses provided important context for interpreting model performance and supports the rationale for our primary training‐only approach in this study. The splitting model showed near‐perfect discrimination, especially in plasma (AUC = 0.99, SN = 1.0, SP = 0.99), indicating strong separation between D and ND. With a splitting model, performance remained robust, though accuracy and PPV showed a modest decline, especially in the nEV compartment. This pattern is consistent with reduced statistical power and increased variance inherent in smaller effective test samples rather than model instability. Importantly, the relative preservation of high AUC and SN across validation strategies indicates that the signal captured by the combined inflammatory and A/T/N biomarker algorithm is not solely an artifact of overfitting. The persistence of A/T/N as top contributors across modeling approaches further supports the biological coherence and reproducibility of feature importance. Given the modest size of the D subgroup, a conventional split substantially reduces the sample size per partition and may yield unstable estimates; thus, the training‐only framework, supplemented with cross‐validation, permutation testing, and balanced accuracy metrics, is a statistically appropriate strategy for these data constraints. Together, the two model results confirm that the training‐only model detects a strong, biologically relevant classification signal and rightly presents its performance as a proof‐of‐concept awaiting validation in larger, independent cohorts.

Here, we showed that targeting inflammation markers of AD progression in DS can accurately predict cognitive status and treatment response in the DSAD, aligning with the broader literature on the role of neuroinflammation in DSAD pathology.^71^, ^72^, ^73^, ^74^, ^75^, ^76^, ^77^, ^78^, ^79^ We also provide direct evidence that a blood‐based A/T/N profile can detect underlying neurodegeneration and predict therapeutic responders versus non‐responders in the vitamin E trial, as assessed by ARC of primary and secondary outcomes in the DS. The overall profile was highly significant across biomarker classifications, and plasma levels of Aβ42, Aβ40, Aβ42/40, p‐tau181, and p‐tau231 were robust predictors of cognitive impairment in DS. Amyloid peptides and their ratio, and NFL, drove the predictive treatment response profiles, often falling within the top five markers on the variable importance plots. These biomarkers align with established A/T/N criteria for AD diagnosis in the general population, supporting their applicability in DS.^9^, ^11^, ^16^ Further, the differential performance of three biofluid panels suggests that no single biofluid or biomarker type is sufficient for comprehensive treatment monitoring and that a combination of biofluids and biomarker panels might be necessary to capture the full spectrum of biological processes underlying cognitive decline and treatment response.

We also examined how sex, age, and *APOE* influence cognitive decline and treatment outcomes. Although sex and age showed no significant effect, *APOE* consistently influenced treatment response, making it one of the top predictors.^83^, ^84^, ^85^, ^86^, ^87^, ^88^, ^89^, ^90^ Previous research indicates that sex acts more as a secondary modifier than a primary risk factor.^18^, ^21^ Additionally, age is the most significant risk factor for AD in DS, with a sharp increase after age 50; neuropathological changes are almost universal by age 40. However, the absence of a significant age difference in our cohort suggests that other factors, such as genetic background, neuroinflammation, OS, or metabolic vulnerabilities, likely modulate the timing of clinical onset beyond chronological age.^18^, ^21^ We did not detect an effect of *APOE* on the algorithm's prediction of D, likely because only two individuals in the DS cohort carried the *APOE* (ε4/ε4) genotype.

Despite the promising results, several limitations warrant consideration. The small D group limited statistical generalizability and increased the risk of overfitting. So, in our study, we used training‐only modeling because a standard train–test split would have yielded an underpowered, unstable test set for predicting responders versus non‐responders. Some models achieved perfect classification, but these findings are exploratory and hypothesis generating under constrained sample conditions and require validation in larger independent cohorts. Second, it is a retrospective study because we used the SVM algorithm from a previously conducted clinical trial. And despite being a randomized, double‐blind, placebo‐controlled, parallel‐group study, the sample size was relatively small, and larger multicenter trials are needed to further validate our findings. Third, a key limitation of the present study is the absence of paired CSF and neuroimaging data, which restricted our ability to directly relate plasma and EV‐derived biomarkers to established central amyloid and tau measures. Our algorithms rely solely on the ARC of combined primary and secondary outcomes of the trial, so CSF/imaging validation analyses would significantly strengthen our conclusions. Last, we observed differential performance of the SVM algorithm across three biofluid panels, raising uncertainty about whether these proinflammatory and A/T/N protein indicators are exclusively present in plasma or also circulate within EV. Future research should concentrate on a comprehensive, comparative study of isolated nEV, aEV, EV‐depleted, and neat plasma samples, which could yield essential insights into the predominant association of these protein markers with EV cargo or their free circulation in plasma and improve understanding of the cellular compartments involved in AD.

To confirm that anti‐inflammatories are effective for people with DS, a prospective trial recruiting new patients using predictive biomarkers is needed. And to address all the limitations, we propose (1) plasma–EV–CSF triangulation studies to establish cross‐compartment concordance and mechanistic linkage, (2) longitudinal EV cargo profiling extending beyond 36 months to define temporal dynamics of disease progression and treatment response, and (3) integration with amyloid and tau PET biomarkers to validate central target engagement and enhance translational relevance. Future research should also examine the longitudinal dynamics of these biomarkers and their interactions with genetic and environmental factors that influence AD progression in DS. We further suggest characterizing the proinflammatory endophenotypes and A/T/N neuropathological markers in DS cohorts like Alzheimer Biomarkers Consortium–Down Syndrome, Trial Ready Cohort Down Syndrome, and Alzheimer's Clinical Trials Consortium–Down Syndrome, as our findings indicate that these markers could be useful as predictive biomarkers for more precise applications in clinical trials.

In conclusion, our study demonstrates the feasibility and clinical utility of a precision medicine approach using plasma proinflammatory and A/T/N biomarkers to detect dementia and monitor treatment response in DSAD. These findings suggest that customized therapeutic strategies in ongoing and upcoming clinical trials for AD in DS can be adjusted based on biomarker feedback to stratify those most likely to respond to therapeutic interventions, creating a personalized treatment approach for DSAD through early identification of those at risk for rapid cognitive decline, timely initiation of treatment to alter disease trajectory, and real‐time monitoring to optimize efficacy.

## AUTHOR CONTRIBUTIONS

Robert A. Rissman, Michael S. Rafii, and Sid E. O'Bryant conceived the idea. Sonal Sukreet and Elise K. Kim conducted the experiments. Sonal Sukreet and Robert A. Rissman designed and drafted the paper. Sonal Sukreet, Robert A. Rissman, Michael S. Rafii, and Sid E. O'Bryant edited the paper. Sonal Sukreet, Melissa Petersen, and Fan Zhang conducted the data analyses. Robert A. Rissman had primary responsibility for the final content. All authors read and approved the final manuscript.

## CONFLICT OF INTEREST STATEMENT

The authors declare no conflicts of interest. Author disclosures are available in the supporting information.

## CONSENT STATEMENT

Consent was not necessary for this study. For the original trial, all clinical experiments followed IRB‐approved protocols, and patients or their informants provided written informed consent.

## Supporting information



Supporting Information

Supporting Information
