# Supplementary material for: Precision medicine for Alzheimer's disease in Down syndrome
Source: Alzheimers Dement. 2026 Apr 19;22(4):e71398. doi: 10.1002/alz.71398 (PMC13092428; doi:10.1002/alz.71398)

Supplemental Data: Precision Medicine for Alzheimer's Disease in Down Syndrome

Supplementary Figure 1: **Characterization of astrocyte- and neuron-derived extracellular vesicles (aEV and nEV) from plasma of Down Syndrome (DS) individuals. (a-d)** Flow cytometry analysis using fluorescence-conjugated antibodies shows labeling of (**a**) a non-EV negative control, (**b**) aEV labeled with GLAST (astrocyte marker), (**c**) nEV labeled with L1CAM (neuronal marker), and (**d**) quantification of GLAST+ aEV and L1CAM+ nEV normalized to total EV input from plasma of demented [D] and non-demented [ND] DS individuals. **(e-h)** Nanoparticle Flow Cytometry tracking analysis (NanoFCM) of (**e**) aEV and (**g**) nEV showing particle size distribution, concentration, and summary statistics including particle number, size range, and flow rate, quantification of NanoFCM sorted aEV and nEV indicating (**f**) a typical size range of 65-70 nm and comparable concentration profiles and (**h**) number of aEV and nEV particles resuspended in per mL 1X PBS. **(i-j)** Transmission electron microscopy (TEM) images of aEV **(i)** and nEV **(j)** showing vesicle morphology and homogeneity of sample preparation (yellow arrows); scale bar = 100 nm. **(k-l)** Scanning electron microscopy (SEM) images of aEV **(k)** and nEV **(l)** confirm spherical morphology and nanoscale size; scale bar = 1 µm. (**m**) Plasma concentrations of the EV marker CD81, as measured by human-specific ELISA. A significant increase was observed in the D group compared to the ND group of DS individuals in both aEV and nEV, suggesting elevated circulating EV levels associated with D. Data are represented as mean ± SD.

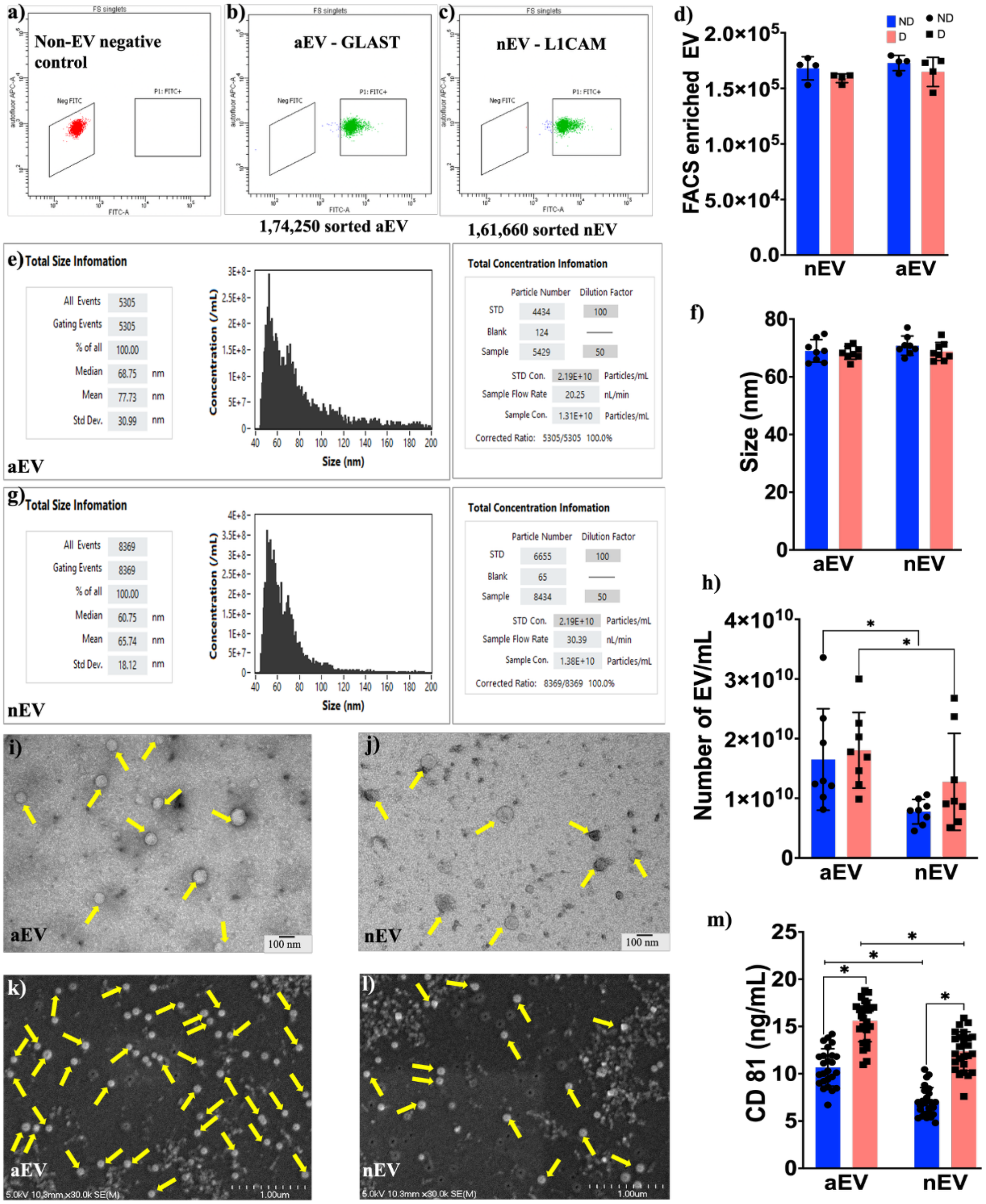

Supplemental Data: Precision Medicine for Alzheimer's Disease in Down Syndrome

Supplementary Figure 2: **Cognitive correlates of individual ATN biomarkers in DS individuals in plasma, aEV and nEV.** Area under the curve (AUC) values for individual plasma biomarkers with "***no algorithm***" over time (0 and 36 months) (**a**), ApoE genotype (no/yes) (**b**), age (≤53 years or >53) (**c**), and sex (F/M) (**d**). Biomarker levels were correlated with cognitive performance, as measured by the annual rate of change (ARC), to assess their utility for tracking cognitive status across the D and ND groups, treatment responders (yes/no), and changes in treatment response over time (change/no change).

a) <u>AUC values for individual plasma biomarkers with *no algorithm* over time (0 and 36 months)</u>

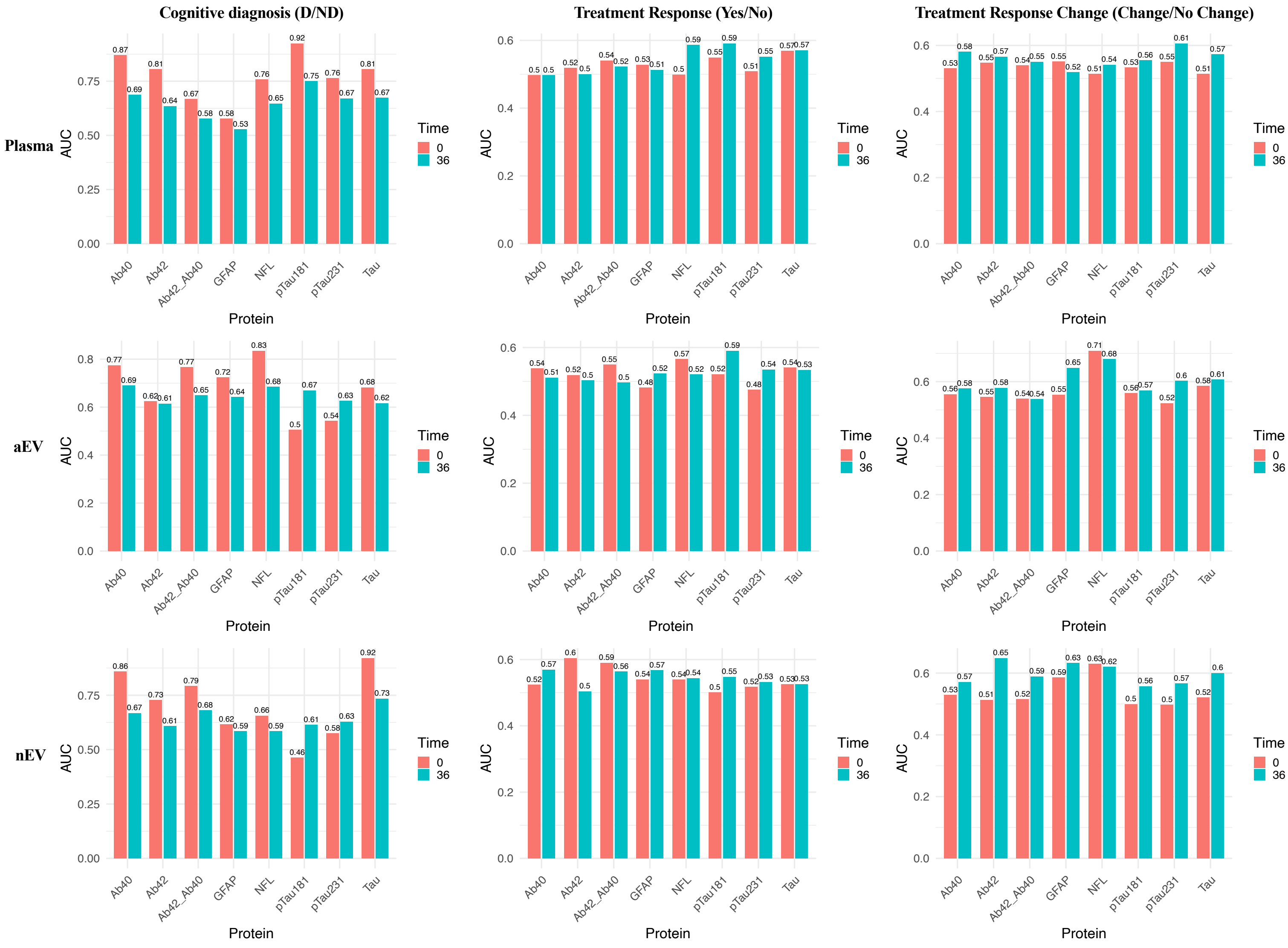

Supplemental Data: Precision Medicine for Alzheimer's Disease in Down Syndrome

b) <u>AUC values for individual plasma biomarkers with *no algorithm* over ApoE genotype (no/yes)</u>

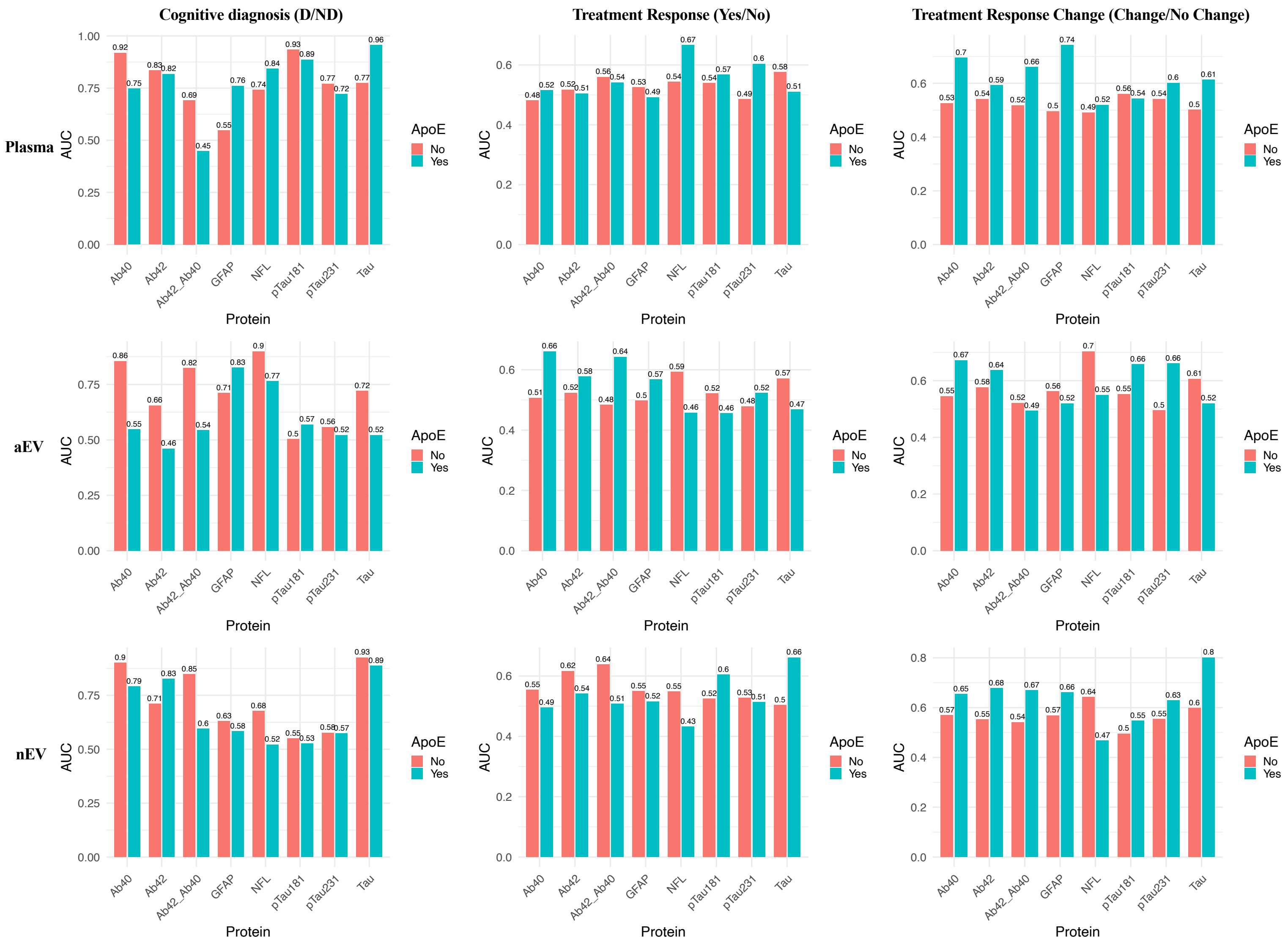

Supplemental Data: Precision Medicine for Alzheimer's Disease in Down Syndrome

c) <u>AUC values for individual plasma biomarkers with *no algorithm* over age (≤53 years or >53)</u>

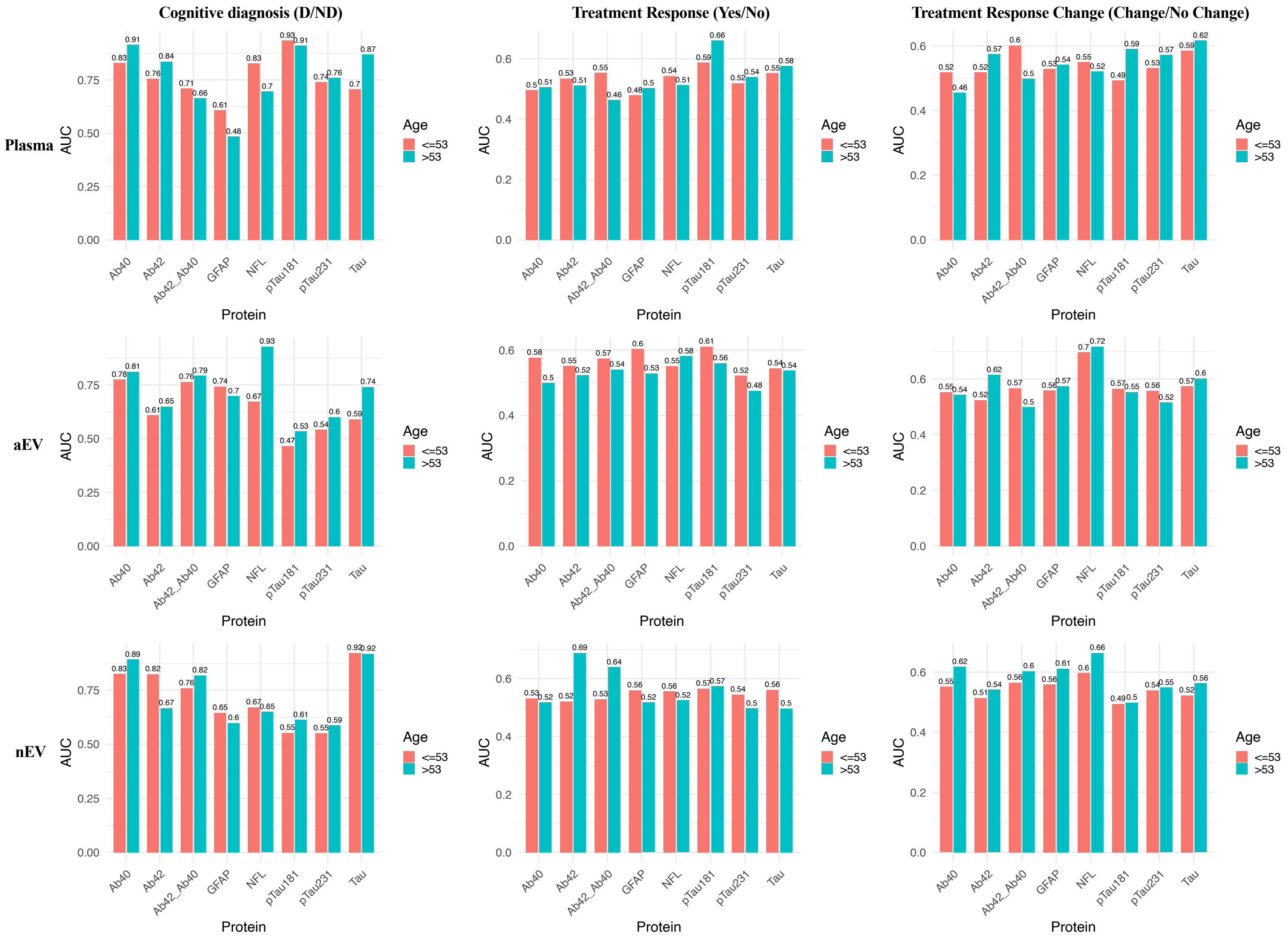

Supplemental Data: Precision Medicine for Alzheimer's Disease in Down Syndrome

d) <u>AUC values for individual plasma biomarkers with *no algorithm* over sex (F/M)</u>

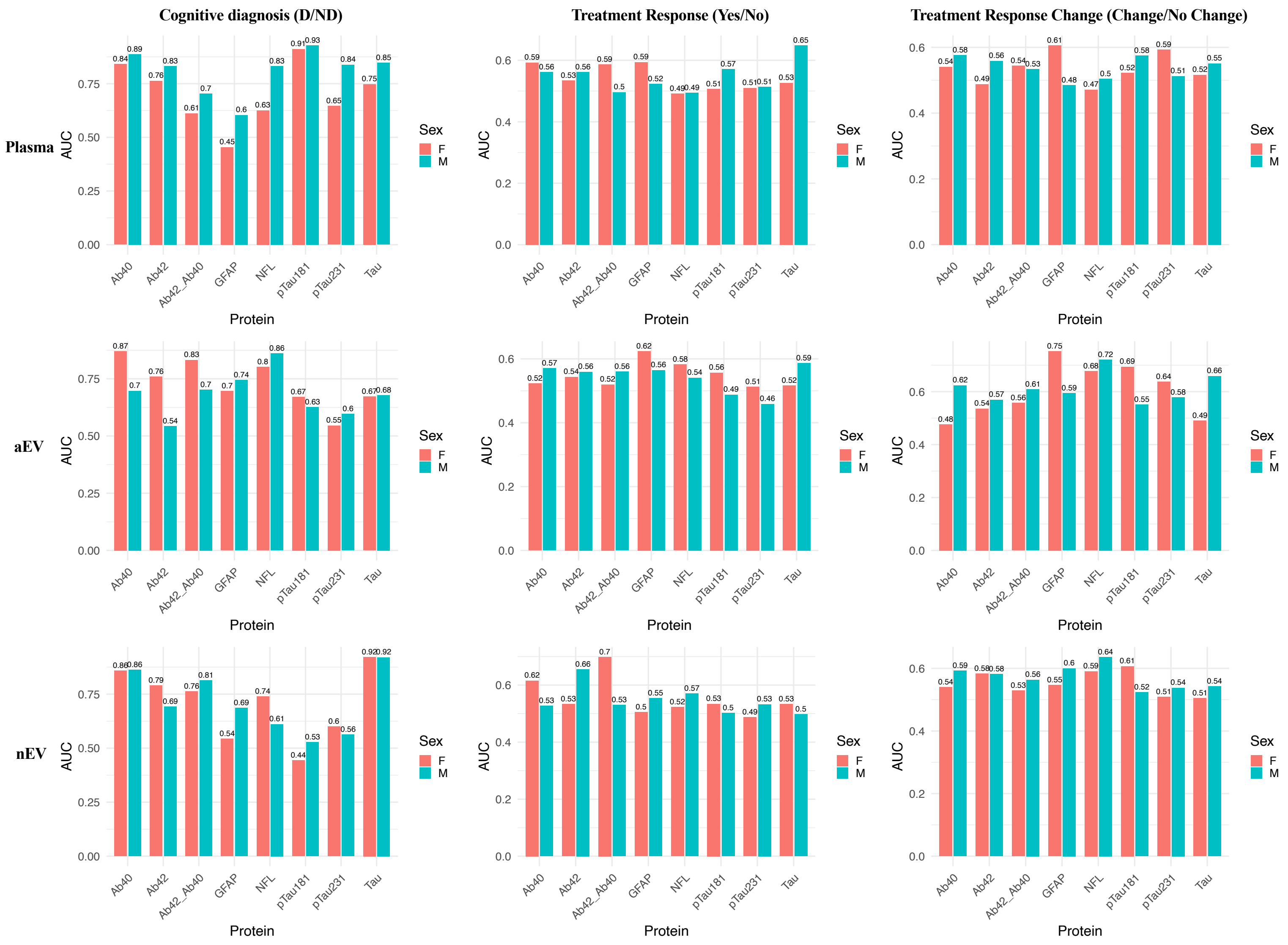

Supplemental Data: Precision Medicine for Alzheimer's Disease in Down Syndrome

Supplementary Figure 3: **Support vector machine (SVM) analyses were conducted as a training-only algorithm for baseline proinflammatory biomarkers in plasma (a), aEV (b), and nEV (c) to predict cognitive status (D vs. ND) in DS adults.** (**a-i**) SVM class statistics of the area under the curve, accuracy, sensitivity, specificity, and negative predictive value in plasma; (**a-ii**) SVM variable importance values plot in plasma; and (**a-iii**) ROC curve assessing the accuracy of algorithm prediction in plasma. (**b-i**) SVM class statistics of the area under the curve, accuracy, sensitivity, specificity, and negative predictive value in aEV; (**b-ii**) SVM variable importance values plot in aEV; and (**b-iii**) ROC curve assessing the accuracy of algorithm prediction in aEV. (**c-i**) SVM class statistics of the area under the curve, accuracy, sensitivity, specificity, and negative predictive value in nEV; (**c-ii**) SVM variable importance values plot in nEV; and (**c-iii**) ROC curve assessing the accuracy of algorithm prediction in nEV.

(i) (ii) (iii)

(a)

**D/ND predicted using proinflammatory markers**	**Actual**	
Predicted	D	ND
D	27	0
ND	5	106
Precision/PPV	100.00%	
Accuracy	96.38%	
Sensitivity	84.38%	
Specificity	100.00%	
NPV	95.50%	
AUC	97.35%	
PPV12	100.00%	
NPV12	97.91%	

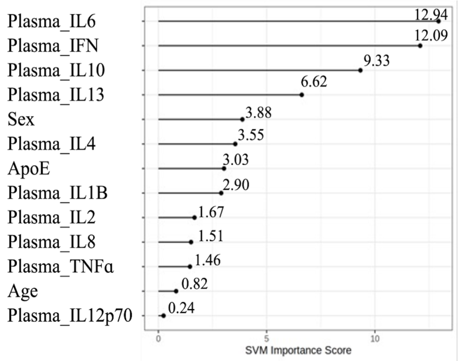

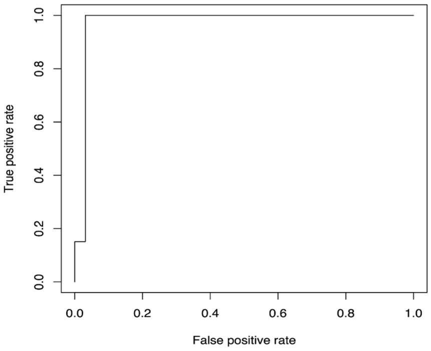

(b)

	Actual	
Predicted	D	ND
D	17	16
ND	15	90
Precision/PPV	51.52%	
Accuracy	77.54%	
Sensitivity	53.12%	
Specificity	84.91%	
NPV	85.71%	
AUC	78.89%	
PPV12	32.43%	
NPV12	93.00%	

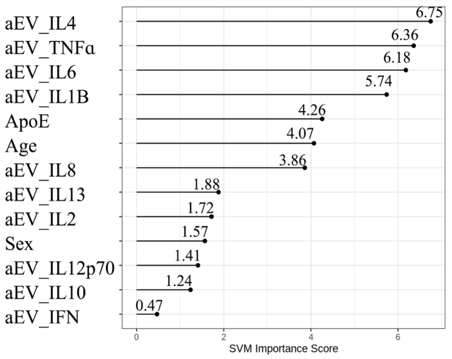

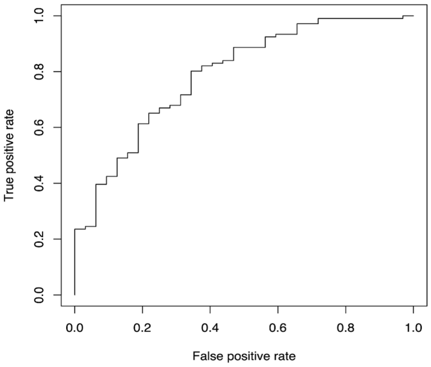

(c)

	Actual	
Predicted	D	ND
D	31	32
ND	1	74
Precision/PPV	49.21%	
Accuracy	76.09%	
Sensitivity	96.88%	
Specificity	69.81%	
NPV	98.67%	
AUC	92.45%	
PPV12	30.44%	
NPV12	99.39%	

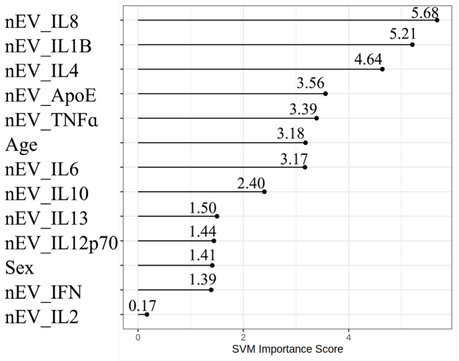

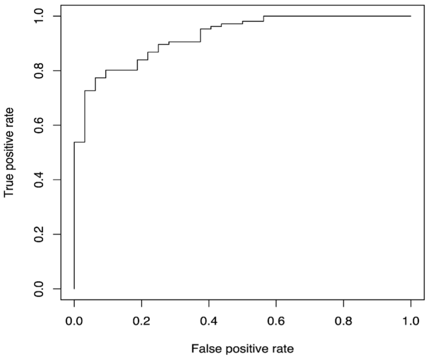

Supplementary Figure 4: Area under the curve (AUC) values for the top biomarker predictors of cognitive status and treatment efficacy in DS individuals evaluated using an SVM training-only model. Blue dots represent the AUCs for individual top biomarkers assessed across the biofluid compartments of plasma, aEV, and nEV. The vertical red line in each panel indicates the AUC of the multivariate predictive training-only model incorporating the top biomarkers identified for each outcome. (**a**) AUC distributions for the top biomarkers predicting cognitive status (demented [D] vs. non-demented [ND]) in plasma, aEV, and nEV. (**b**) AUCs for biomarkers predicting treatment responders (yes vs. no) in plasma, aEV, and nEV. (**c**) AUCs for biomarkers predicting changes in treatment response over time (change vs. no change) in plasma, aEV, and nEV.

a) <u>AUC distributions for the top biomarkers predicting cognitive status (demented [D] vs. non-demented [ND]) in plasma, aEV, and nEV</u>

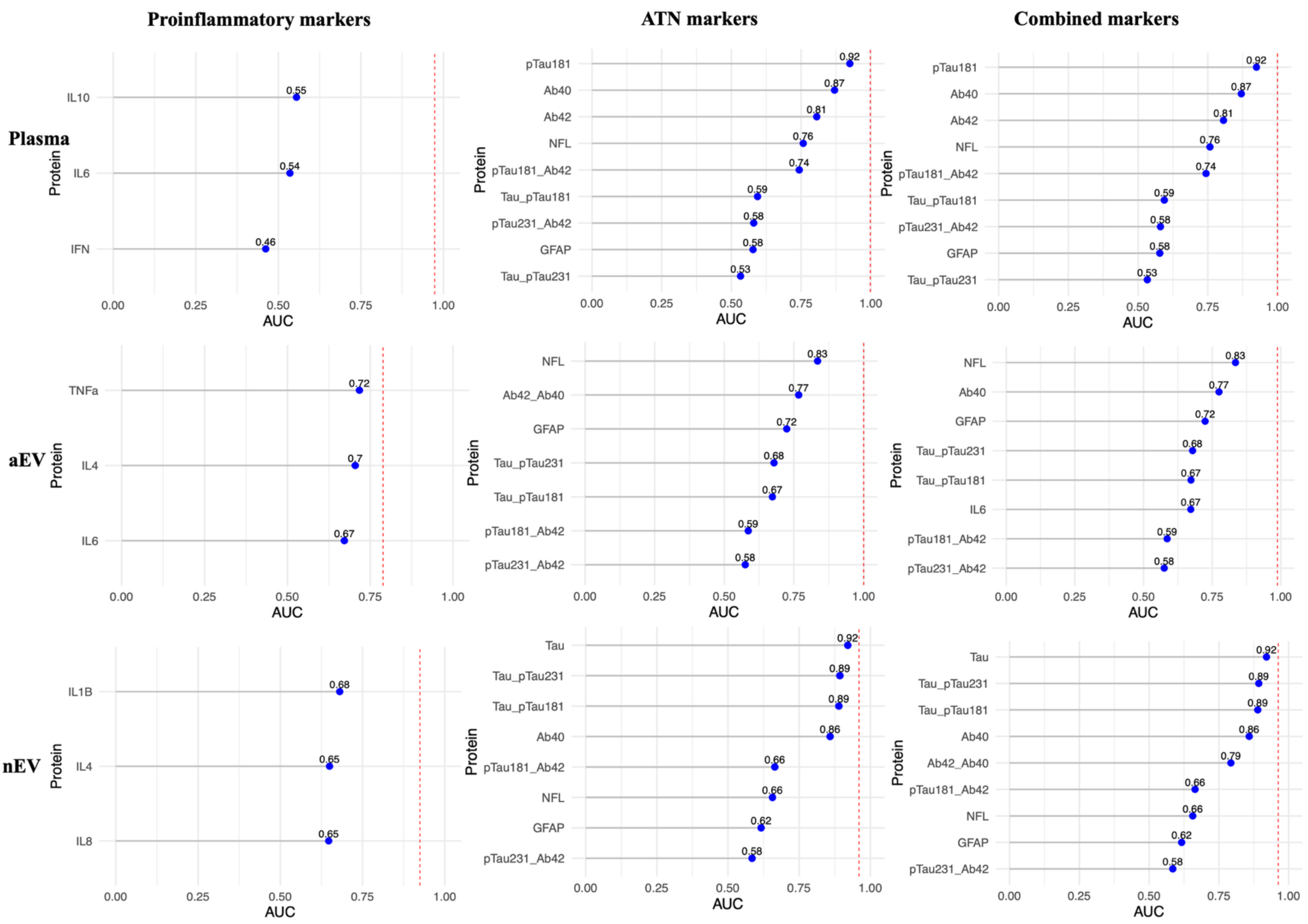

Supplemental Data: Precision Medicine for Alzheimer's Disease in Down Syndrome

b) AUCs for biomarkers predicting treatment responders (yes vs. no) in plasma, aEV, and nEV

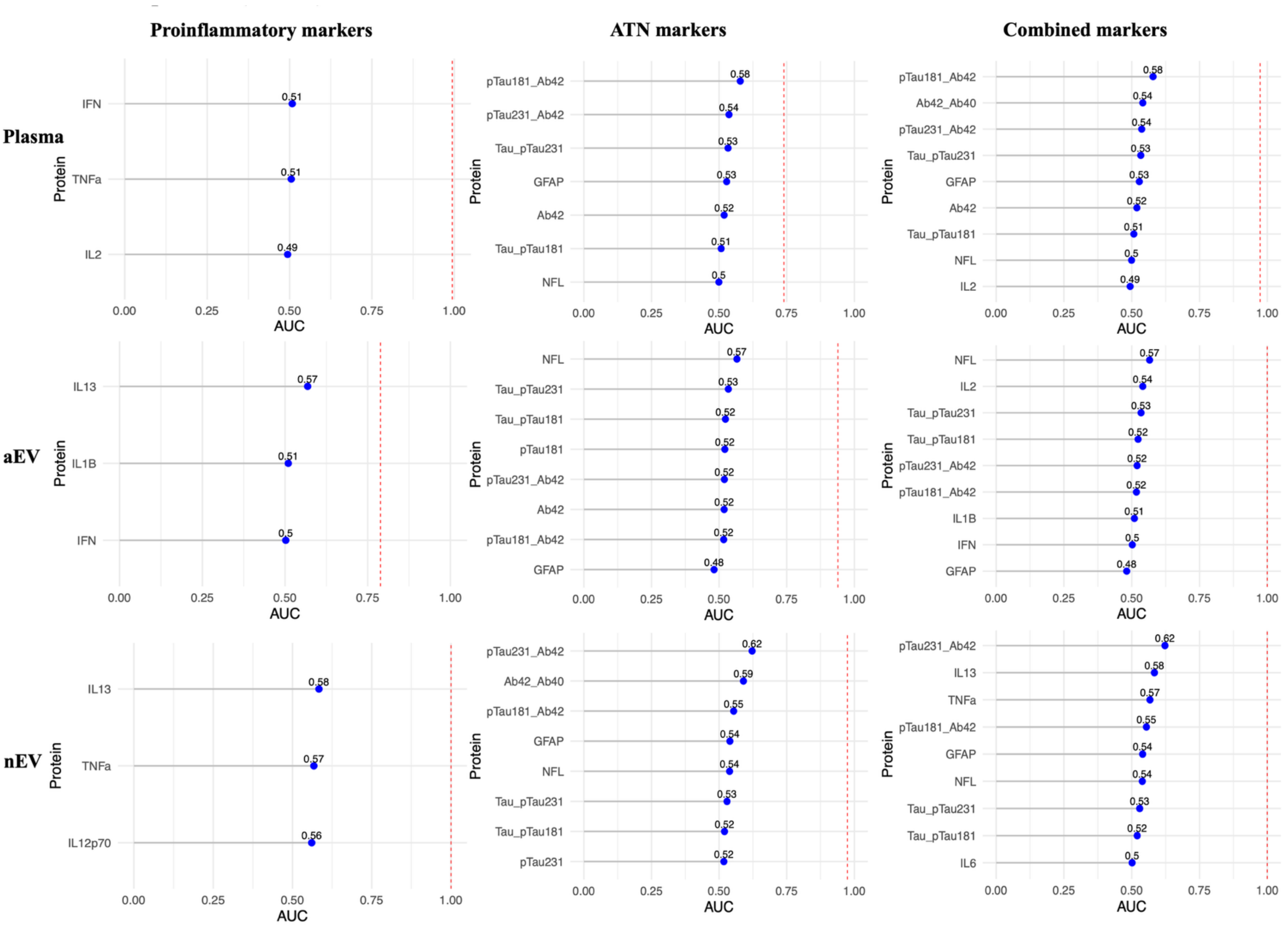

c) **AUCs for biomarkers predicting changes in treatment response over time (change vs. no change) in plasma, aEV, and nEV**

Change in Treatment Response (Changed/No Change)

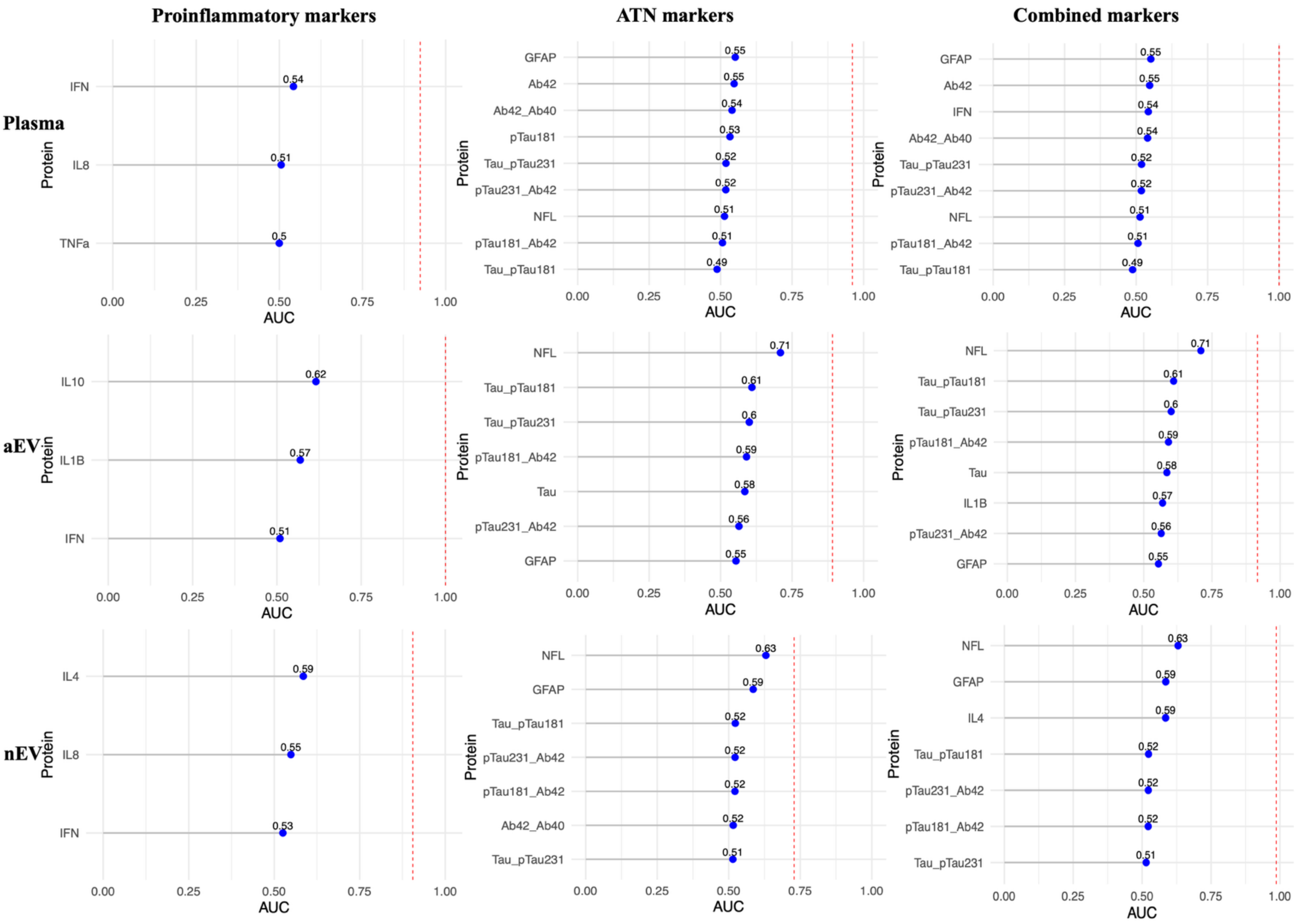

Supplementary Figure 5: **SVM analyses were conducted as a *training-only algorithm* for baseline A/T/N biomarkers in plasma (a), aEV (b), and nEV (c) to predict cognitive status (D vs. ND) in DS adults.** (**a-i**) SVM class statistics of the area under the curve, accuracy, sensitivity, specificity, and negative predictive value in plasma; (**a-ii**) SVM variable importance values plot in plasma; and (**a-iii**) ROC curve assessing the accuracy of algorithm prediction in plasma. (**b-i**) SVM class statistics of the area under the curve, accuracy, sensitivity, specificity, and negative predictive value in aEV; (**b-ii**) SVM variable importance values plot in aEV; and (**b-iii**) ROC curve assessing the accuracy of algorithm prediction in aEV. (**c-i**) SVM class statistics of the area under the curve, accuracy, sensitivity, specificity, and negative predictive value in nEV; (**c-ii**) SVM variable importance values plot in nEV; and (**c-iii**) ROC curve assessing the accuracy of algorithm prediction in nEV.

(i) (ii) (iii)

(a)

**D/ND predicted using A/T/N markers**	**Actual**	
Predicted	D	ND
D	31	2
ND	1	104
Precision/PPV	93.94%	
Accuracy	97.83%	
Sensitivity	96.88%	
Specificity	98.11%	
NPV	99.05%	
AUC	99.82%	
PPV12	87.50%	
NPV12	99.57%	

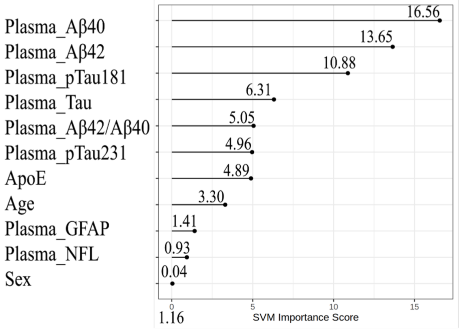

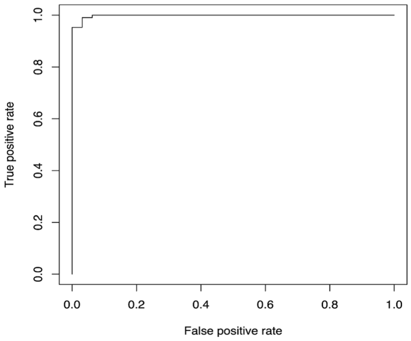

(b)

	Actual	
Predicted	D	ND
D	32	21
ND	0	85
Precision/PPV	60.38%	
Accuracy	84.78%	
Sensitivity	100.00%	
Specificity	80.19%	
NPV	100.00%	
AUC	100.00%	
PPV12	40.77%	
NPV12	100.00%	

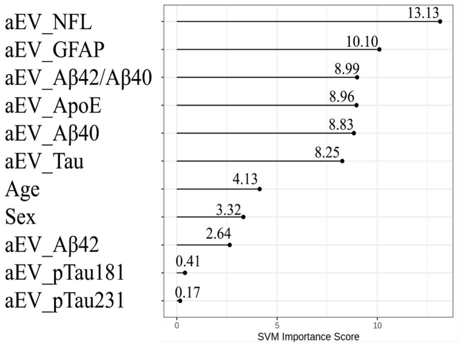

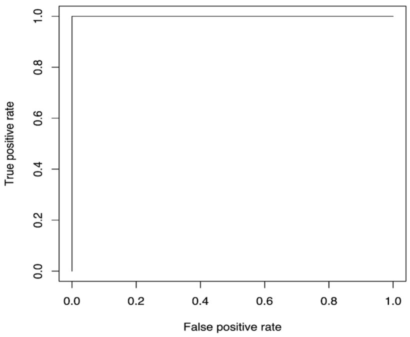

(c)

	Actual	
Predicted	D	ND
D	28	3
ND	4	103
Precision/PPV	90.32%	
Accuracy	94.93%	
Sensitivity	87.50%	
Specificity	97.17%	
NPV	96.26%	
AUC	95.99%	
PPV12	80.83%	
NPV12	98.28%	

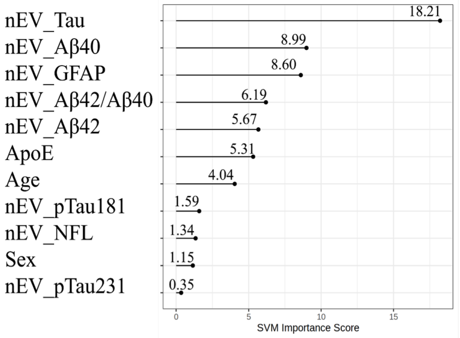

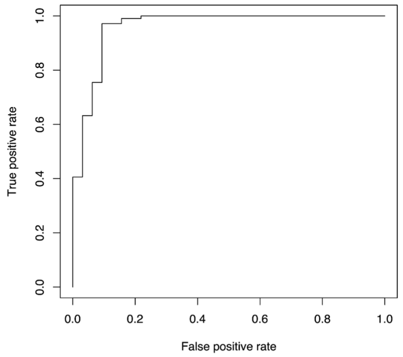

Supplemental Data: Precision Medicine for Alzheimer's Disease in Down Syndrome

Supplementary Figure 6: **SVM analyses were conducted as a *training-testing-splitting algorithm* for baseline combined proinflammatory and A/T/N biomarkers in plasma (a), aEV (b), and nEV (c) to predict cognitive status (D vs. ND) in DS adults.** (**a-i**) SVM class statistics of the area under the curve, accuracy, sensitivity, specificity, and negative predictive value in plasma; (**a-ii**) SVM variable importance values plot in plasma; and (**a-iii**) ROC curve assessing the accuracy of algorithm prediction in plasma. (**b-i**) SVM class statistics of the area under the curve, accuracy, sensitivity, specificity, and negative predictive value in aEV; (**b-ii**) SVM variable importance values plot in aEV; and (**b-iii**) ROC curve assessing the accuracy of algorithm prediction in aEV. (**c-i**) SVM class statistics of the area under the curve, accuracy, sensitivity, specificity, and negative predictive value in nEV; (**c-ii**) SVM variable importance values plot in nEV; and (**c-iii**) ROC curve assessing the accuracy of algorithm prediction in nEV.

(i) (ii) (iii)

(a)

**D/ND predicted using combined markers**	**Actual**	
Predicted	D	ND
D	32	1
ND	0	105
Precision/PPV	96.97%	
Accuracy	99.28%	
Sensitivity	100.00%	
Specificity	99.06%	
NPV	100.00%	
AUC_ROC	99.97%	
AUC_PR	99.90%	
PPV12	93.53%	
NPV12	100.00%	

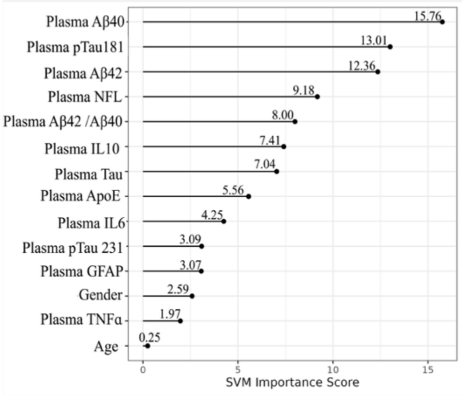

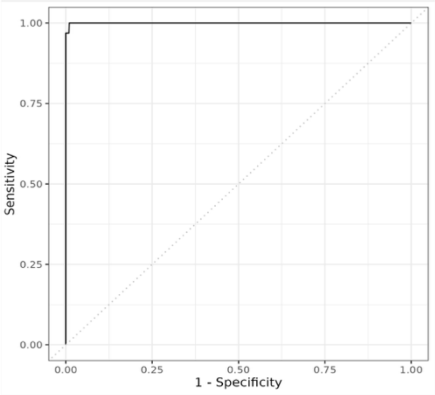

(b)

	**Actual**	
Predicted	D	ND
D	32	6
ND	0	100
Precision/PPV	84.21%	
Accuracy	95.65%	
Sensitivity	100.00%	
Specificity	94.34%	
NPV	100.00%	
AUC_ROC	99.76%	
AUC_PR	99.24%	
PPV12	70.67%	
NPV12	100.00%	

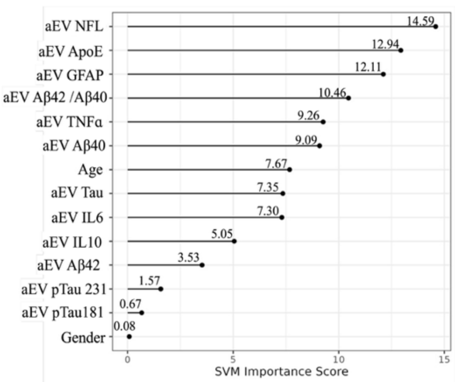

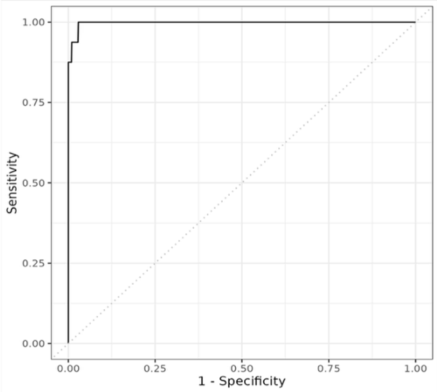

(c)

	**Actual**	
Predicted	D	ND
D	29	12
ND	3	94
Precision/PPV	70.73%	
Accuracy	89.13%	
Sensitivity	90.62%	
Specificity	88.68%	
NPV	96.91%	
AUC_ROC	95.43%	
AUC_PR	90.61%	
PPV12	52.19%	
NPV12	98.58%	

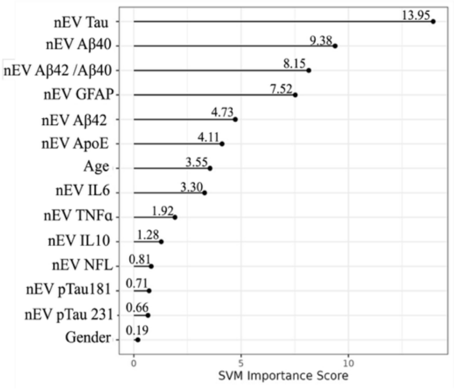

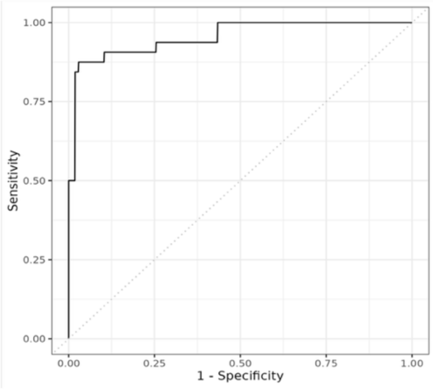

Supplementary Figure 7: **Predictive accuracy of baseline proinflammatory biomarkers for identifying responders versus non-responders (yes/no) using SVM *training-only algorithm* conducted in plasma (a), aEV (b), and nEV (c) from DS adults.** (**a-i**) SVM class statistics of the area under the curve, accuracy, sensitivity, specificity, and negative predictive value in plasma; (**a-ii**) SVM variable importance values plot in plasma; and (**a-iii**) ROC curve assessing the accuracy of algorithm prediction in plasma. (**b-i**) SVM class statistics of the area under the curve, accuracy, sensitivity, specificity, and negative predictive value in aEV; (**b-ii**) SVM variable importance values plot in aEV; and (**b-iii**) ROC curve assessing the accuracy of algorithm prediction in aEV. (**c-i**) SVM class statistics of the area under the curve, accuracy, sensitivity, specificity, and negative predictive value in nEV; (**c-ii**) SVM variable importance values plot in nEV; and (**c-iii**) ROC curve assessing the accuracy of algorithm prediction in nEV.

(i) (ii) (iii)

(a)

Treatment response predicted using proinflammatory markers	Actual	
Predicted	Yes	No
Yes	64	0
No	7	67
Precision/PPV	100.00%	
Accuracy	94.93%	
Sensitivity	90.14%	
Specificity	100.00%	
NPV	90.54%	
AUC	99.41%	
PPV12	100.00%	
NPV12	98.67%	

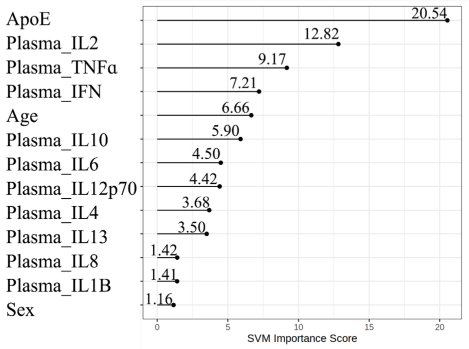

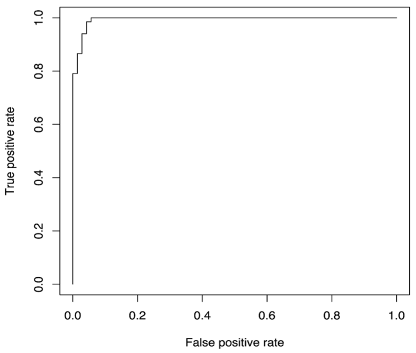

(b)

	Actual	
Predicted	Yes	No
Yes	59	25
No	12	42
Precision/PPV	70.24%	
Accuracy	73.19%	
Sensitivity	83.10%	
Specificity	62.69%	
NPV	77.78%	
AUC	78.87%	
PPV12	23.29%	
NPV12	96.45%	

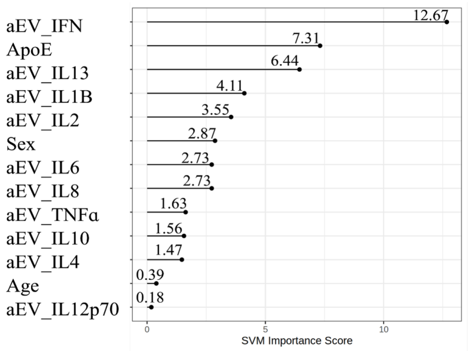

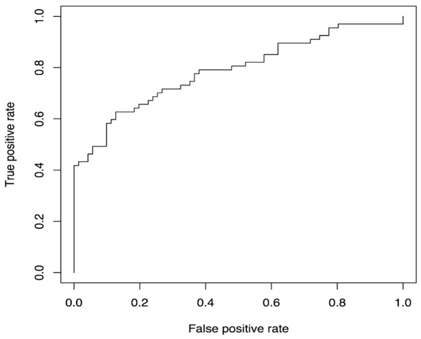

(c)

	Actual	
Predicted	Yes	No
Yes	60	0
No	11	67
Precision/PPV	100.00%	
Accuracy	92.03%	
Sensitivity	84.51%	
Specificity	100.00%	
NPV	85.90%	
AUC	100.00%	
PPV12	100.00%	
NPV12	97.93%	

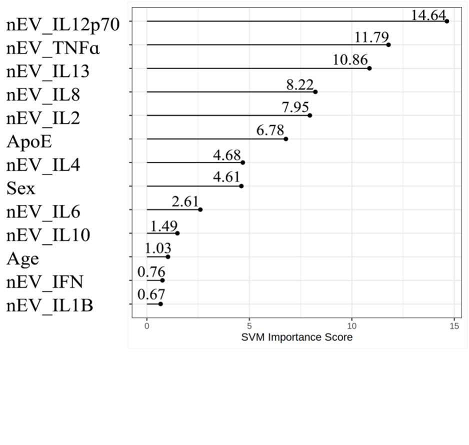

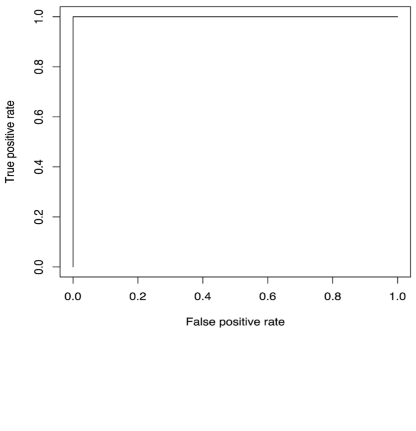

Supplementary Figure 8: **Predictive accuracy of baseline A/T/N biomarkers for identifying responders versus non-responders (yes/no) using SVM *training-only algorithm* conducted in plasma (a), aEV (b), and nEV (c) from DS adults**. (**a-i**) SVM class statistics of the area under the curve, accuracy, sensitivity, specificity, and negative predictive value in plasma; (**a-ii**) SVM variable importance values plot in plasma; and (**a-iii**) ROC curve assessing the accuracy of algorithm prediction in plasma. (**b-i**) SVM class statistics of the area under the curve, accuracy, sensitivity, specificity, and negative predictive value in aEV; (**b-ii**) SVM variable importance values plot in aEV; and (**b-iii**) ROC curve assessing the accuracy of algorithm prediction in aEV. (**c-i**) SVM class statistics of the area under the curve, accuracy, sensitivity, specificity, and negative predictive value in nEV; (**c-ii**) SVM variable importance values plot in nEV; and (**c-iii**) ROC curve assessing the accuracy of algorithm prediction in nEV.

(i) (ii) (iii)

(a)

Treatment response predicted using A/T/N markers	Actual	
Predicted	Yes	No
Yes	45	14
No	26	53
Precision/PPV	76.27%	
Accuracy	71.01%	
Sensitivity	63.38%	
Specificity	79.10%	
NPV	67.09%	
AUC	73.98%	
PPV12	29.26%	
NPV12	94.06%	

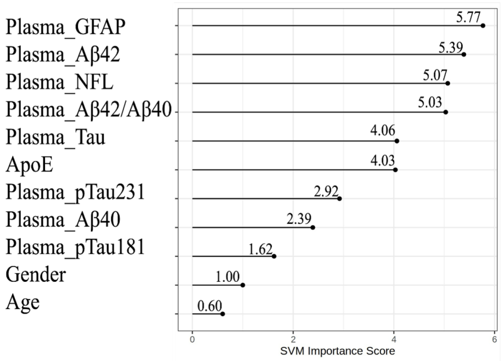

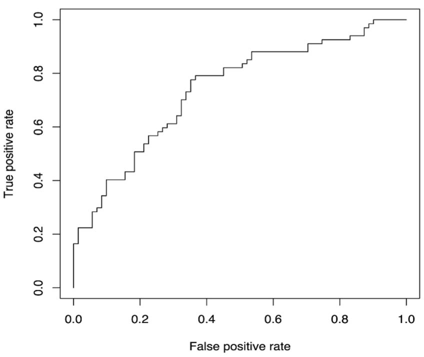

(b)

	Actual	
Predicted	Yes	No
Yes	71	23
No	0	44
Precision/PPV	75.53%	
Accuracy	83.33%	
Sensitivity	100.00%	
Specificity	65.67%	
NPV	100.00%	
AUC	93.88%	
PPV12	28.43%	
NPV12	100.00%	

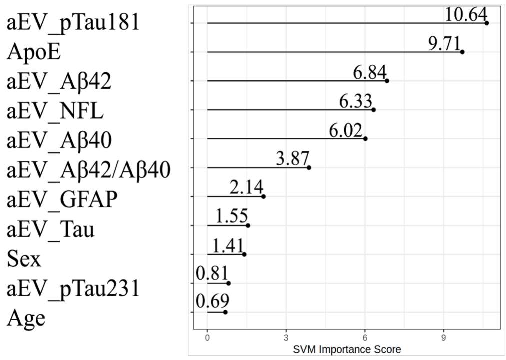

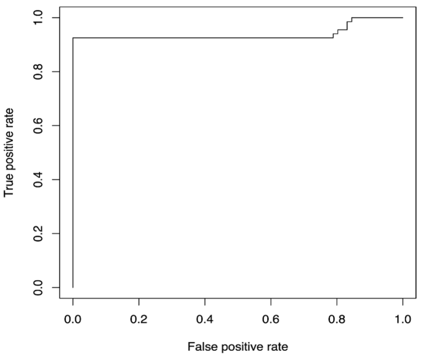

(c)

	Actual	
Predicted	Yes	No
Yes	53	0
No	18	67
Precision/PPV	100.00%	
Accuracy	86.96%	
Sensitivity	74.65%	
Specificity	100.00%	
NPV	78.82%	
AUC	97.41%	
PPV12	100.00%	
NPV12	96.66%	

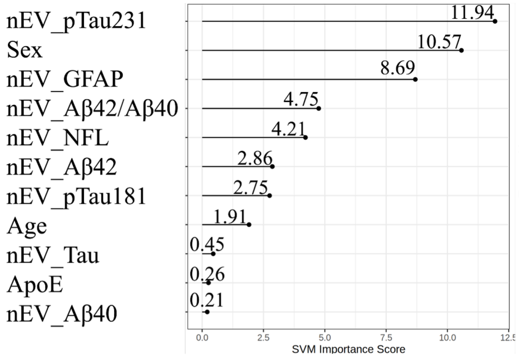

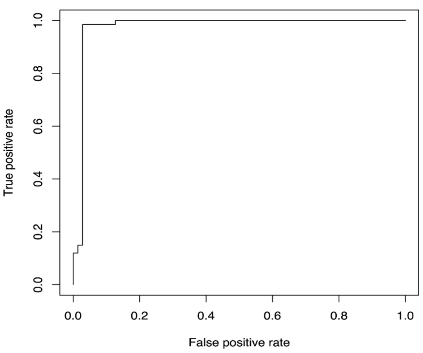

Supplemental Data: Precision Medicine for Alzheimer's Disease in Down Syndrome

Supplementary Figure 9: **Predictive accuracy of proinflammatory biomarkers in identifying changes in treatment response vs. no change from baseline (0) to 36 months using SVM *training-only algorithm* analyses in plasma (a), aEV (b), and nEV (c) from DS adults.** (**a-i**) SVM class statistics of the area under the curve, accuracy, sensitivity, specificity, and negative predictive value in plasma; (**a-ii**) SVM variable importance values plot in plasma; and (**a-iii**) ROC curve assessing the accuracy of algorithm prediction in plasma. (**b-i**) SVM class statistics of the area under the curve, accuracy, sensitivity, specificity, and negative predictive value in aEV; (**b-ii**) SVM variable importance values plot in aEV; and (**b-iii**) ROC curve assessing the accuracy of algorithm prediction in aEV. (**c-i**) SVM class statistics of the area under the curve, accuracy, sensitivity, specificity, and negative predictive value in nEV; (**c-ii**) SVM variable importance values plot in nEV; and (**c-iii**) ROC curve assessing the accuracy of algorithm prediction in nEV.

(i) (ii) (iii)

(a)

Change in treatment response predicted using proinflammatory markers	Actual	
Predicted	Changed	No change
Changed	24	0
No change	48	66
Precision/PPV	100.00%	
Accuracy	65.22%	
Sensitivity	33.33%	
Specificity	100.00%	
NPV	57.89%	
AUC	92.36%	
PPV12	100.00%	
NPV12	91.67%	

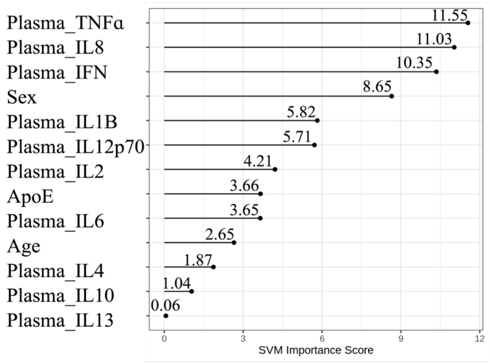

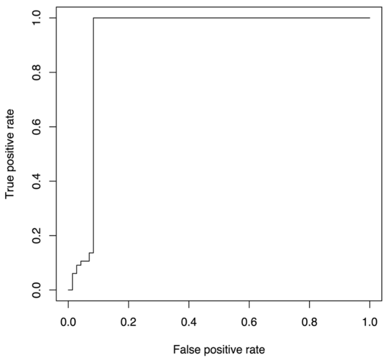

(b)

	Actual	
Predicted	Changed	No change
Changed	69	0
Nochange	3	66
Precision/PPV	100.00%	
Accuracy	97.83%	
Sensitivity	95.83%	
Specificity	100.00%	
NPV	95.65%	
AUC	100.00%	
PPV12	100.00%	
NPV12	99.44%	

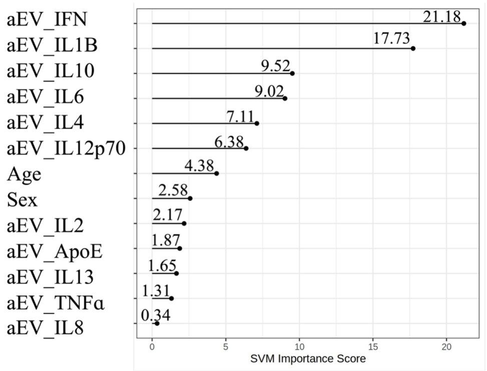

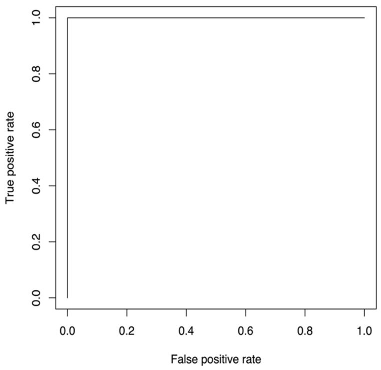

(c)

	Actual	
Predicted	Changed	No change
Changed	72	50
Nochange	0	16
Precision/PPV	59.02%	
Accuracy	63.77%	
Sensitivity	100.00%	
Specificity	24.24%	
NPV	100.00%	
AUC	90.53%	
PPV12	15.25%	
NPV12	100.00%	

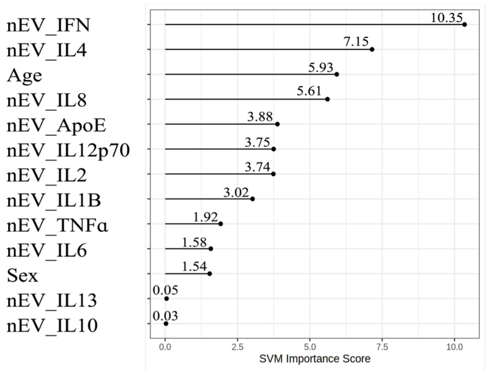

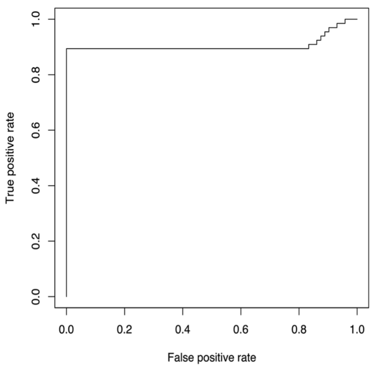

Supplementary Figure 10: **Predictive accuracy of A/T/N biomarkers in identifying changes in treatment response vs. no change from baseline (0) to 36 months using SVM *training-only algorithm* analyses in plasma (a), aEV (b), and nEV (c) from DS adults.** (**a-i**) SVM class statistics of the area under the curve, accuracy, sensitivity, specificity, and negative predictive value in plasma; (**a-ii**) SVM variable importance values plot in plasma; and (**a-iii**) ROC curve assessing the accuracy of algorithm prediction in plasma. (**b-i**) SVM class statistics of the area under the curve, accuracy, sensitivity, specificity, and negative predictive value in aEV; (**b-ii**) SVM variable importance values plot in aEV; and (**b-iii**) ROC curve assessing the accuracy of algorithm prediction in aEV. (**c-i**) SVM class statistics of the area under the curve, accuracy, sensitivity, specificity, and negative predictive value in nEV; (**c-ii**) SVM variable importance values plot in nEV; and (**c-iii**) ROC curve assessing the accuracy of algorithm prediction in nEV.

(i) (ii) (iii)

(a)

Change in treatment response predicted using A/T/N markers	Actual	
Predicted	Changed	No change
Changed	55	3
No change	17	63
Precision/PPV	94.83%	
Accuracy	85.51%	
Sensitivity	76.39%	
Specificity	95.45%	
NPV	78.75%	
AUC	96.06%	
PPV12	69.62%	
NPV12	96.74%	

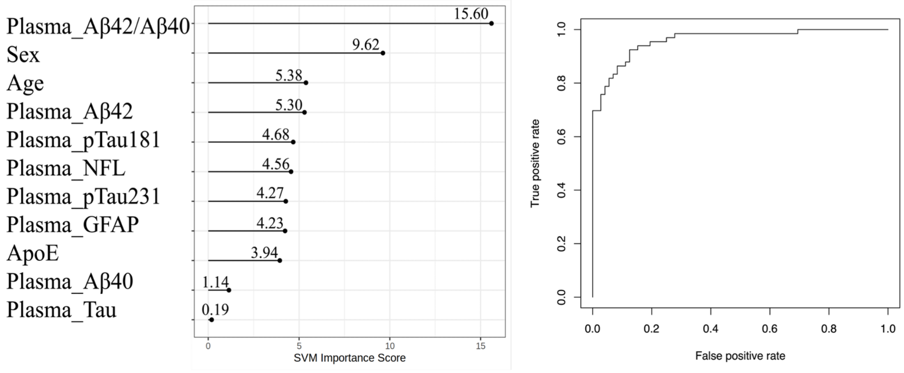

(b)

	Actual	
Predicted	Changed	No change
Changed	63	23
No change	9	43
Precision/PPV	73.26%	
Accuracy	76.81%	
Sensitivity	87.50%	
Specificity	65.15%	
NPV	82.69%	
AUC	89.10%	
PPV12	25.51%	
NPV12	97.45%	

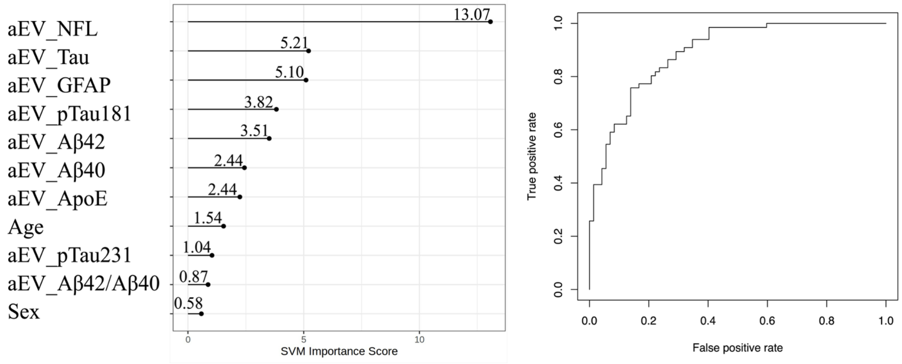

(c)

	Actual	
Predicted	Changed	No change
Changed	54	28
No change	18	38
Precision/PPV	65.85%	
Accuracy	66.67%	
Sensitivity	75.00%	
Specificity	57.58%	
NPV	67.86%	
AUC	72.87%	
PPV12	19.42%	
NPV12	94.41%	

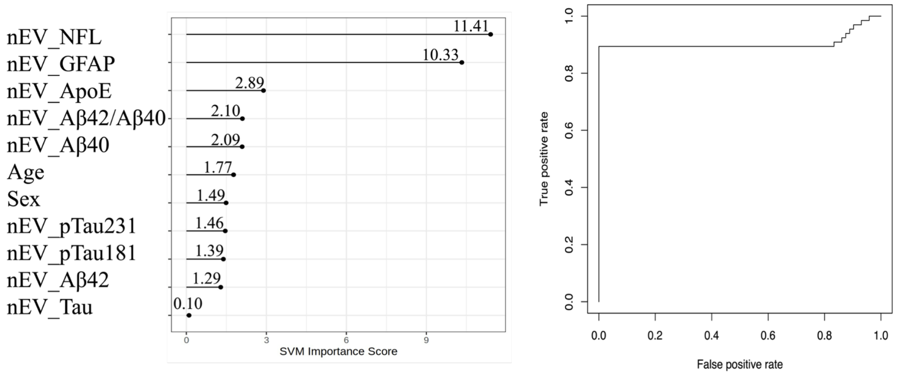

Supplementary Figure 11: Scatter plots showing the relationship between log-transformed levels of the top three biomarkers and the annual rate of cognitive change, as measured by a composite of primary and secondary endpoints (BPT, VT, and BFDS) within the Vitamin E trial in individuals with DS, in predicting the cognitive status, treatment responders, and changes in treatment response from baseline (0) to 36 months. (a-c) Top three biomarkers associated with cognitive status (demented [D] vs. non-demented [ND]) in plasma (**a**), astrocyte-derived extracellular vesicles (aEV; **b**), and neuron-derived extracellular vesicles (nEV; **c**). (**d-f**) Biomarkers predictive of treatment response (yes vs. no) in plasma (**d**), aEV (**e**), and nEV **(f)**. (**g-i**) Biomarkers associated with changes in treatment response over time (change vs. no change) in plasma (**g**), aEV (h), and nEV **(i)**. Each point represents an individual participant, with biomarker levels plotted against the annualized rate of cognitive change. These correlations highlight the potential of these biomarkers to track disease progression and treatment-related effects in the DS population.

a) **<u>Scatter plots showing the relationship between log-transformed levels of the top 3 biomarkers and the annual rate of cognitive change predicting cognitive status (demented [D] vs. non-demented [ND]) in plasma</u>**

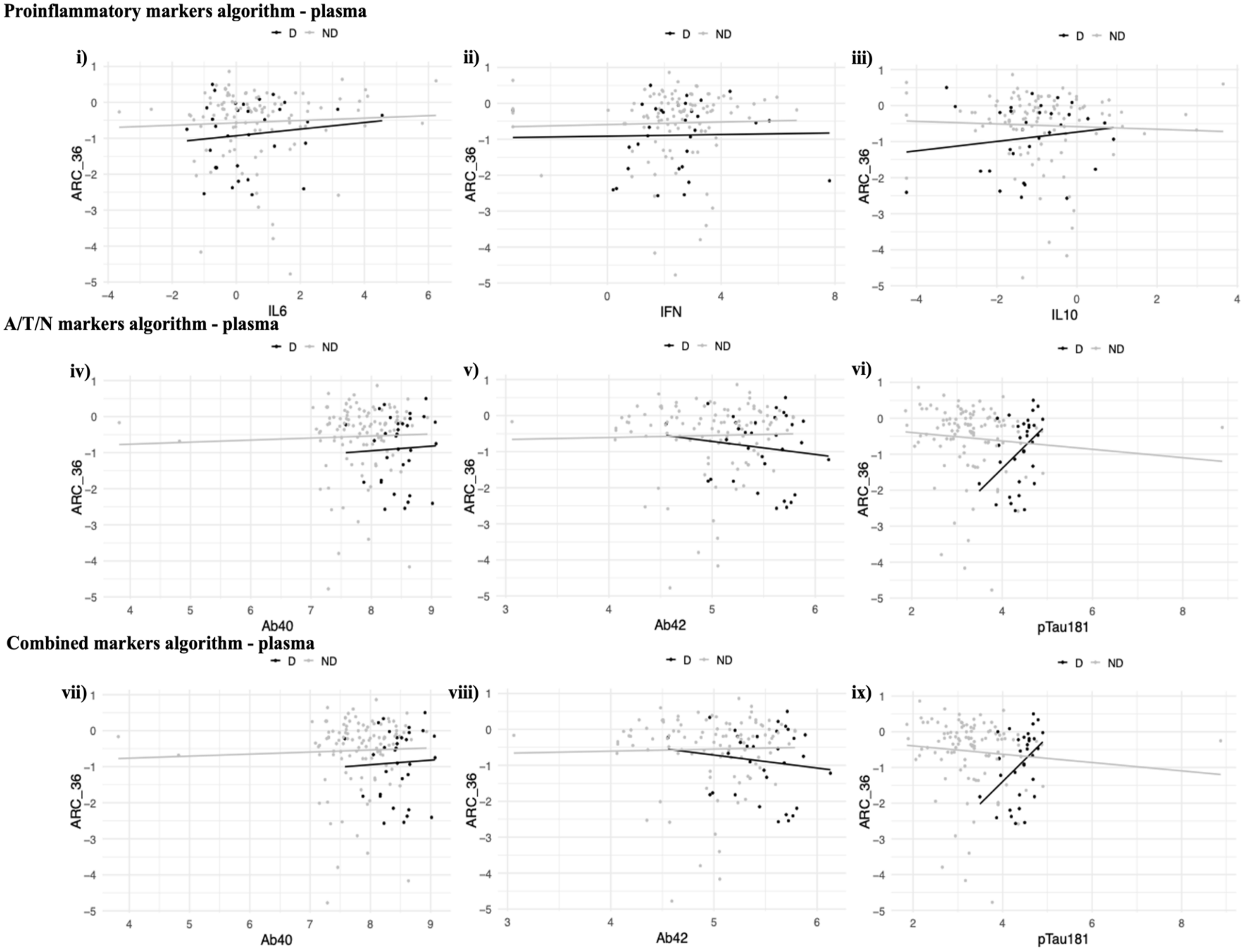

Supplemental Data: Precision Medicine for Alzheimer's Disease in Down Syndrome

b) **Scatter plots showing the relationship between log-transformed levels of the top 3 biomarkers and the annual rate of cognitive change predicting cognitive status (demented [D] vs. non-demented [ND]) in aEV**

Proinflammatory markers algorithm - aEV

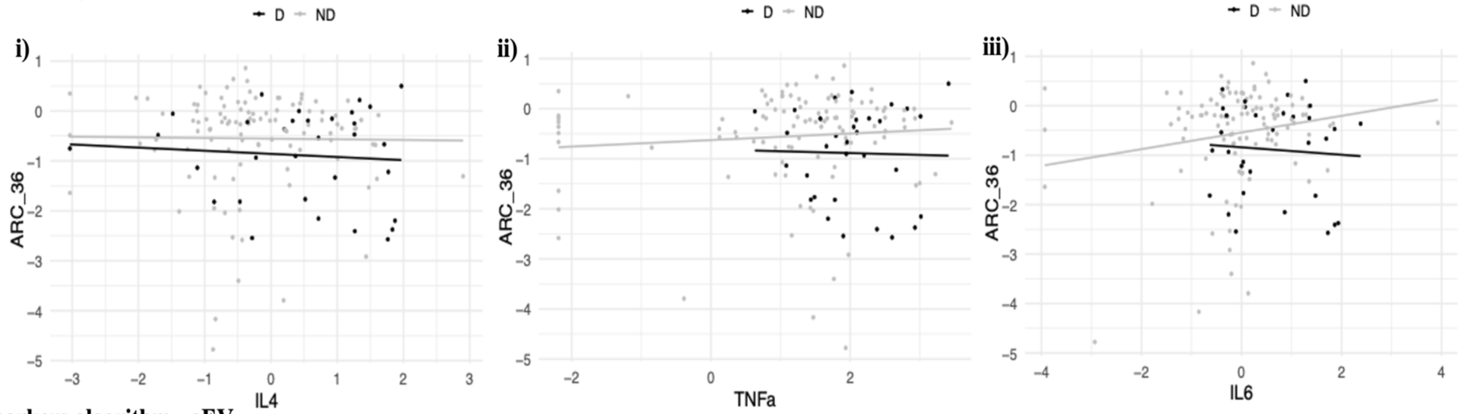

A/T/N markers algorithm - aEV

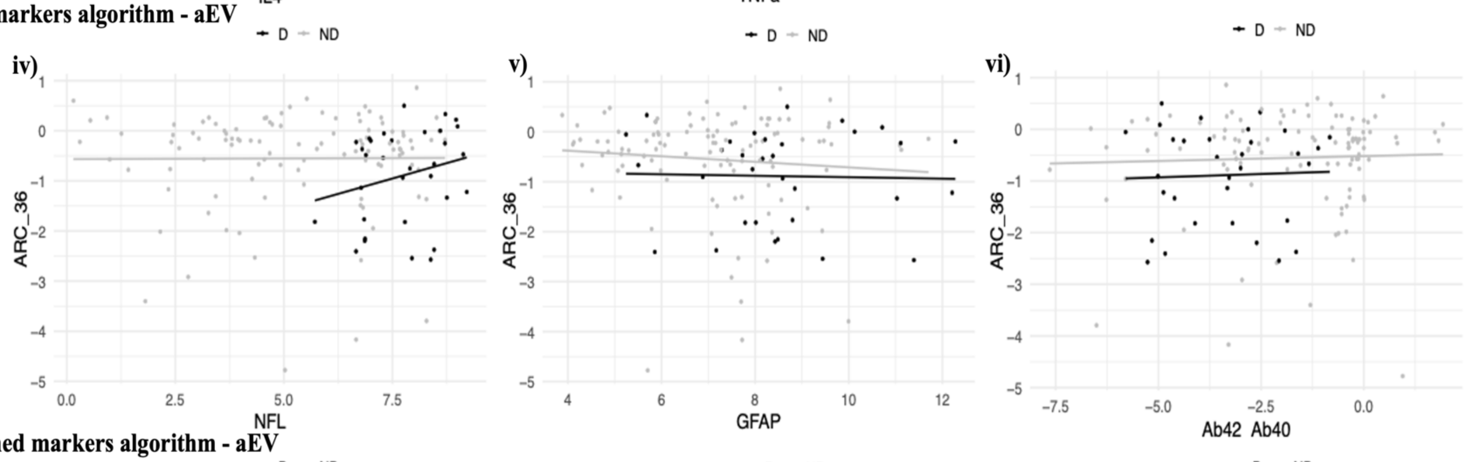

Combined markers algorithm - aEV

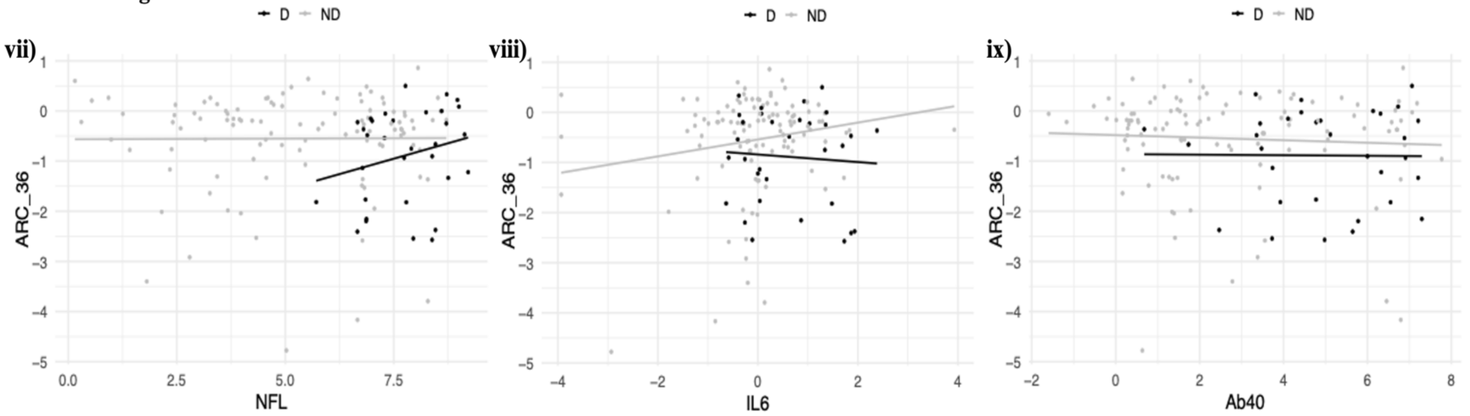

c) **Scatter plots showing the relationship between log-transformed levels of the top 3 biomarkers and the annual rate of cognitive change predicting cognitive status (demented [D] vs. non-demented [ND]) in nEV**

Proinflammatory markers algorithm - nEV

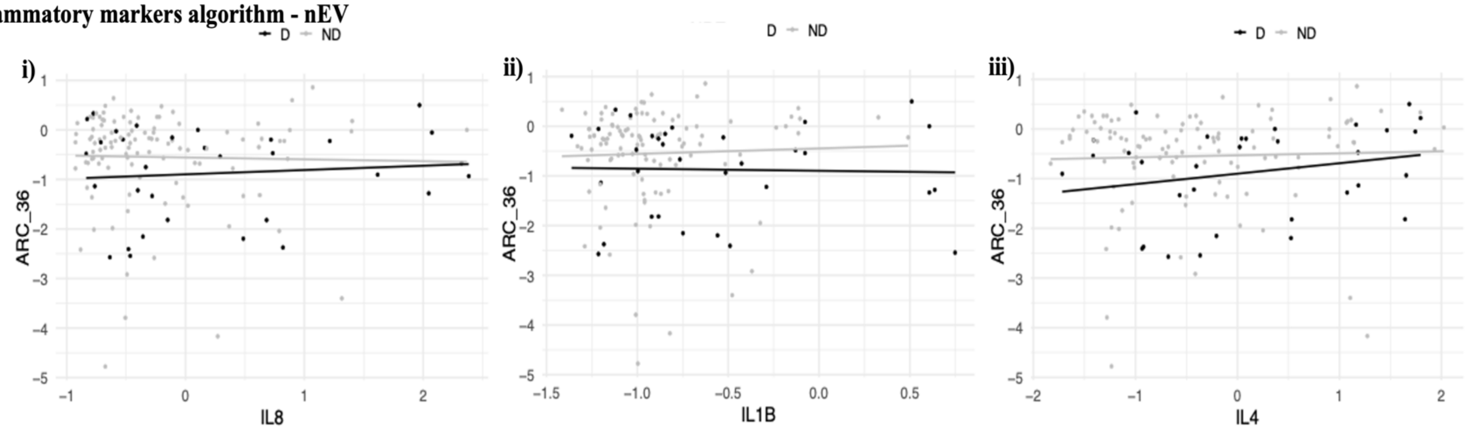

A/T/N markers algorithm - nEV

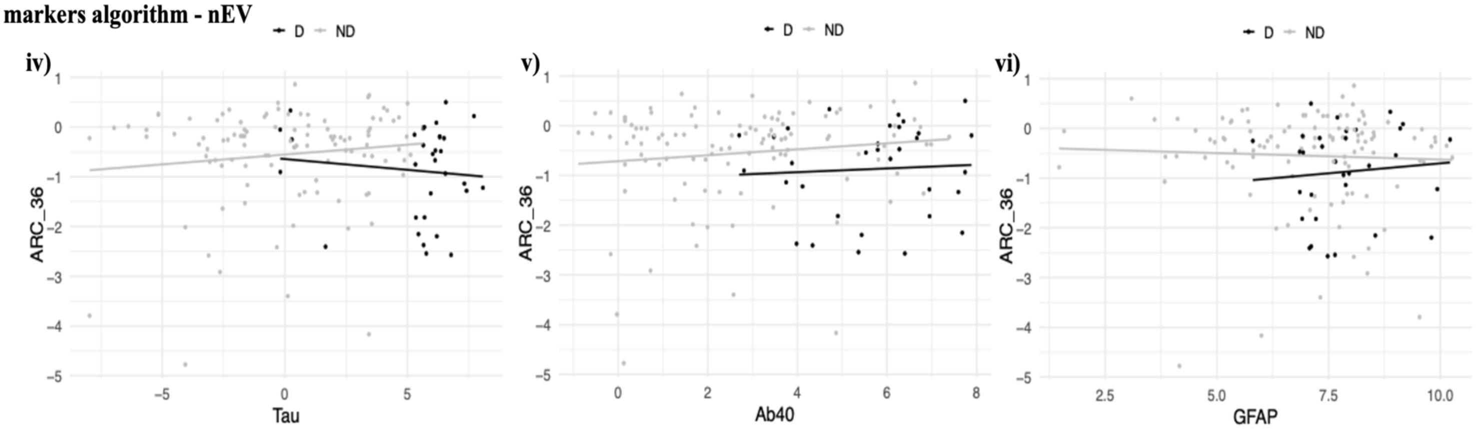

Combined markers algorithm - nEV

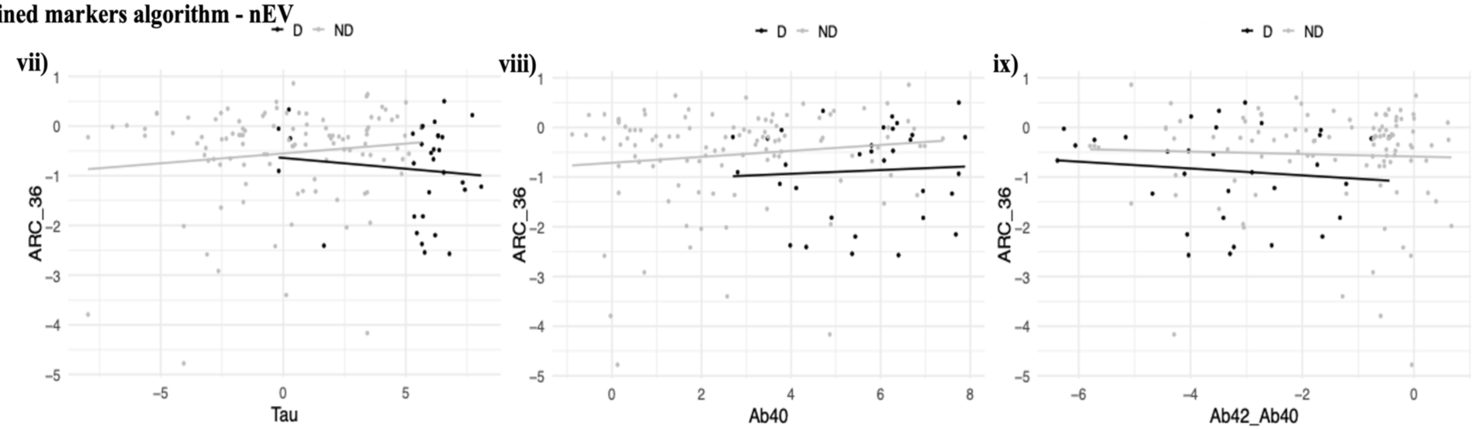

Supplemental Data: Precision Medicine for Alzheimer's Disease in Down Syndrome

d) **Scatter plots showing the relationship between log-transformed levels of the top 3 biomarkers and the annual rate of cognitive change predicting treatment responders (yes vs. no) in plasma**

Proinflammatory markers algorithm - plasma

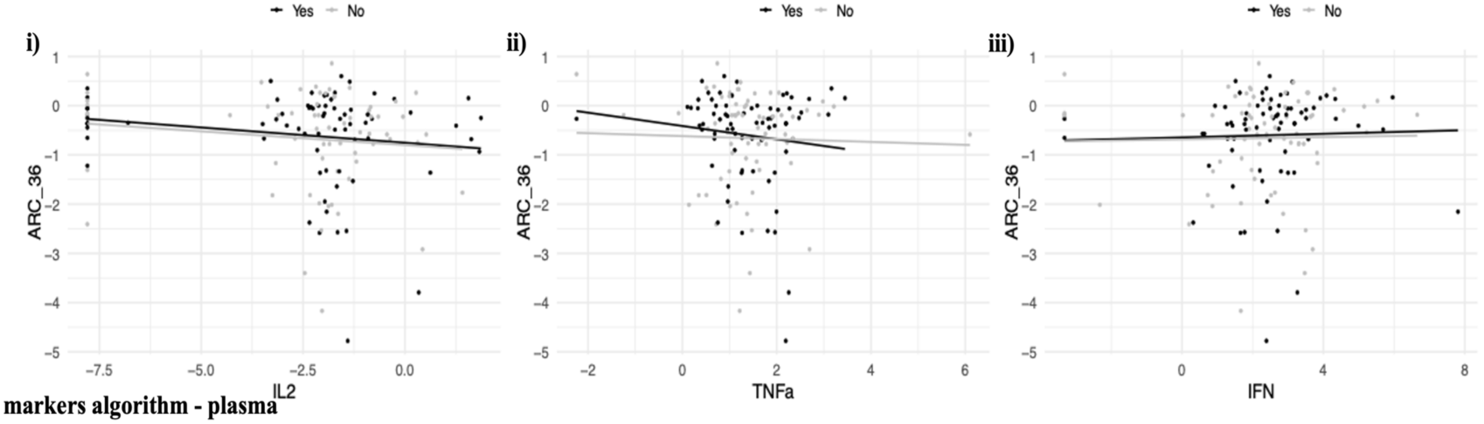

A/T/N markers algorithm - plasma

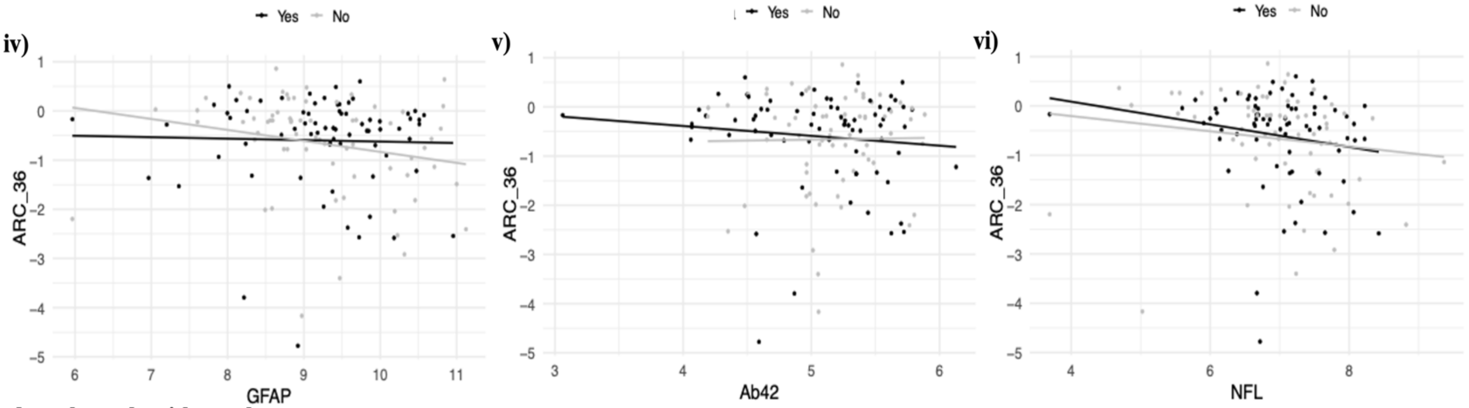

Combined markers algorithm - plasma

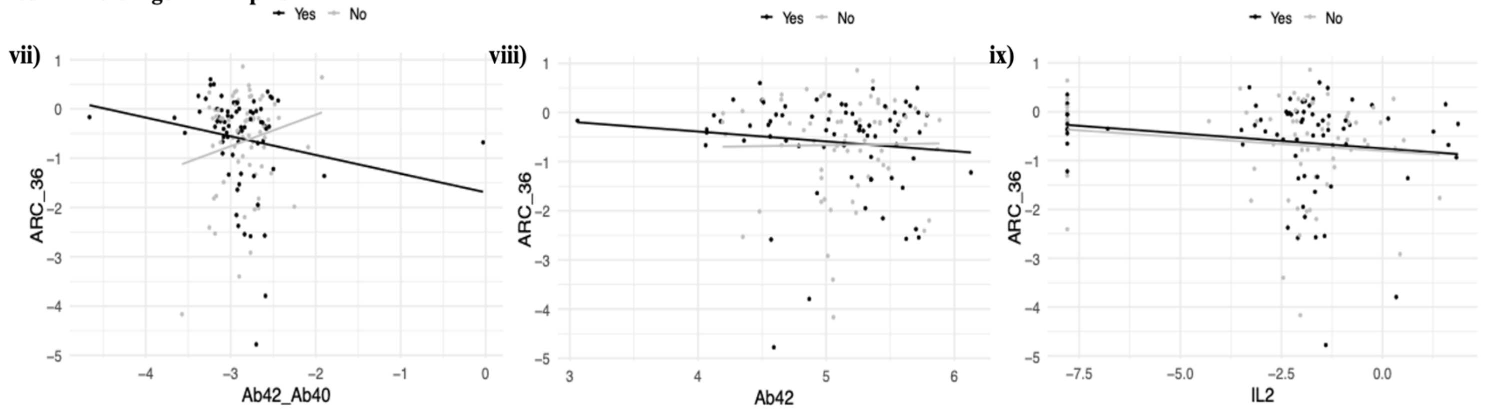

e) **<u>Scatter plots showing the relationship between log-transformed levels of the top 3 biomarkers and the annual rate of cognitive change predicting treatment responders (yes vs. no) in aEV</u>**

Proinflammatory markers algorithm - aEV

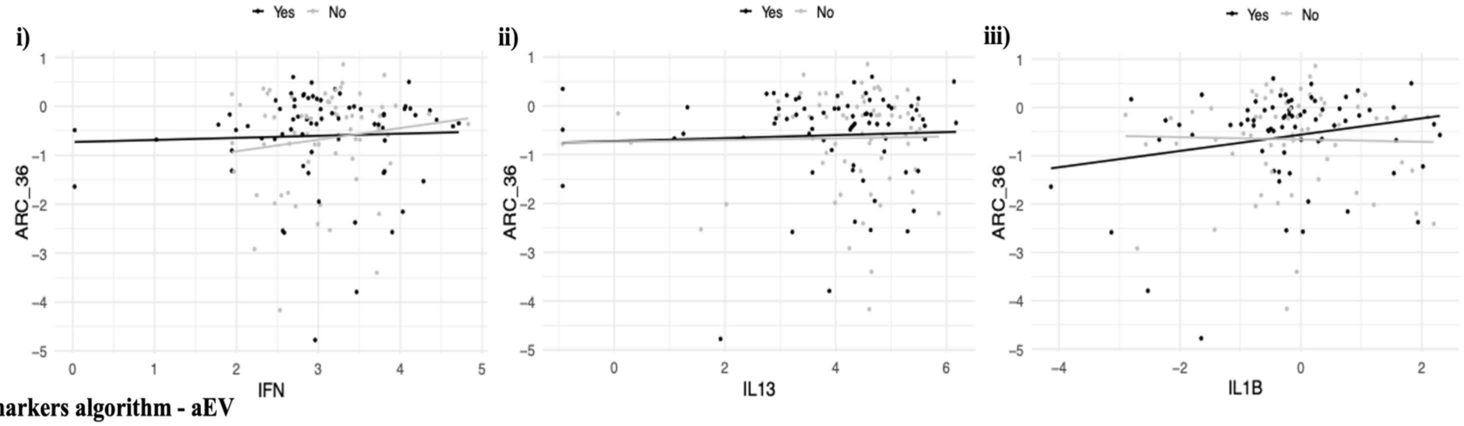

A/T/N markers algorithm - aEV

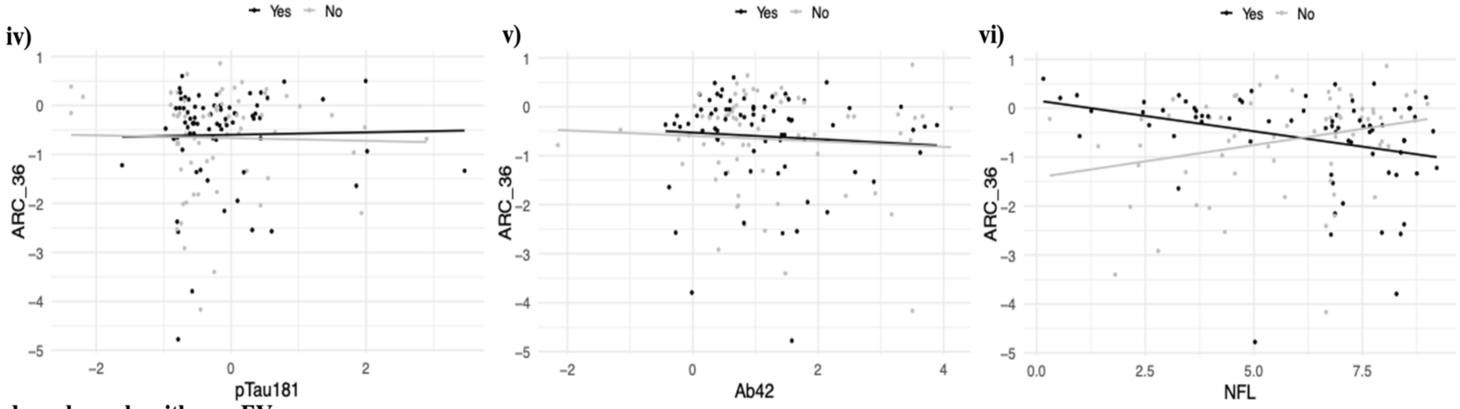

Combined markers algorithm - aEV

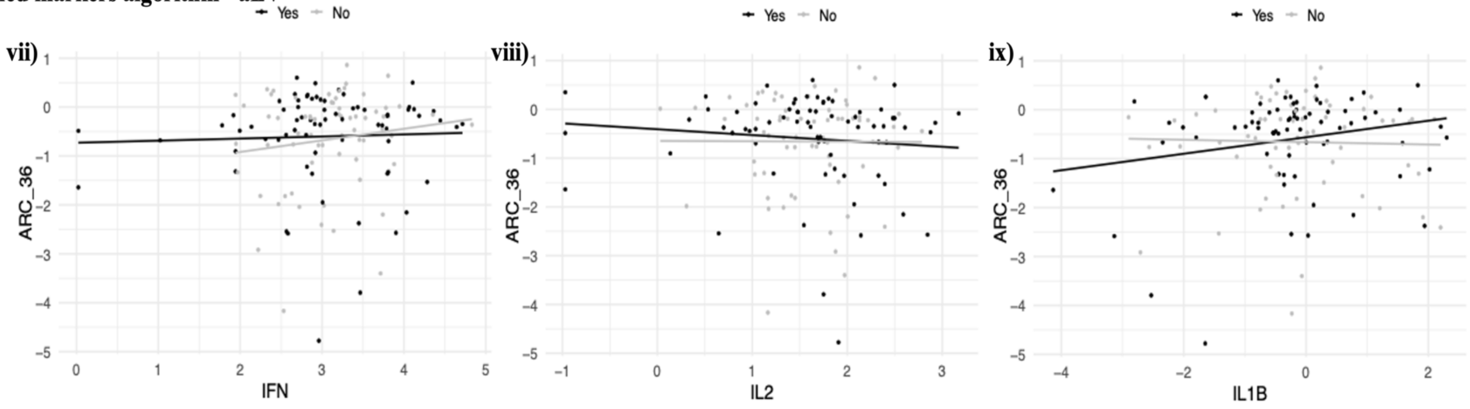

Supplemental Data: Precision Medicine for Alzheimer's Disease in Down Syndrome

f) <u>Scatter plots showing the relationship between log-transformed levels of the top 3 biomarkers and the annual rate of cognitive change predicting treatment responders (yes vs. no) in nEV</u>

Proinflammatory markers algorithm - nEV

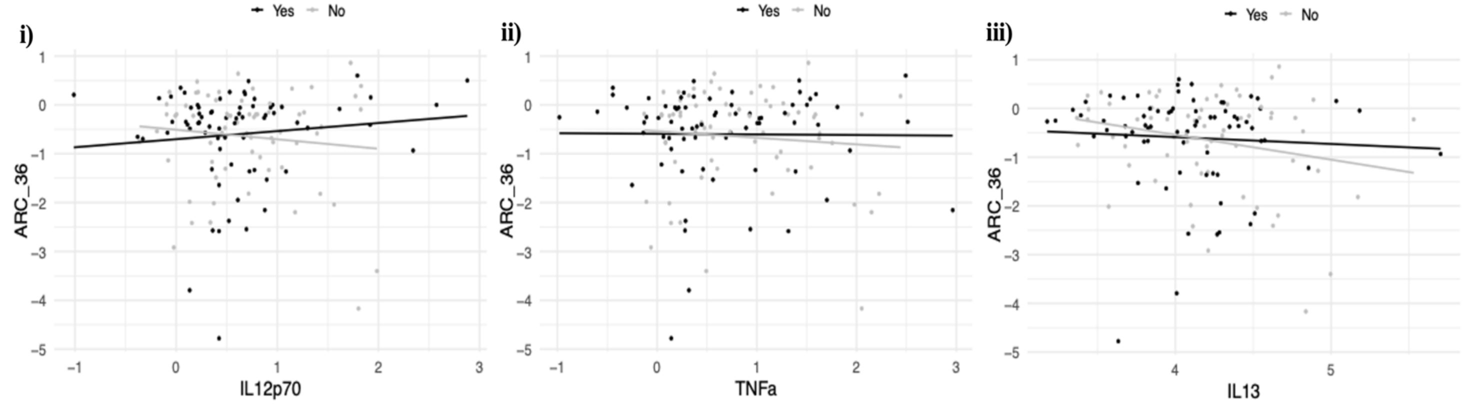

A/T/N markers algorithm - nEV

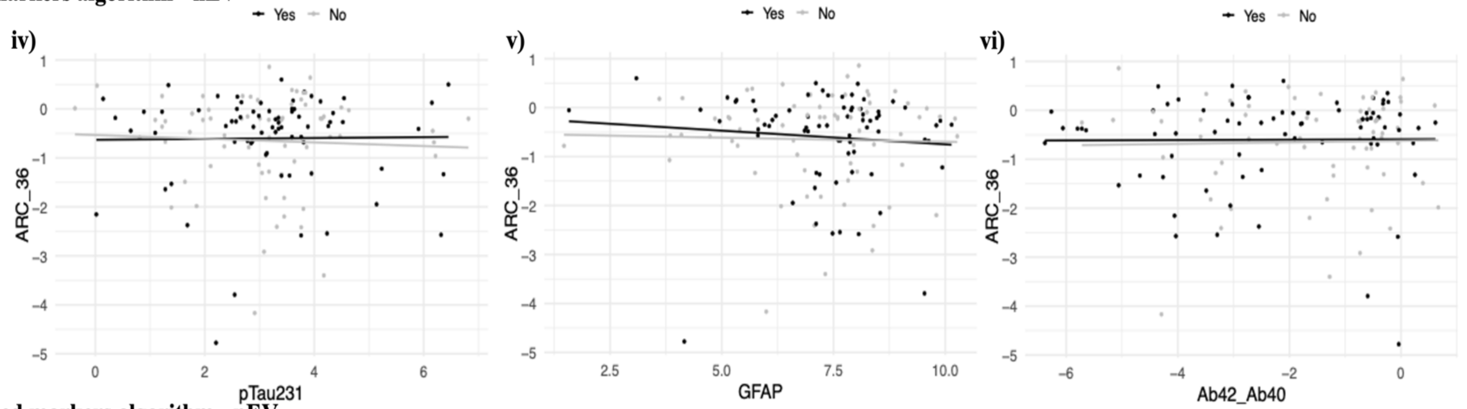

Combined markers algorithm - nEV

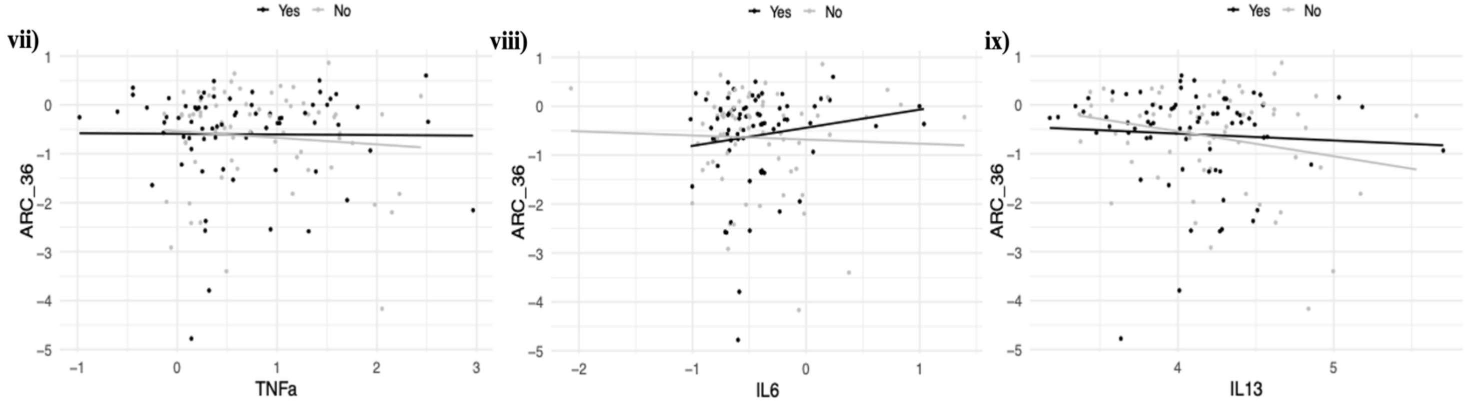

Supplemental Data: Precision Medicine for Alzheimer's Disease in Down Syndrome

g) **Scatter plots showing the relationship between log-transformed levels of the top 3 biomarkers and the annual rate of cognitive change predicting change in treatment response (changed vs. no change) in plasma**

Proinflammatory markers algorithm - plasma

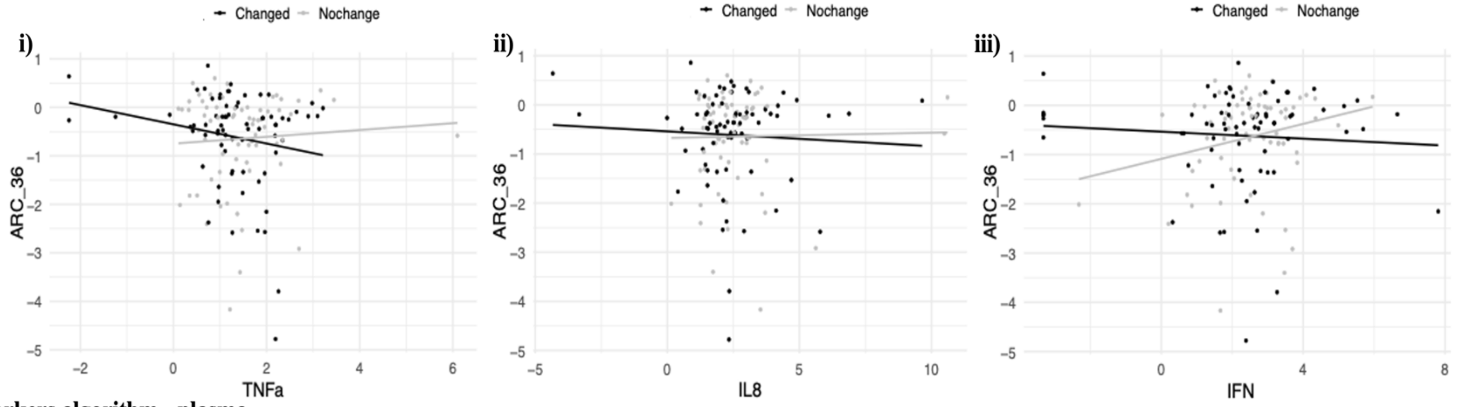

A/T/N markers algorithm - plasma

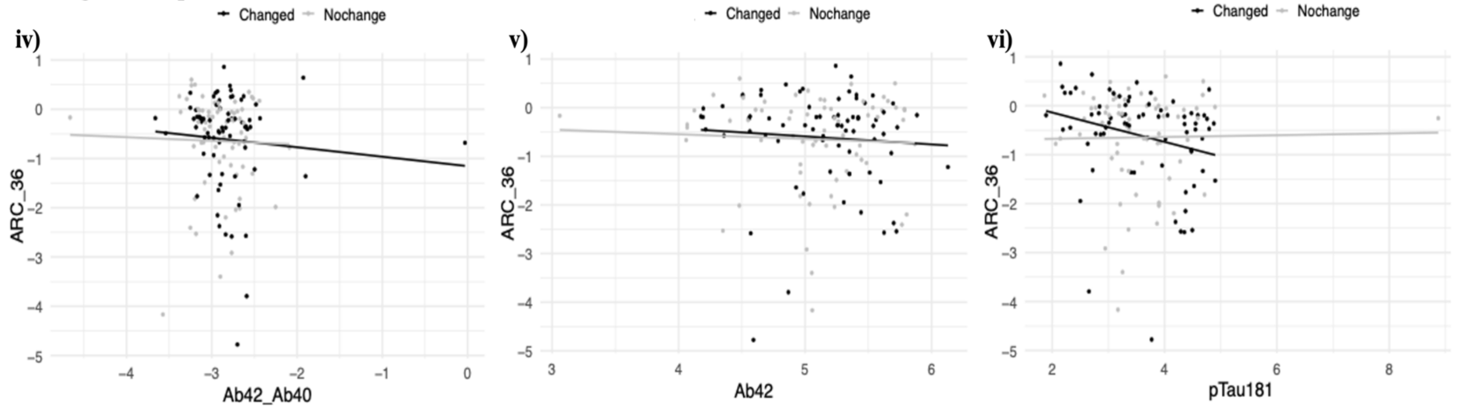

Combined markers algorithm - plasma

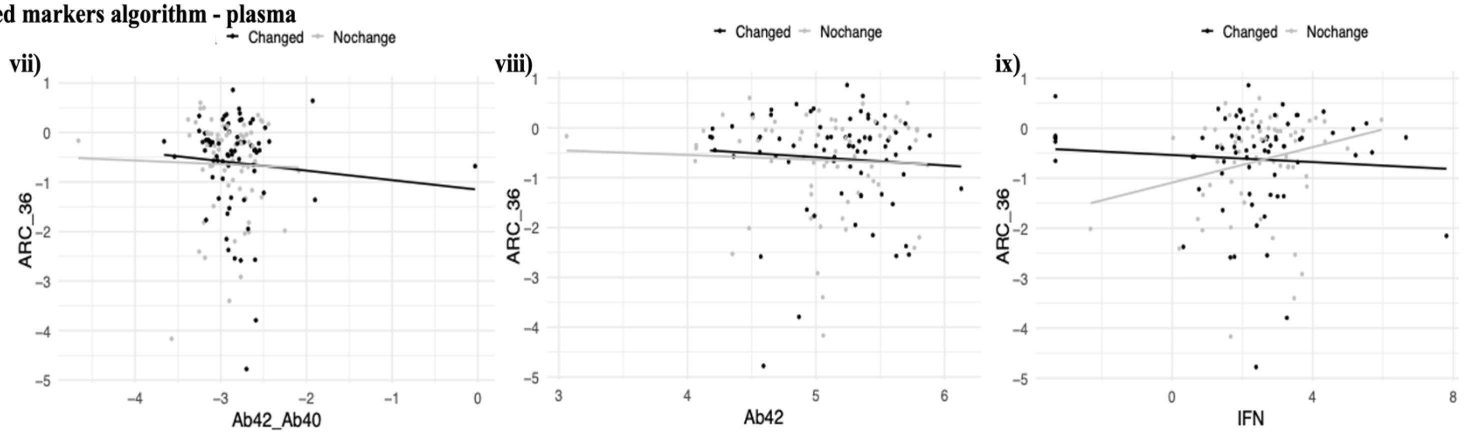

h) Scatter plots showing the relationship between log-transformed levels of the top 3 biomarkers and the annual rate of cognitive change predicting change in treatment response (changed vs. no change) in aEV

Proinflammatory markers algorithm - aEV

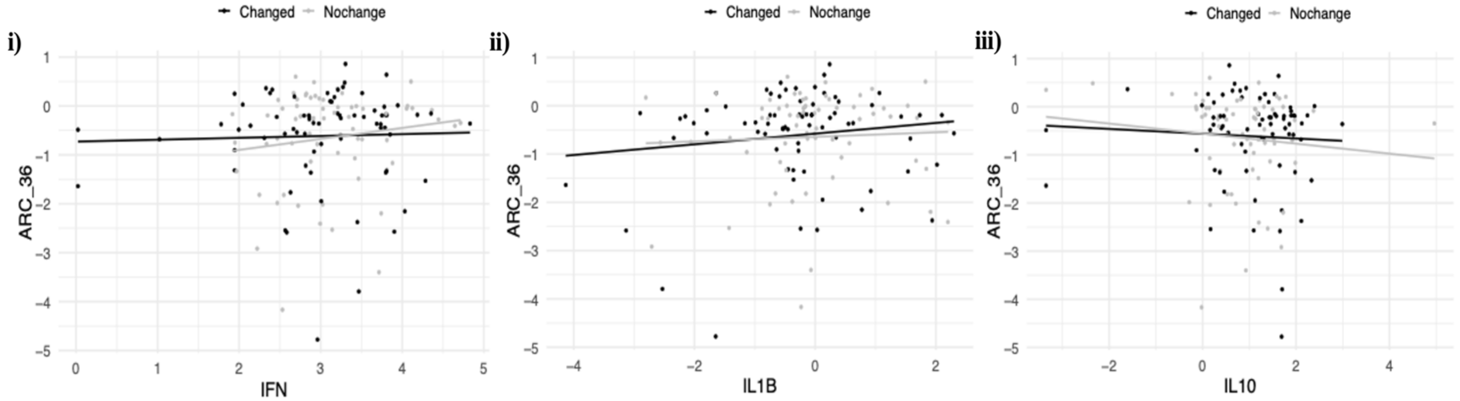

A/T/N markers algorithm - aEV

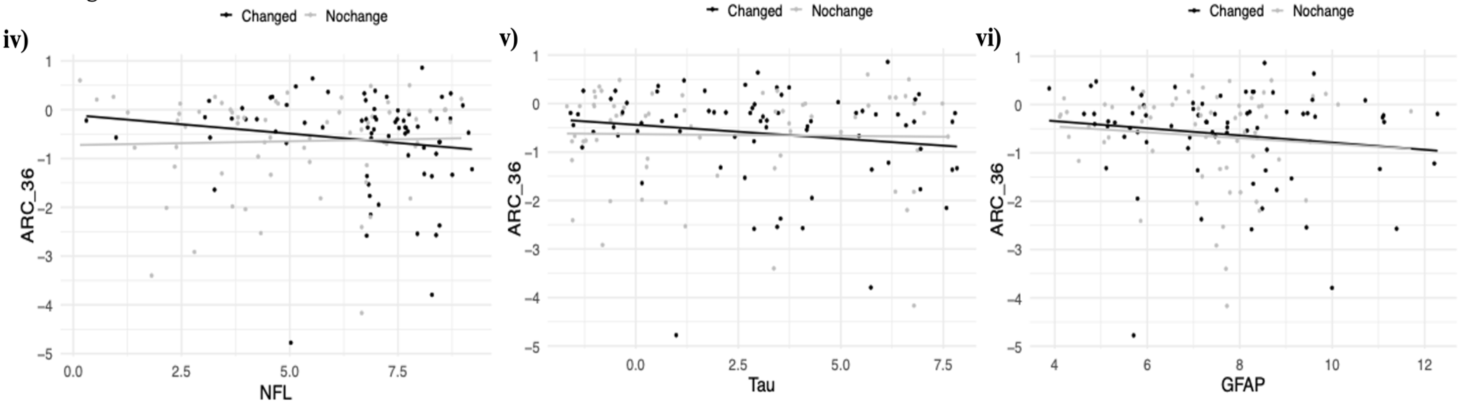

Combined markers algorithm - aEV

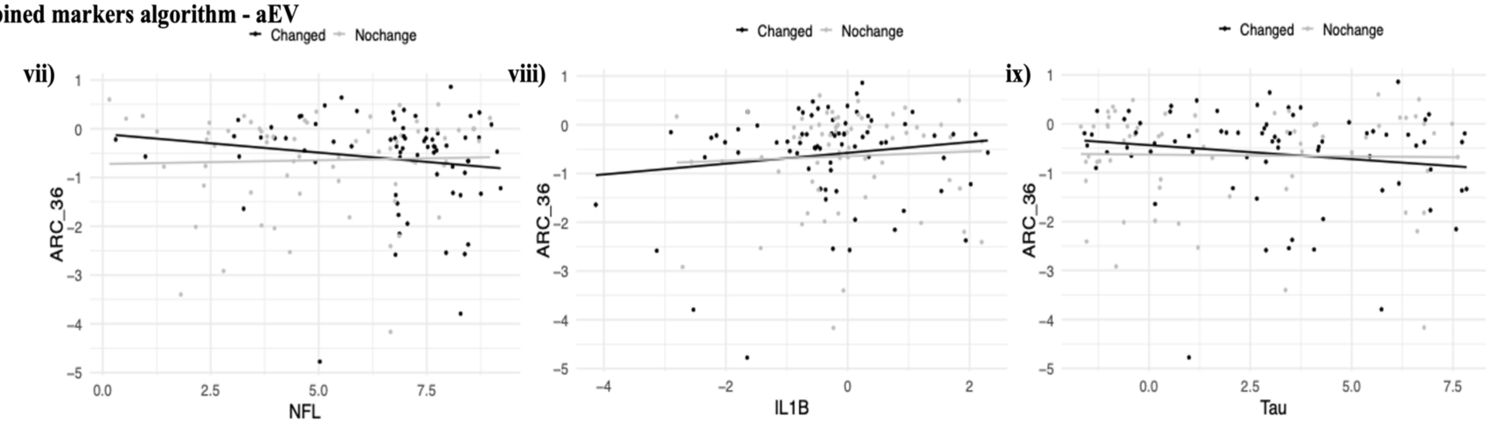

Supplemental Data: Precision Medicine for Alzheimer's Disease in Down Syndrome

i) **<u>Scatter plots showing the relationship between log-transformed levels of the top 3 biomarkers and the annual rate of cognitive change predicting change in treatment response (changed vs. no change) in nEV</u>**

Proinflammatory markers algorithm - nEV

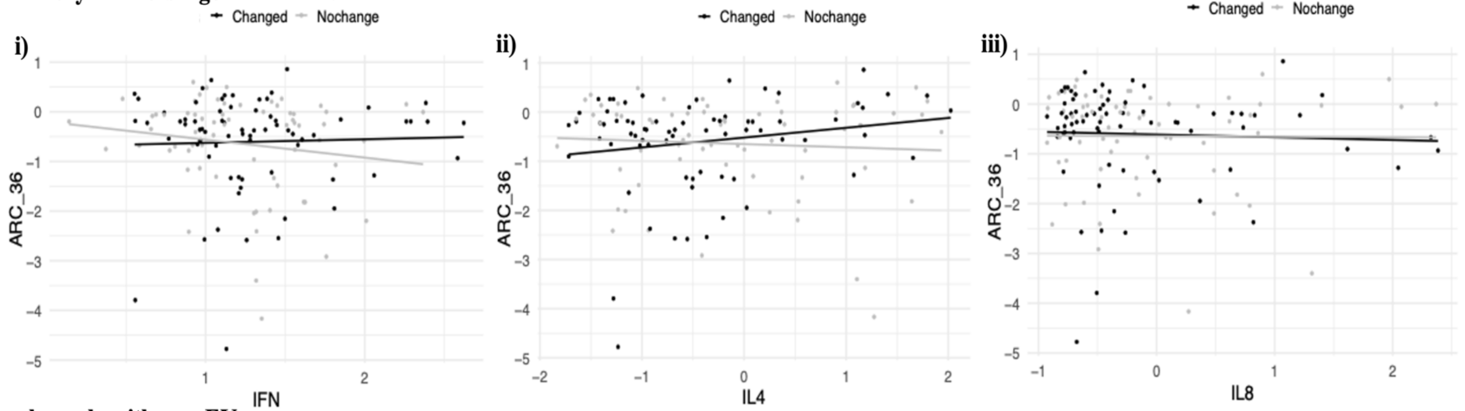

A/T/N markers algorithm - nEV

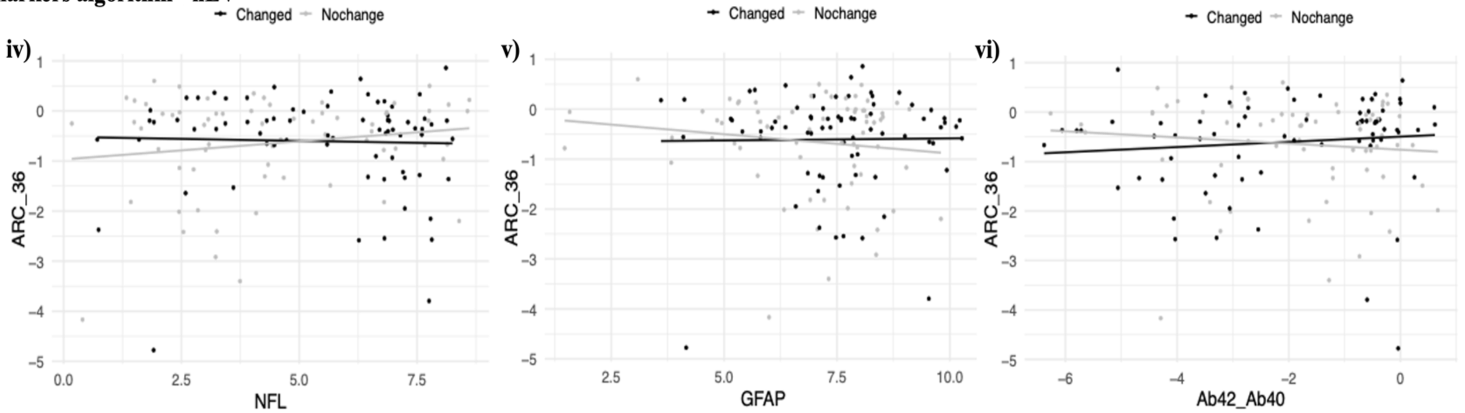

Combined markers algorithm - nEV

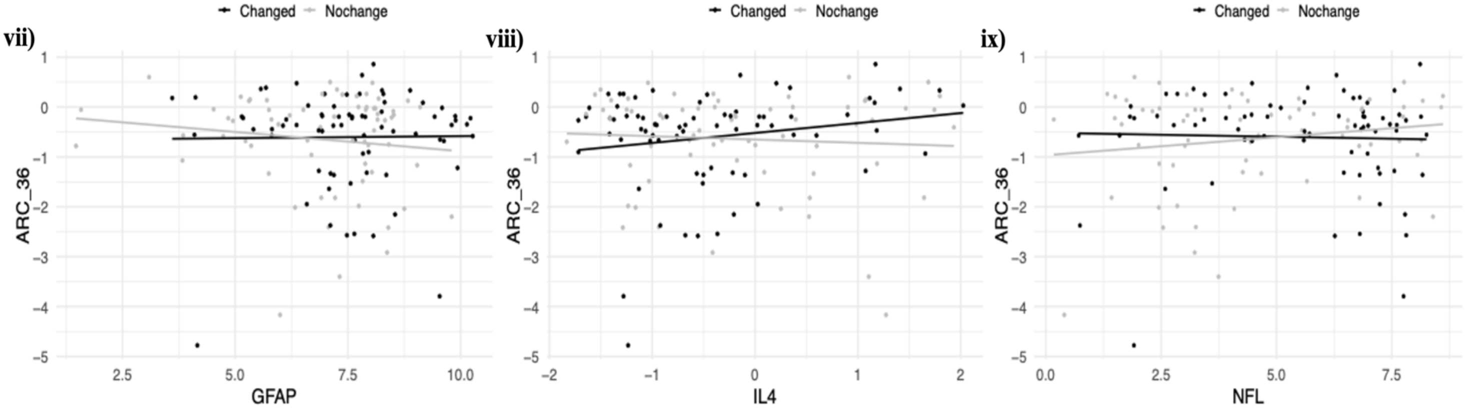

Supplemental Data: Precision Medicine for Alzheimer's Disease in Down Syndrome

Supplementary Figure 12: Box plots of log-transformed values of the top three biomarker concentration levels in respective biofluids predicting cognitive status, treatment responders, and changes in treatment response over time (change/no change) in treatment response. (**a-c**) Log-transformed levels of the top three biomarkers associated with prediction of cognitive status (demented [D] vs. non-demented [ND]) across plasma (**a**), aEV (**b**), and nEV (**c**). (**d-f**) Biomarkers predictive of treatment responders (yes vs. no) in plasma (**d**), aEV (**e**), and nEV (**f**). (**g-i**) Biomarkers associated with changes in treatment response over time (change vs. no change) in plasma (**g**), aEV (**h**), and nEV (**i**). Each box represents the interquartile range, with the horizontal line indicating the median. Data points outside this range are shown as individual dots.

a) **<u>Boxplot of log-transformed values of the top 3 biomarkers predictive of cognitive status (demented [D] vs. non-demented [ND]) in plasma</u>**

Proinflammatory markers algorithm - plasma

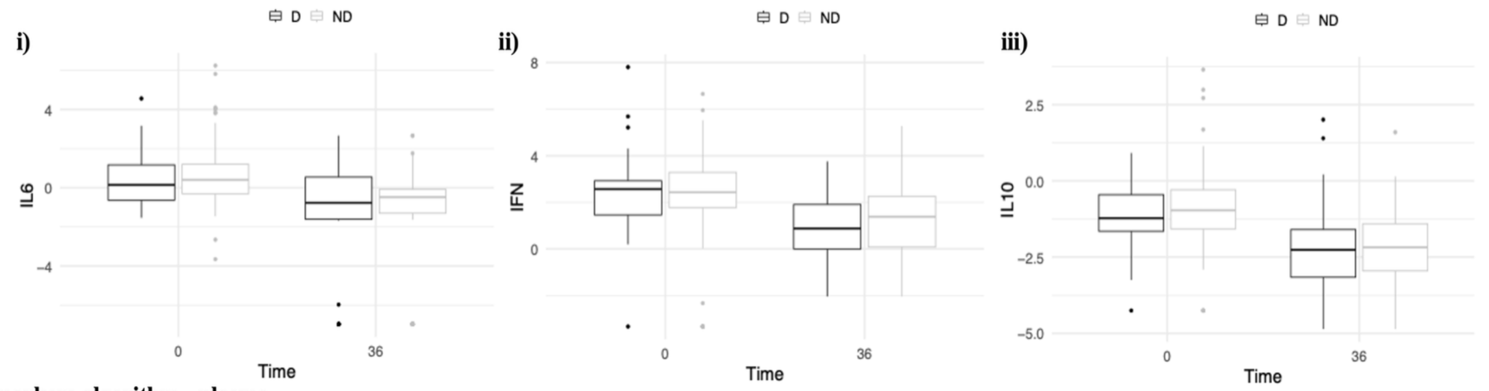

A/T/N markers algorithm - plasma

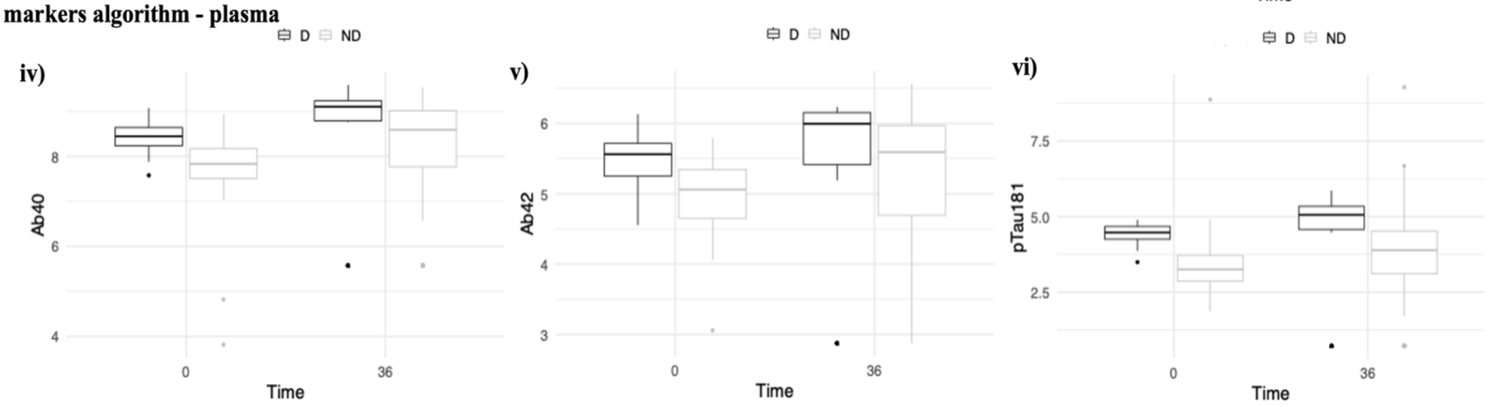

Combined markers algorithm - plasma

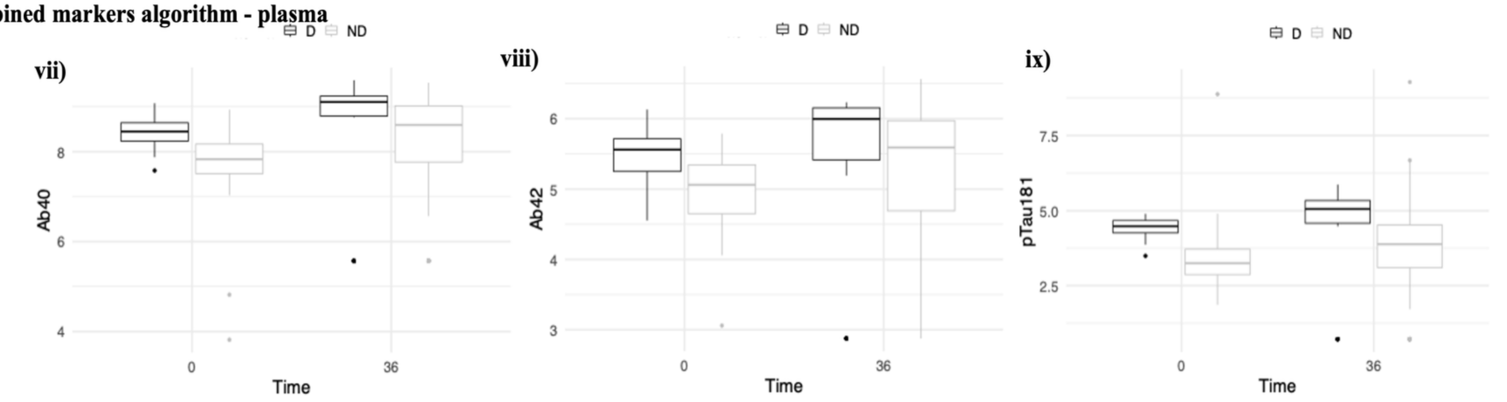

Supplemental Data: Precision Medicine for Alzheimer's Disease in Down Syndrome

b) **Boxplot of log-transformed values of the top 3 biomarkers predictive of cognitive status (demented [D] vs. non-demented [ND]) in aEV**

Proinflammatory markers algorithm - aEV

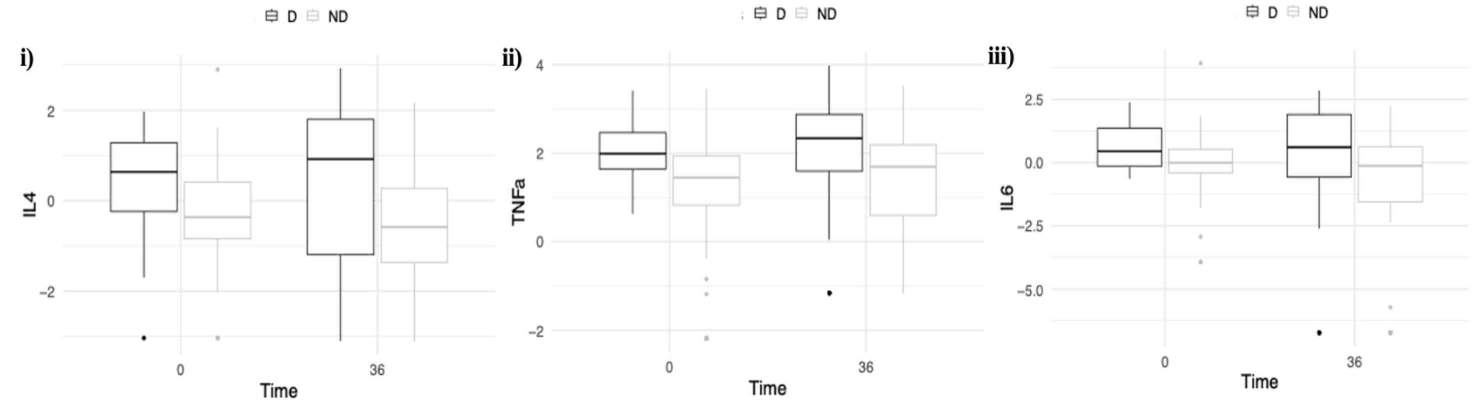

A/T/N markers algorithm - aEV

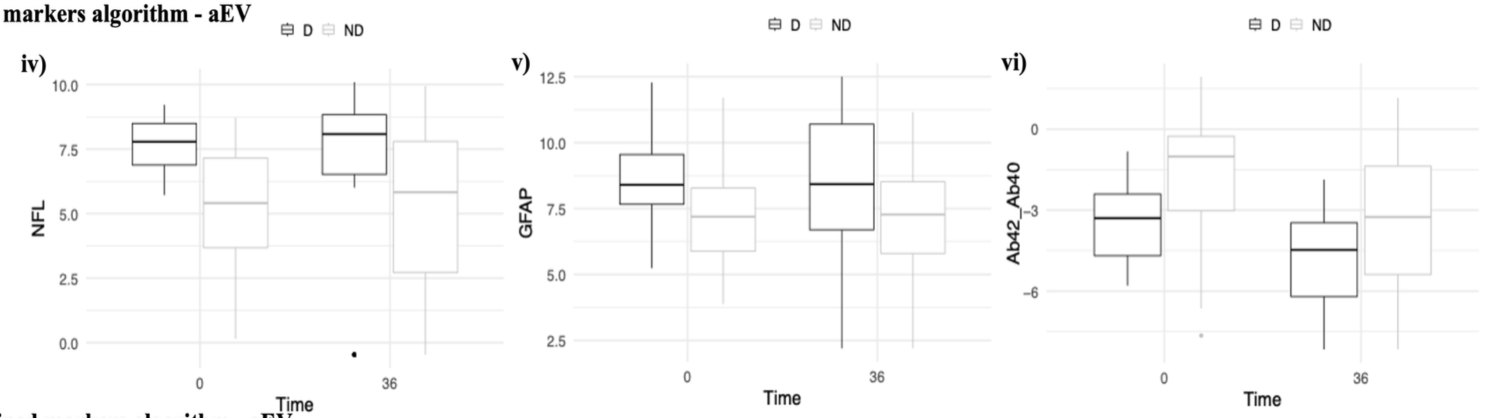

Combined markers algorithm - aEV

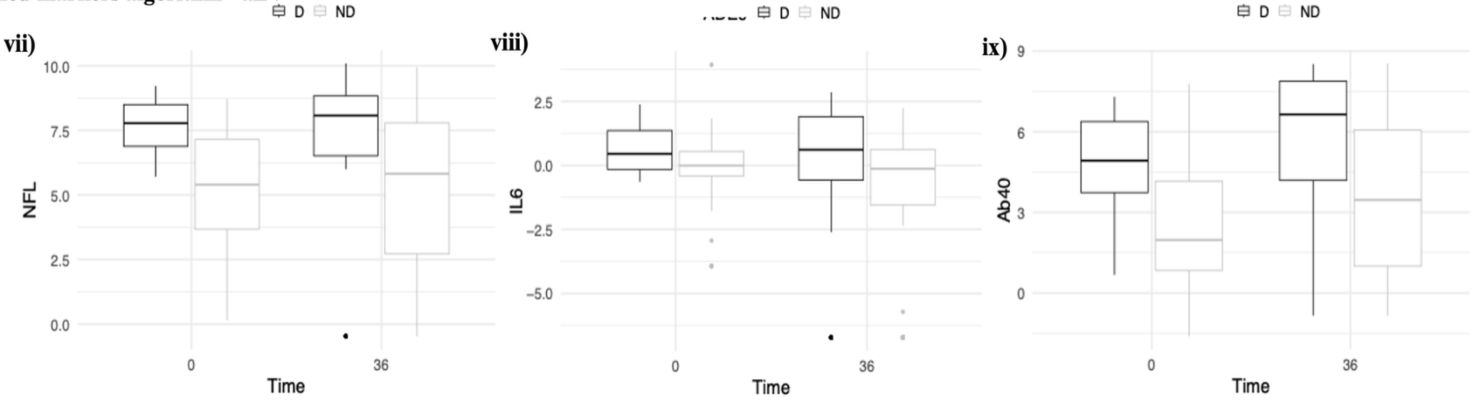

c) **<u>Boxplot of log-transformed values of the top 3 biomarkers predictive of cognitive status (demented [D] vs. non-demented [ND]) in nEV</u>**

Proinflammatory markers algorithm - nEV

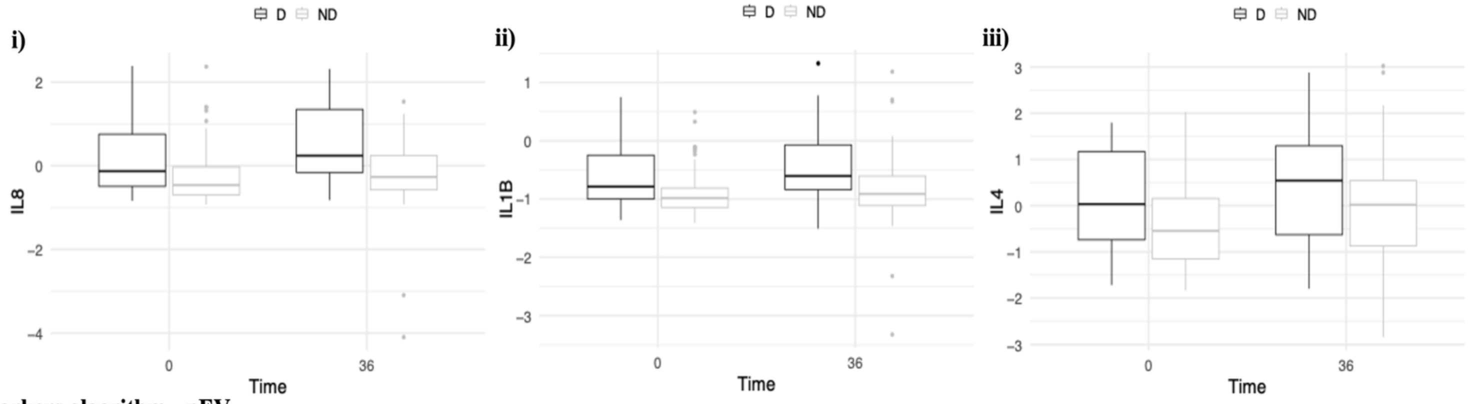

A/T/N markers algorithm - nEV

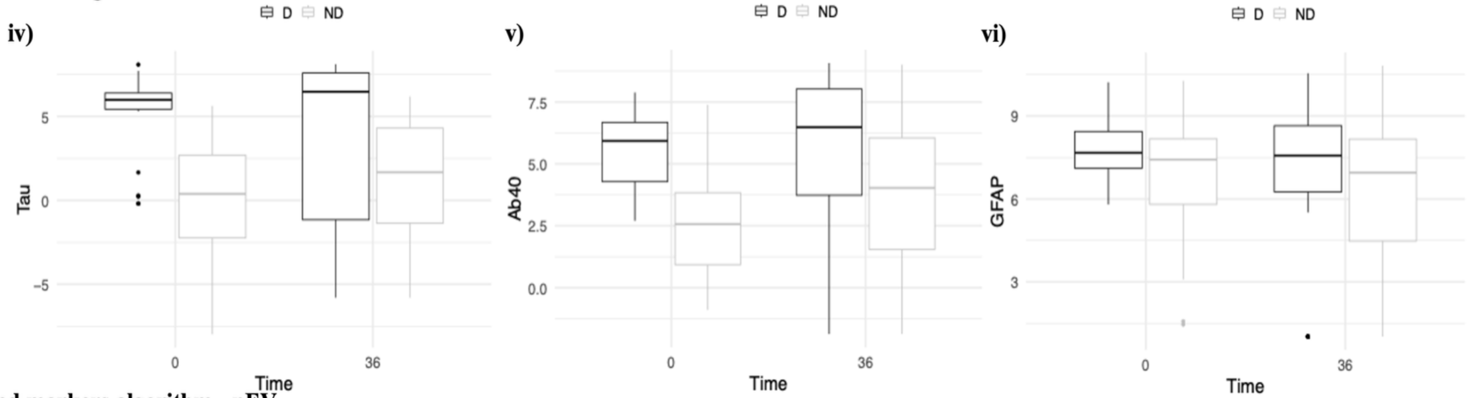

Combined markers algorithm - nEV

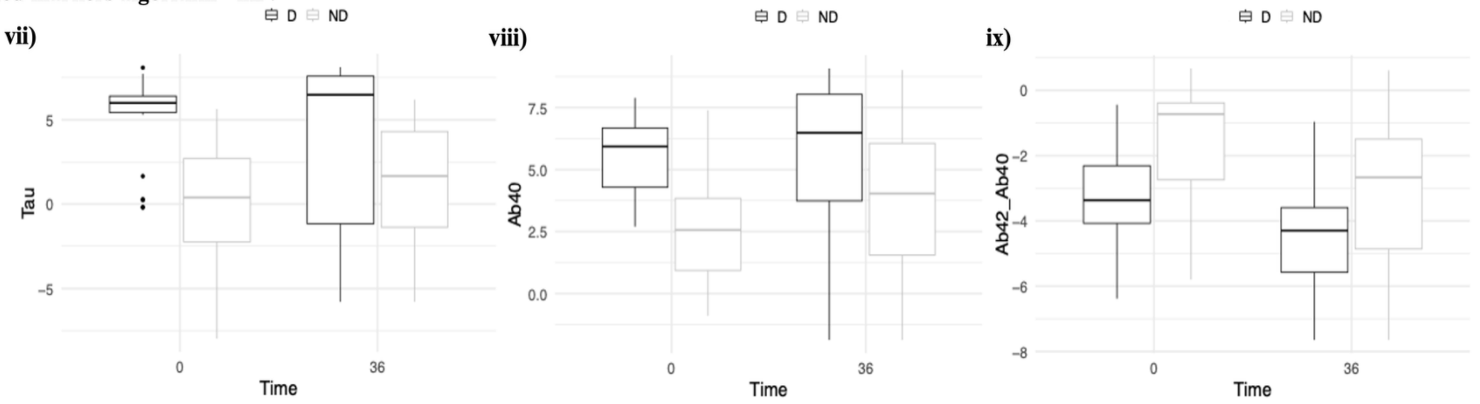

d) Boxplot of log-transformed values of the top 3 biomarkers predictive of treatment responders (yes vs. no) in plasma

Proinflammatory markers algorithm - plasma

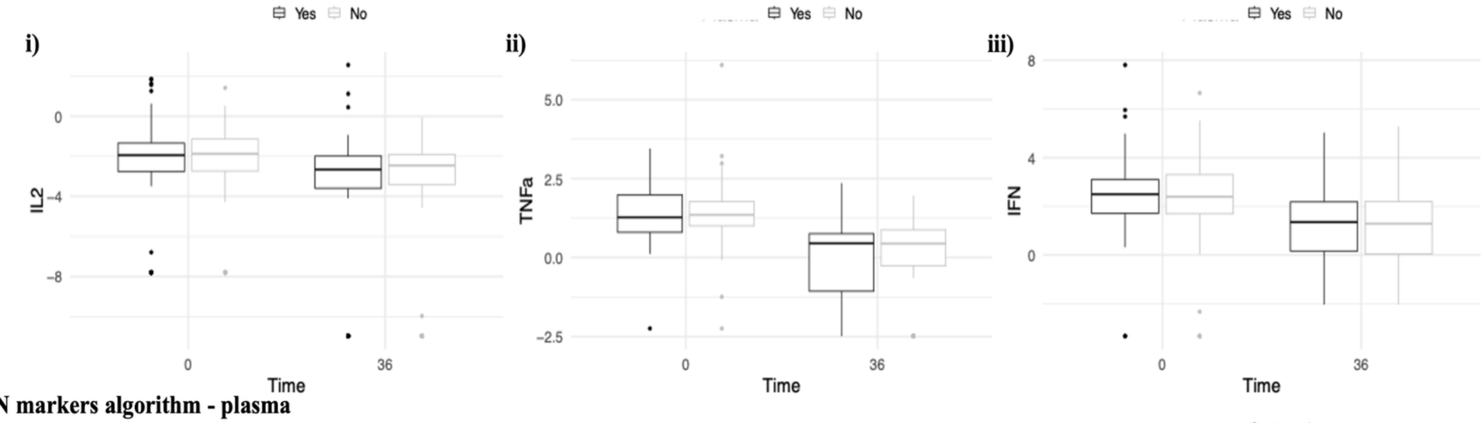

A/T/N markers algorithm - plasma

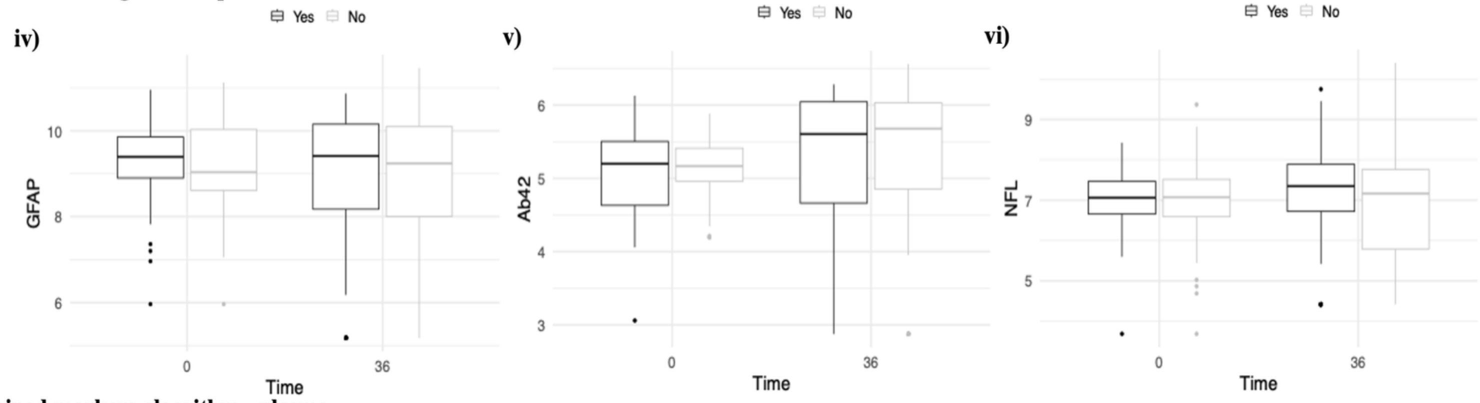

Combined markers algorithm - plasma

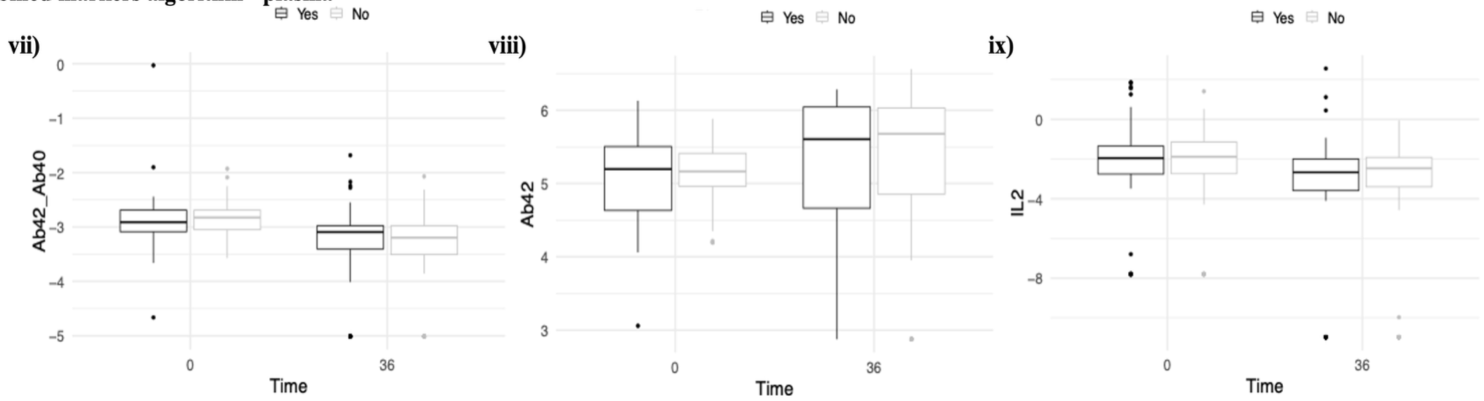

e) <u>Boxplot of log-transformed values of the top 3 biomarkers predictive of treatment responders (yes vs. no) in aEV</u>

Proinflammatory markers algorithm - aEV

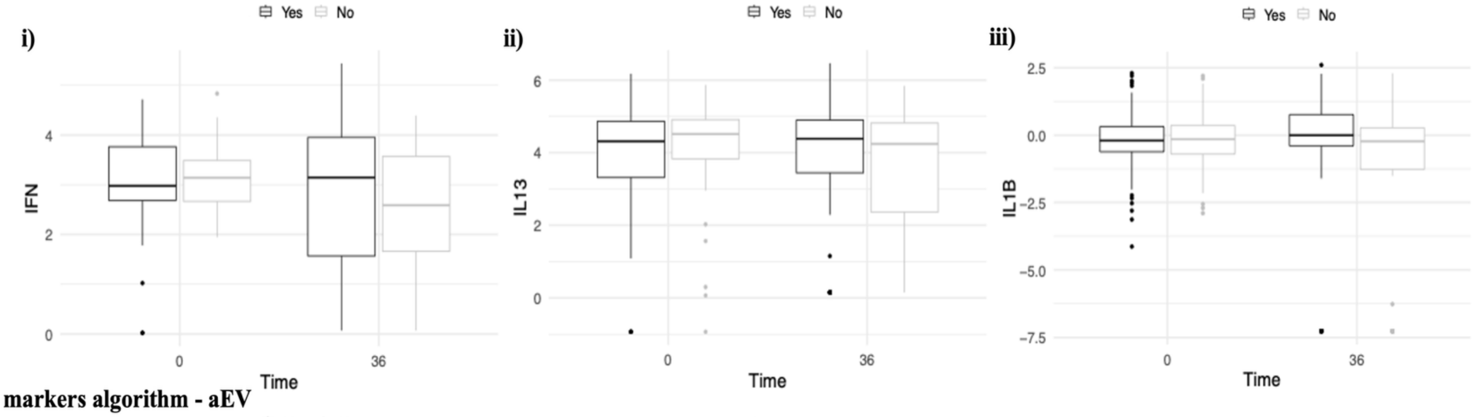

A/T/N markers algorithm - aEV

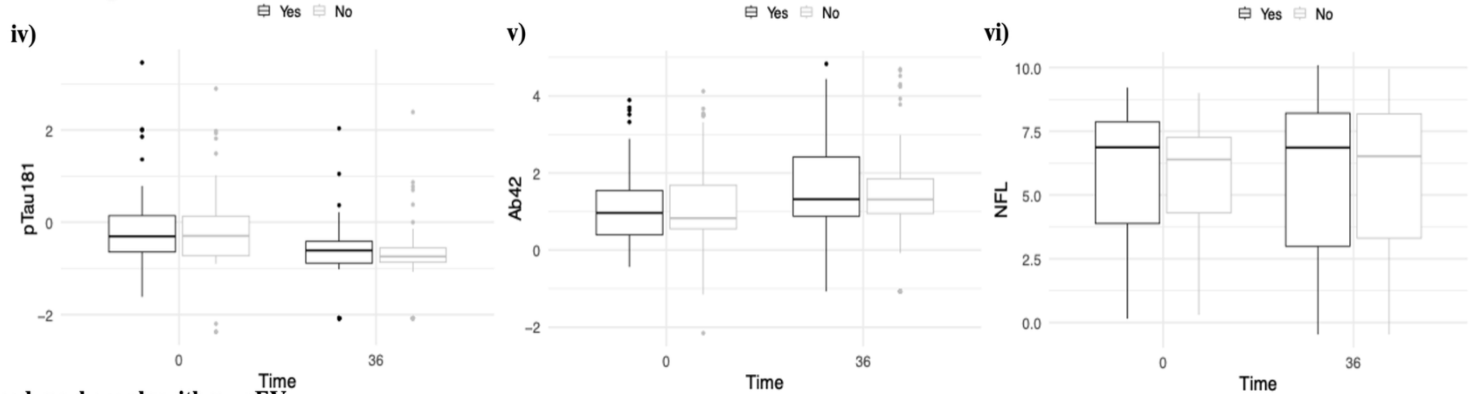

Combined markers algorithm - aEV

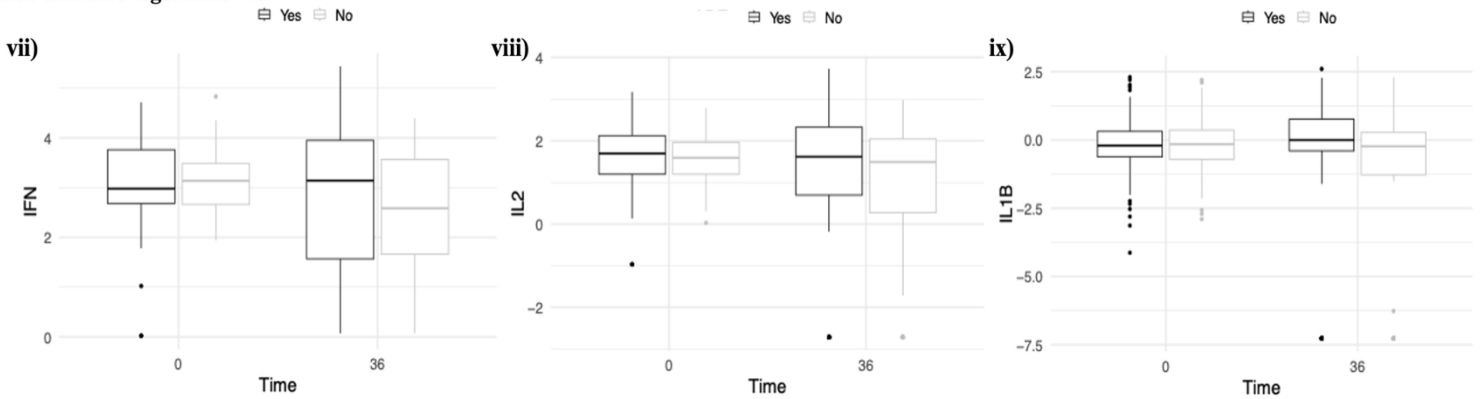

Supplemental Data: Precision Medicine for Alzheimer's Disease in Down Syndrome

f) Boxplot of log-transformed values of the top 3 biomarkers predictive of treatment responders (yes vs. no) in nEV

Proinflammatory markers algorithm - nEV

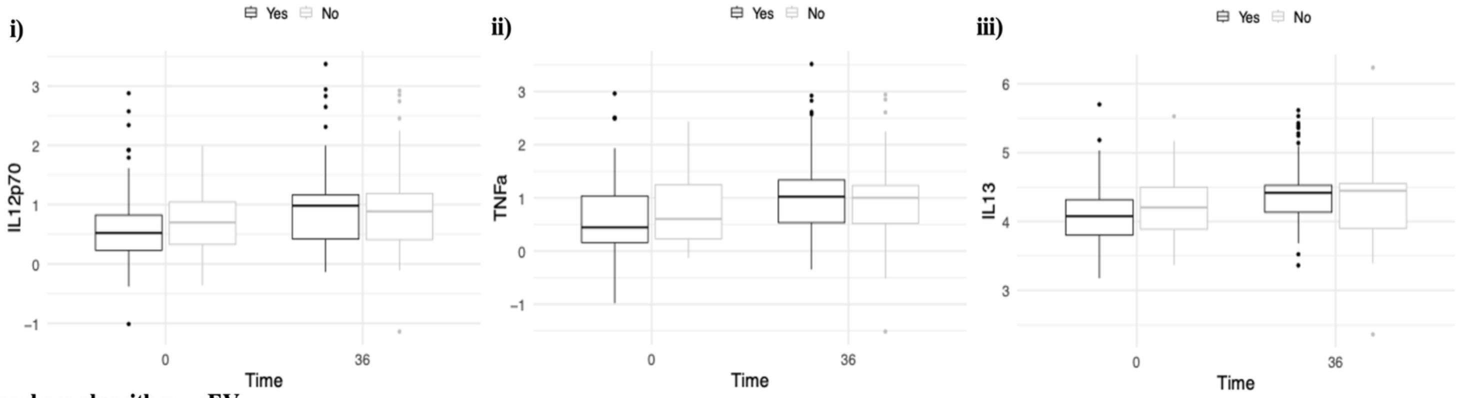

A/T/N markers algorithm - nEV

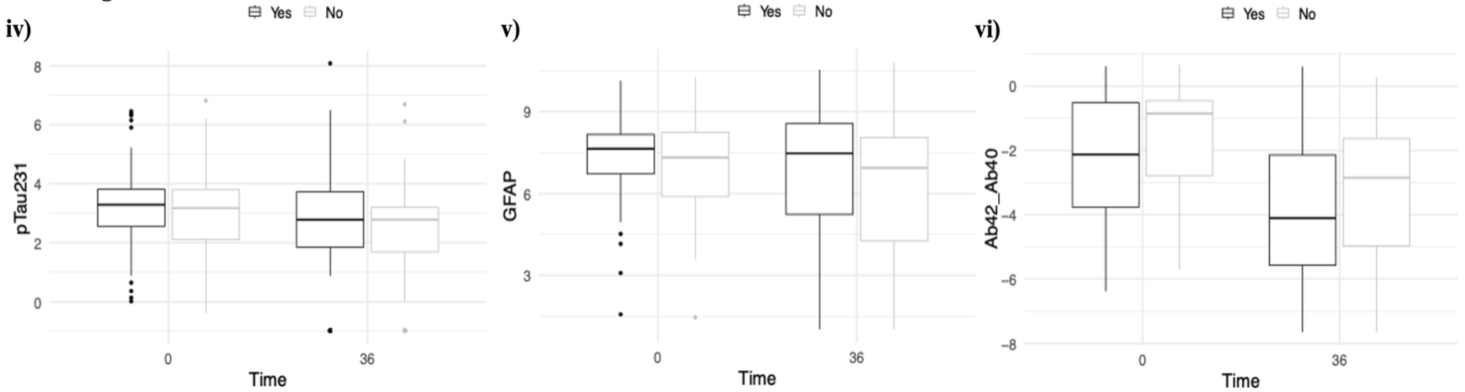

Combined markers algorithm - nEV

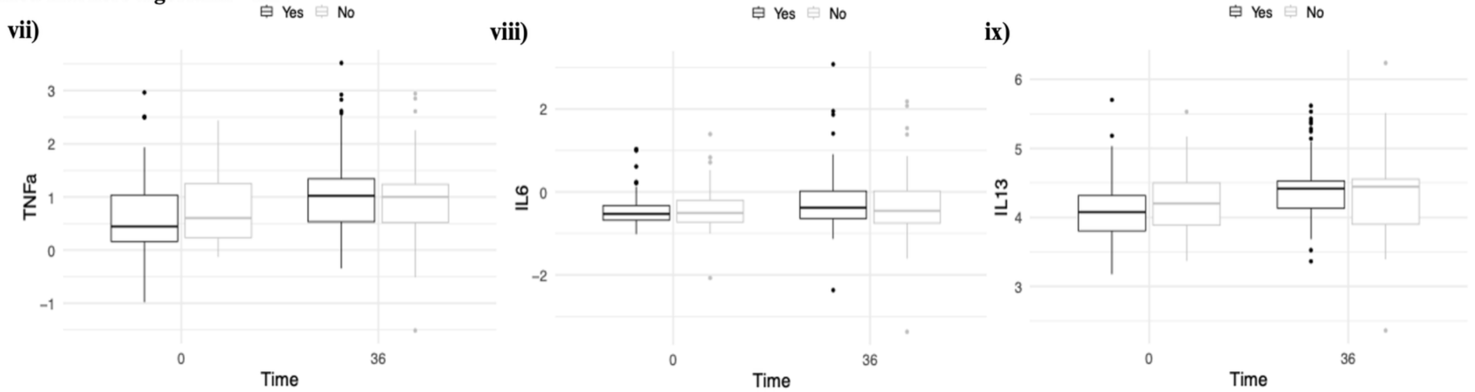

Supplemental Data: Precision Medicine for Alzheimer's Disease in Down Syndrome

g) **Boxplot of log-transformed values of the top 3 biomarkers predictive of change in treatment response (changed vs. no change) in plasma**

Proinflammatory markers algorithm - plasma

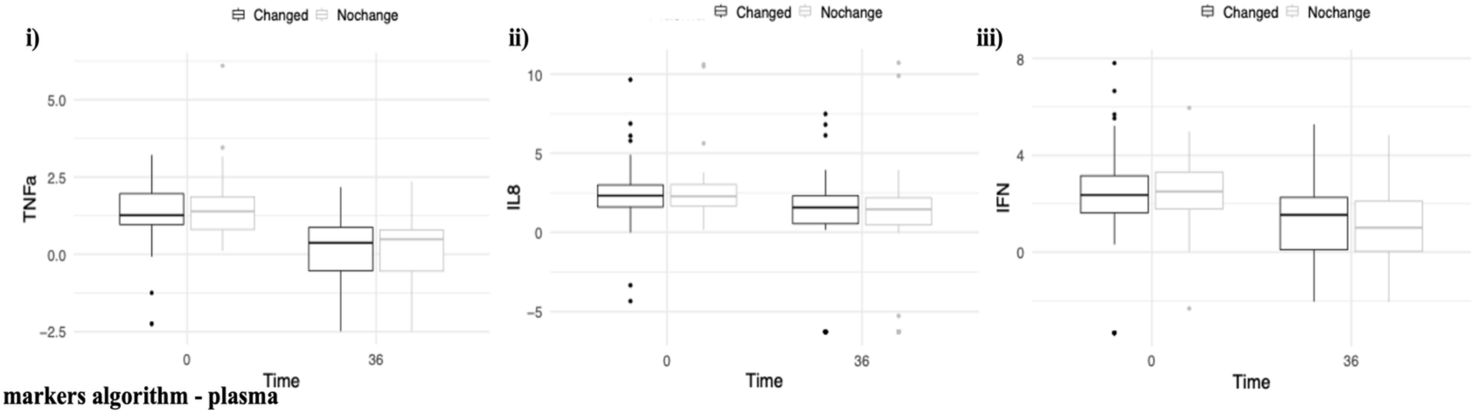

A/T/N markers algorithm - plasma

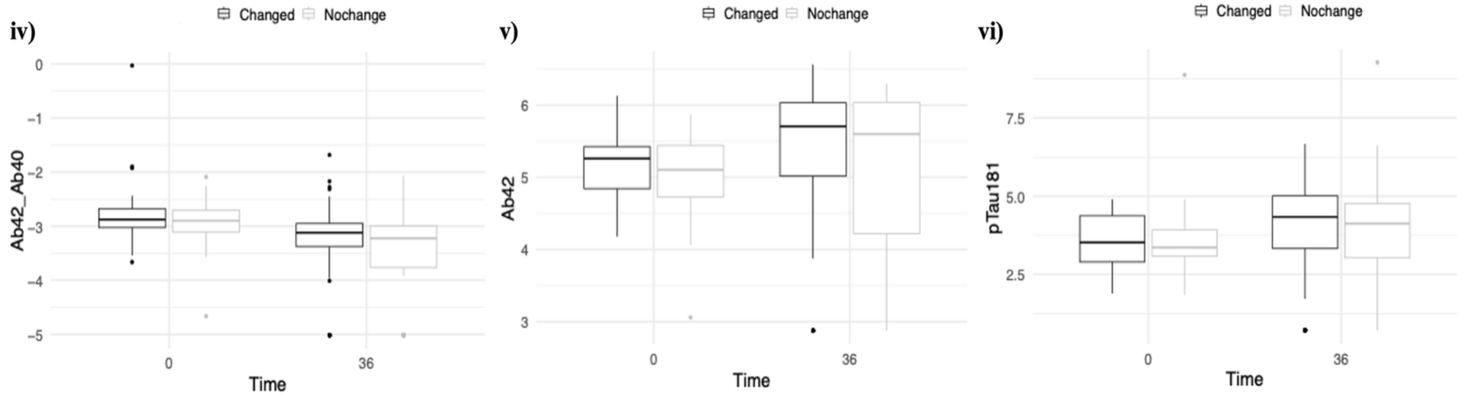

Combined markers algorithm - plasma

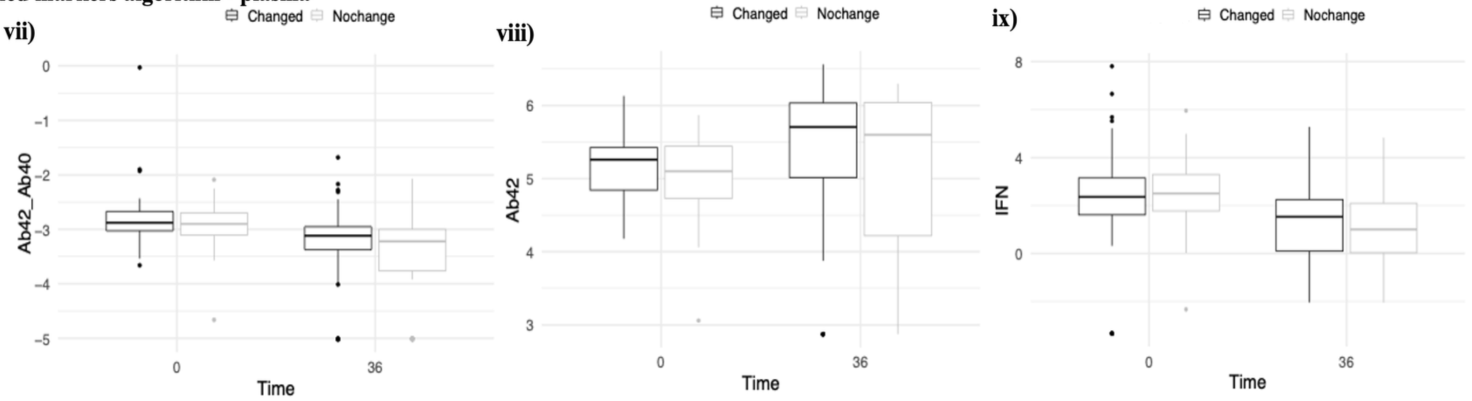

Supplemental Data: Precision Medicine for Alzheimer's Disease in Down Syndrome

h) Boxplot of log-transformed values of the top 3 biomarkers predictive of change in treatment response (changed vs. no change) in aEV

Proinflammatory markers algorithm - aEV

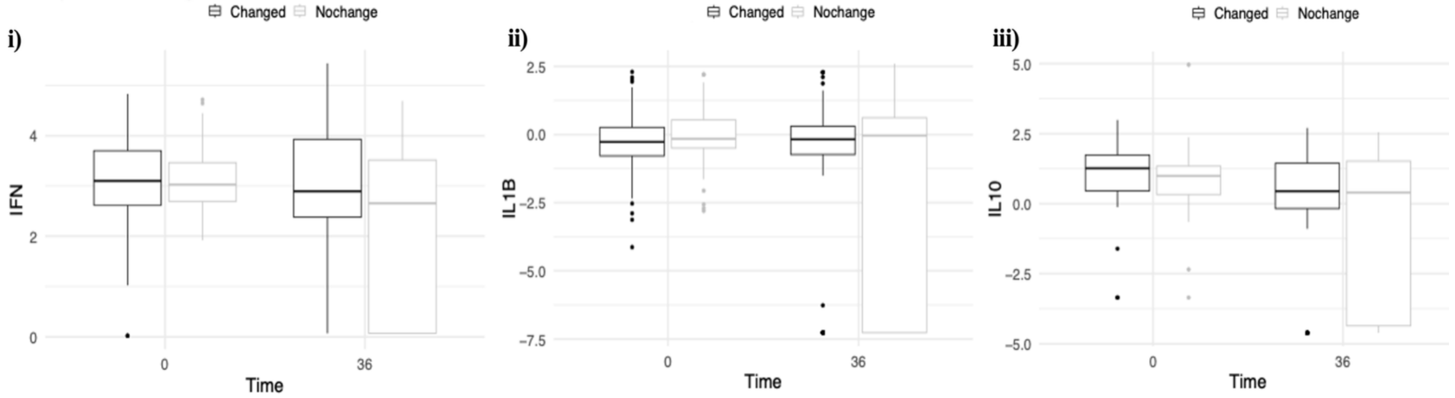

A/T/N markers algorithm - aEV

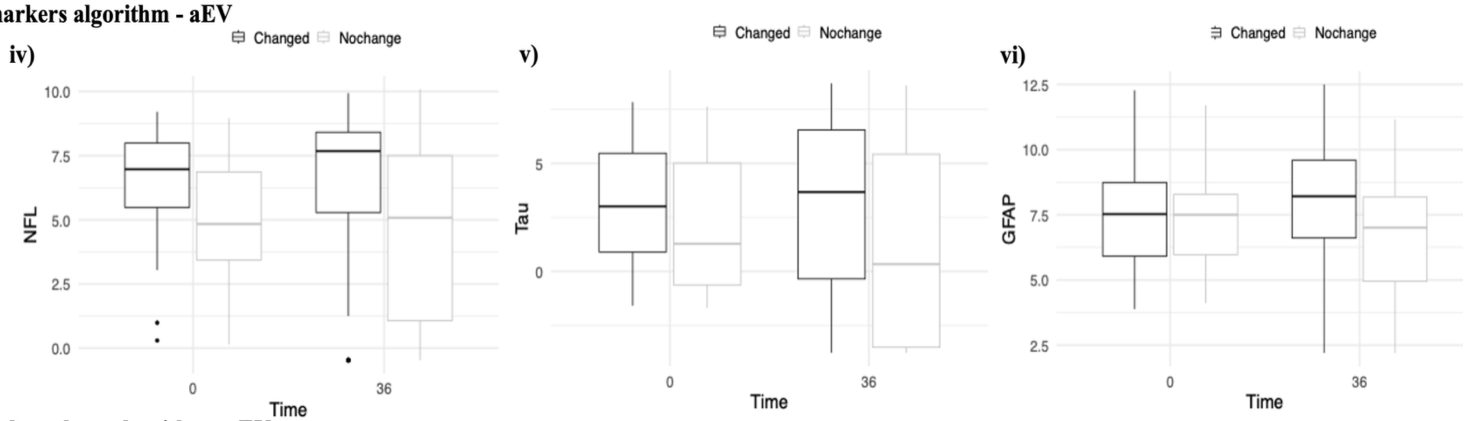

Combined markers algorithm - aEV

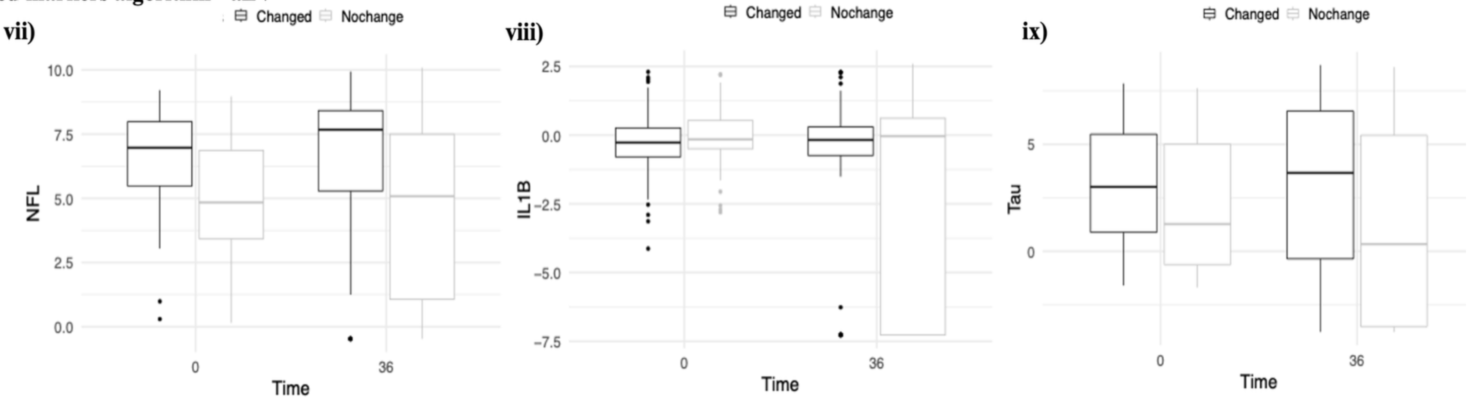

Supplemental Data: Precision Medicine for Alzheimer's Disease in Down Syndrome

i) Boxplot of log-transformed values of the top 3 biomarkers predictive of change in treatment response (changed vs. no change) in nEV

Proinflammatory markers algorithm - nEV

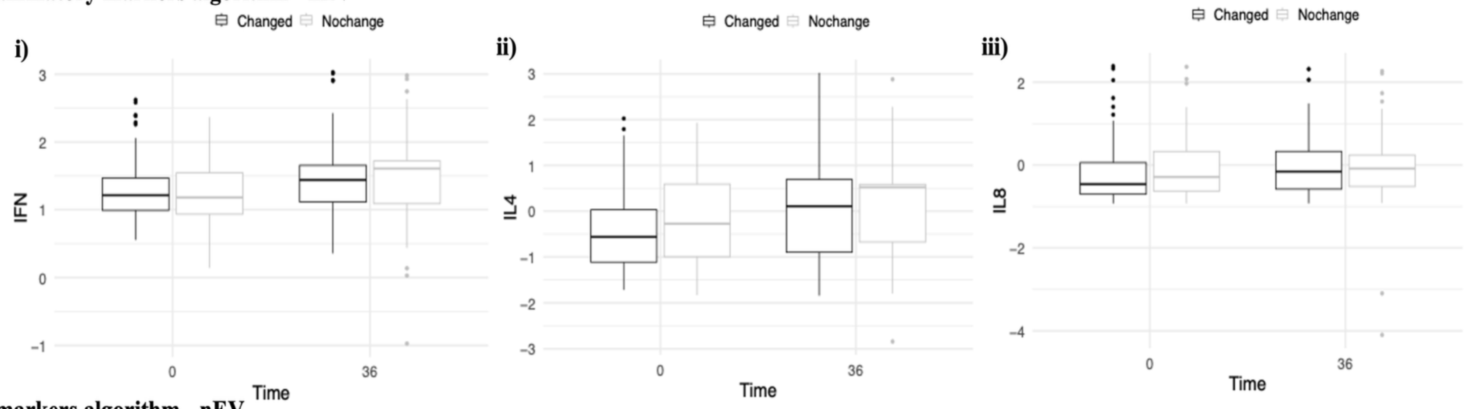

A/T/N markers algorithm - nEV

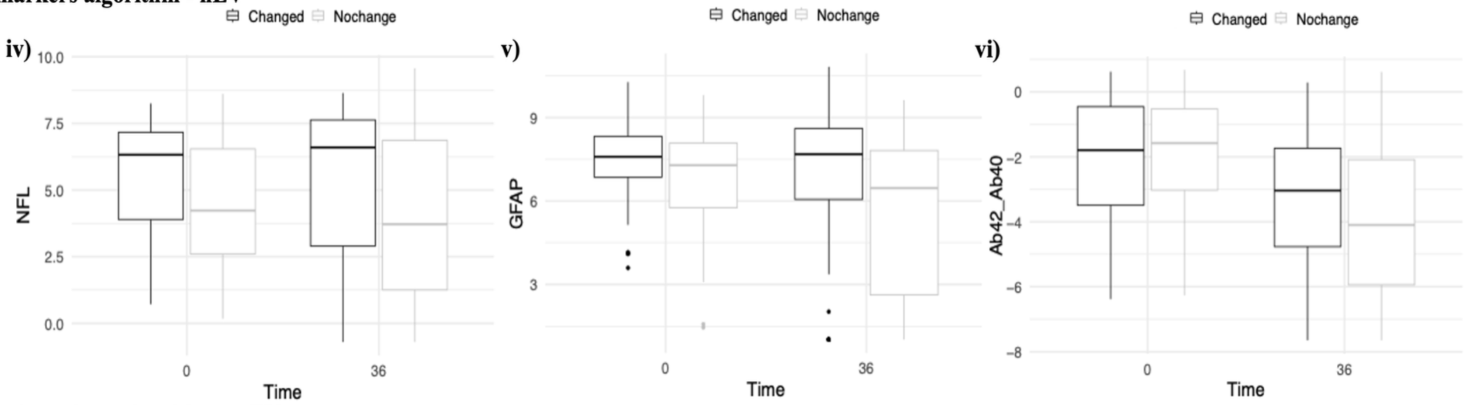

Combined markers algorithm - nEV

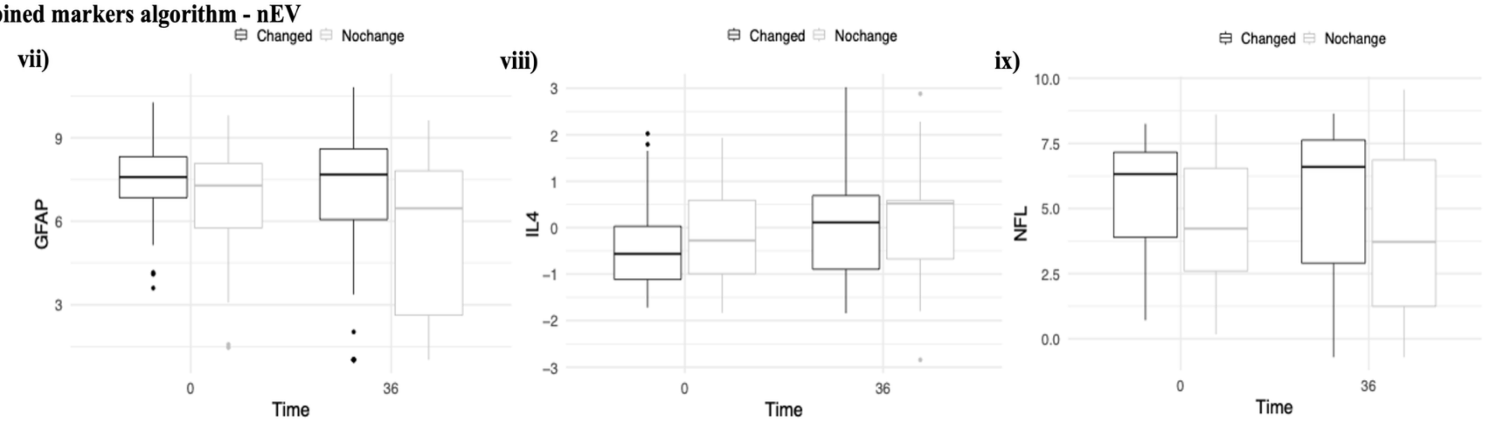

Supplement: Supplementary file 2 — Supporting Information [file ALZ-22-e71398-s002.pdf]
